# Genetic variability in cisplatin metabolic pathways and outcome of locally advanced head and neck squamous cell carcinoma patients

**DOI:** 10.1038/s41598-023-44040-7

**Published:** 2023-10-05

**Authors:** Ana Maria Castro Ferreira, João Maurício Carrasco Altemani, Ligia Traldi Macedo, Gustavo Jacob Lourenço, Carmen Silvia Passos Lima

**Affiliations:** 1 Laboratory of Cancer Genetics, Faculty of Medical Sciences, University of Campinas, Campinas, São Paulo Brazil; 2 Department of Anesthesiology, Oncology and Radiology, Faculty of Medical Sciences, University of Campinas, Rua Alexander Fleming, 181, Cidade Universitária “Zeferino Vaz”, Barão Geraldo, Campinas, São Paulo CEP: 13083-970 Brazil

**Keywords:** Head and neck cancer, Cancer genetics

## Abstract

Advanced head and neck squamous cell carcinoma (HNSCC) patients have been treated with cisplatin (CDDP) chemoradiation, and the variability of treatment effects has been attributed to single nucleotide variants (SNVs) in genes of metabolic pathways. This study investigated the roles of *GSTM1*, *GSTT1*, *GSTP1* c.313A>G, *XPC* c.2815A>C, *XPD* c.934G>A and c.2251A>C, *XPF* c.2505T>C, *ERCC1* c.354C>T, *MLH1* c.93G>A, *MSH2* c.211+9C>G, *MSH3* c.3133G>A, *EXO1* c.1765G>A, *TP53* c.215G>C, *CASP3* c.-1191A>G and c.-182-247G>T, *FAS* c.-1378G>A and c.-671A>G and *FASL* c.-844C>T SNVs in outcome of 109 patients treated with CDDP chemoradiation. Genotypes were identified in genomic DNA by PCR-based methods. Conventional criteria and tests analyzed response and survival. Patients with *XPC* c.2815AC or CC had 3.43 times more chances of presenting partial response or stable disease. Patients with *FAS* c.-671GG, *GSTM1* present plus *XPC* c.2815AA, or plus *XPD* c.934GG, or plus *XPD* c.2251AA, or plus *TP53* c.215GC or CC, and *XPD* c.2251AA plus *XPF* c.2505TT had up to 2.70 and 2.37 times more chances of presenting tumor progression and evolving to death, respectively. Our data indicate, for the first time, preliminary evidence that combined SNVs of CDDP metabolism act as independent prognostic factors and can be used to select patients for distinct treatments.

## Introduction

Cisplatin (CDDP)-based chemotherapy administered with radiotherapy (RT) remains the standard treatment for patients with locally advanced head and neck (HN) squamous cell carcinoma (SCC)^1,2^.

CDDP interacts mainly with DNA to form inter and intra-strand crosslinks^3^. The DNA lesions induced by CDDP activate different pathways, including intracellular detoxification^4,5^, DNA repair^5–7^, and apoptosis^5,8^.

Glutathione S-transferase proteins, encoded by mu 1 (*GSTM1*), theta 1 (*GSTT1*), and pi 1 (*GSTP1*) genes, conjugate CDDP to glutathione and contribute to its detoxification^4,5^. Proteins encoded by xeroderma pigmentosum C (*XPC*), D (*XPD*), F (*XPF*), excision repair cross-complementation group 1 (*ERCC1*), mutL homolog 1 (*MLH1*), mutS homolog 2 (*MSH2*), 3 (*MSH3*) and exonuclease 1 (*EXO1*) genes act on the nucleotide excision repair (NER) and the mismatch repair (MMR) pathways and remove DNA lesions of CDDP in cells^5–7^. When lesions in DNA overwhelm repair capacity, the biological effect favors apoptosis; the intrinsic and extrinsic apoptosis pathways are activated by modulation of enzymes encoded by tumor protein p53 (*TP53*), caspase 3 (*CASP3*), Fas cell surface death receptor (*FAS*) and Fas ligand (*FASL*) genes^5,8^ inducing damaged cells to death.

CDDP chemoradiation effects in HNSCC patients are variable, and this variability has been associated with single nucleotide variants (SNVs) on genes that act on CDDP metabolism pathways^9–18^. The functional roles of main SNVs^19–34^ are presented in Table 1.

**Table 1:** Functional roles of the wild and variant alleles of single nucleotide variant on genes enrolled in cisplatin metabolism.

SNV	Functional analyses				
	Wild allele	Function	Variant allele	Function	Reference
*GSTM1*	Present	ND	Null	Absent	^19^
*GSTT1*	Present	ND	Null	Absent	^20^
*GSTP1* c.313A>G	A	ND	G	RD	^21^
*XPC* c.2815A>C	A	NR	C	RR	^22^
*XPD* c.934G>A	G	NR	A	RR	^23^
*XPD* c.2251A>C	A	NR	C	RR	^23^
*XPF* c.2505 T>C	T	NR	C	RR	^24^
*ERCC1* c.354C>T	C	NR	T	RR	^25^
*MLH1* c.93G>A	G	NR	A	RR	^26^
*MSH2* c.211+9C>G	C	NR	G	RR	^27^
*MSH3 c*.3133G>A	G	NR	A	RR	^16^
*EXO1* c.1762G>A	G	NR	A	RR	^28^
*TP53* c.215G>C	G	NA	C	RA	^29^
*CASP3* c.-1191A>G	A	NA	G	RA	^30^
*CASP3* c.-182-247G>T	G	NA	T	RA	^31^
*FAS* c.-1378G>A	G	NA	A	RA	^32^
*FAS* c.-671A>G	A	NA	G	RA	^33^
*FASL* c.-844C>T	C	NA	T	RA	^34^

*SNV* single nucleotide variant, *ND* normal detoxification, *RD* reduced detoxification, *NR* normal repair, *RR* reduced repair, *NA* normal apoptosis, *RA* reduced apoptosis.

The *GSTM1*^11^, *XPD* c.934G>A^9–11^, *XPD* c.2251A>C^9,10,12^, *FAS* c.-671A>G^13^ and *FASL* c.-844C>T^13^ SNVs altered relapse-free (RFS), disease-free (DFS), event-free survival (EFS) and/or overall survival (OS) of HNSCC and oropharynx SCC patients treated with CDDP and/or RT, respectively.

Previous analyses of this prospective study conducted by our group demonstrated that *GSTP1* c.313A>G^17^, *XPD* c.934G>A^15^, *EXO1* c.1762G>A^15,16^, *FAS* c.-671A>G^18^ SNVs of single pathways influenced response rate (RR) and/or PFS, RFS, EFS, and OS of HNSCC patients treated with CDDP chemoradiation. As the final effects of treatment with CDDP chemoradiation possibly depend on the balance of actions of all CDDP metabolic pathways and not in single ways, the present analysis aimed to verify whether the association of defects involving intracellular detoxification, DNA repair, and apoptosis altered even more HNSCC patients´ outcome in almost the same cohort of patients.

## Materials and methods

### Patients and clinicopathological aspects

Previously published data on outcomes of HNSCC patients diagnosed at the Clinical Oncology Service of the General Hospital of the University of Campinas between June 2011 and February 2014, which focused on the roles of SNVs on genes of intracellular detoxification (n = 90)^17^, NER (n = 90)^15^, and MMR (n = 90)^16^, and intrinsic and extrinsic apoptosis (n = 109)^18^ pathways, were compiled and seen together in the current analysis of this prospective study. All patients were selected for CDDP chemoradiation as definitive treatment due to unresectable tumors, refusal of surgery related to expected sequels or an organ preservation protocol. Patients not candidates for CDDP treatment or under induction, adjuvant, or palliative therapy were excluded. Creatinine clearance greater than 45 ml/min was required.

The data related to age at diagnosis, gender, tobacco and alcohol consumption, histological grade, stage, human papillomavirus (HPV) type 16 status, and time to treatment delivery were obtained from the patient charts. As previously reported, patients were classified as smokers or non-smokers and drinkers or abstainers^35^. The tumor was diagnosed according to World Health Organization criteria^36^ and staged by the American Joint Committee of Cancer^37^. HPV was tested by P16 immunohistochemistry, as previously described^38,39^. The interval between the date of diagnosis and the date of treatment initiation was considered the time of treatment delivery.

The study was conducted according to the Declaration of Helsinki and approved by the University of Campinas Ethics Committees (no 274/2011; CAAE: 0218.0.146.000-11). Informed consent was obtained from all subjects and/or their legal guardians before the beginning of the study.

### Treatment, response rate, and survival

The single daily fractionated RT (70 Gy at 2 Gy/day) with concurrent bolus CDDP (80–100 mg/m^2^), given on days 1, 22, and 43, were administered to HNSCC patients; those with consistent side effects during treatment received CDDP at a lower dose (50–75 mg/m^2^)^16^. Patients who failed to respond to their initial treatment regimen or relapsed received intravenous methotrexate as palliative chemotherapy^40^. RR to CDDP chemoradiation was assessed as complete response (CR), partial response (PR,) or stable disease (SD), using Response Evaluation Criteria in Solid Tumors (RECIST) guidelines version 1.1^41^.

EFS was defined from the date of diagnosis and the date of progression, relapse, or death by disease. OS was determined from the date of diagnosis and death by any causes or last follow-up. Patients were followed at 3-month intervals, and the end of the follow-up period considered for the present study was June 2022.

### Genotyping

Genotyping was performed in DNA from peripheral blood samples of patients by multiplex polymerase chain reaction (PCR) for *GSTM1* and *GSTT1*^42^, PCR plus enzymatic digestion for *GSTP1* c.313A>G (rs1695)^43^, *XPC* c.2815A>C (rs2228001)^44^, *XPD* c.934G>A (rs1799793) and c.2251A>C (rs13181)^23^, *XPF* c.2505T>C (rs1799801)^45^, *ERCC1* c.354C>T (rs11615)^46^, *MLH1* c.93G>A (rs1800734)^47^, *MSH2* c.211+9C>G (rs2303426)^48^, *EXO1* c.1765G>A (rs1047840)^49^, *TP53* c.215G>C (rs1042522)^50^, *CASP3* c.-1191A>G (rs12108497)^30^ and c.-182-247G>T (rs4647601)^30^, *FAS* c.-1378G>A (rs2234767)^51^ and c.-671A>G (rs1800682)^52^, *FASL* c.-844C>T (rs763110)^51^ and real-time PCR for *MSH3* c.3133G>A (rs26279)^16^. PCR conditions and primers used are shown in Supplementary Table S1. Positive and negative controls were used in reactions. The 10% of genotype determinations were replicated in independent experiments with total concordance.

### Statistical analysis

Single SNVs and combinations of two SNVs with biological significance were analyzed in the study. To analyze the roles of deleterious isolated and combined genotypes related to normal or greater detoxification of CDDP, reduced apoptosis of cells damaged by CDDP, and regular or greater repair of lesions induced by CDDP based on information presented in Tables 1 and S2, in the outcome of HNSCC patients was the focus of the study. Differences between clinicopathological aspects and genotypes of SNVs in RR were analyzed by Fisher’s exact test in univariate Cox analysis. Only variables with more than ten individuals in each group and presenting *P*-values ≤ 0.20 were included in the multivariate Cox analysis, and the logistic regression model assessed associations of variables and RR, with odds ratios (OR) values and 95% confidence intervals (95% CI). Significant results were internally validated using a bootstrap resampling study to investigate the stability of risk estimates (1,000 replications). Kaplan–Meier method, log-rank test, and univariate and multivariate Cox analyses identified variables predicting EFS and OS, with hazard ratios (HR) values and 95% CI. All variables with *P*-value ≤ 0.20 in univariate Cox regression analysis were included in multivariate analysis.

All statistical tests were done using the SPSS 15.0 software (SPSS Incorporation, Chicago, IL, USA), and significance was achieved when *P*-values were ≤ 0.05.

## Results

### Clinicopathological aspects of patients

The median age at diagnosis of 109 HNSCC patients enrolled in the study was 56 years. Most of the patients were male, smokers, and drinkers, and had tumors located in the larynx and pharynx, moderately differentiated tumors, and tumors at advanced stages. Median BMI was within the ordinarily acceptable range, and HPV type 16 was negative in all analyzed cases (Table 2).

**Table 2:** Clinicopathological aspects of 109 patients with head and neck squamous cell carcinoma.

Variable	Patients
	Median (range) or N (%)
Age at diagnosis (years)	56 (27–74)
Gender	
Male	101 (92.7)
Female	8 (7.3)
Body mass index (kg/m^2^)	19.0 (13.0–31.5)
Tobacco consumption	
Smokers	106 (97.2)
Non-smokers	3 (2.28)
Alcohol consumption	
Drinkers	100 (91.7)
Abstainers	9 (8.3)
Tumor location	
Oral cavity	13 (11.9)
Pharynx	28 (25.7)
Larynx	68 (62.4)
Histological grade*	
Well-differentiated	3 (3.4)
Moderately differentiated	70 (78.7)
Poorly differentiated	15 (16.8)
Undifferentiated	1 (1.1)
Tumor stage	
I	1 (0.9)
II	5 (4.6)
III	17 (15.6)
IV	86 (78.9)
Human papillomavirus type 16*	
Positive	0 (0.0)
Negative	57 (100.0)
Average time to treatment delay (days)*	
≤ 123	65 (59.6)
> 123	42 (38.5)
Response rate*	
Complete	21 (23.9)
Partial	62 (70.4)
Stable disease	5 (5.7)

*N* number of patients.

*The number of patients differed from the total quoted in the study because it was not possible to obtain available tumor fragments and consistent information about the treatment and response rate in some cases.

### Response rate

The CR, PR, and SD were seen in 23.9%, 70.4%, and 5.7% of 88 available HNSCC patients, respectively.

All clinicopathological aspects and single and combined SNVs with biological significance in response to CDDP chemoradiation in 88 patients evaluated are presented in Supplementary Tables S3 and S4, respectively.

Factors with significant associations with the response rates to CDDP and RT are presented in Table 3. In univariate analysis, patients with T3 or T4 tumors, N2 or N3 nodal status, and *FAS* c.-1378GG genotype had 2.83, 4.77, and 2.67 times more chances of presenting PR or SD than those with T1 or T2 tumors, N1 or N2 nodal status and *FAS* c.-1378GA or AA genotype, respectively. In multivariate analysis, patients with T3 or T4 tumors, N2 or N3 nodal status, *XPC* c.2815AC or CC genotype had 3.05, 4.32, and 3.43 times more chances of presenting PR and SD than patients with the remaining aspects, respectively. Combined genotypes of analyzed SNVs did not consistently alter the response to CDDP chemoradiation.

**Table 3:** Clinicopathological aspects and single nucleotide variants in response to cisplatin chemoradiotherapy in 88 patients with head and neck squamous cell carcinoma.

Variable	Response rate						
	N	CR N (%)	PR or SD N (%)	Univariate analysis		Multivariate analysis	
				OR (95% CI)	*P* value	OR* (95%CI)	*P* value
Median age							
≤ 56 years	46	7 (33.3)	39 (58.2)	2.78 (0.99–7.79)	0.05	2.49 (0.81–7.52)	0.10
> 56 years	42	14 (66.7)	28 (41.8)	Reference		Reference	
Tumor size							
T1 or T2	23	9 (42.9)	14 (20.9)	Reference	0.05	Reference	**0.05**^a^
T3 or T4	65	12 (57.1)	53 (79.1)	2.83 (0.99–8.08)		**3.05 (0.97–9.60)**	
Nodal status							
N0 or N1	30	13 (61.9)	17 (25.4)	Reference	0.003	Reference	**0.008**^b^
N2 or N3	58	8 (38.1)	50 (74.6)	4.77 (1.69–13.50)		**4.32 (1.46–12.84)**	
*XPC* c.2815A>C							
AA	31	10 (47.6)	21 (31.3)	Reference	0.17	Reference	**0.04**^c^
AC or CC	57	11 (52.4)	46 (68.7)	1.99 (0.73–5.41)		**3.43 (1.02–11.48)**	
*FAS* c.-1378G>A							
GG	61	11 (52.4)	50 (74.6)	2.67 (0.96–7.40)	0.05	2.91 (0.90–9.38)	0.07
GA or AA	27	10 (47.6)	17 (25.4)	Reference		Reference	

*N* number of patients, *CR* complete response, *PR* partial response, *SD* stable disease.

*OR: odds ratio adjusted by age, tumor size, and nodal status. CI: confidence interval. The number of patients differed from the total quoted in the study because it was not possible to obtain consistent information about the response rate in some cases. Results with significant *P-*values (≤ 0.05) after multivariate analysis are presented in bold letters.

^a^*P*_boostrap_ = 0.02;

^b^*P*_bootstrap_ = 0.004;

^c^*P*_bootstrap_ = 0.03.

### Survival

The impact of all clinicopathological aspects and isolated and combined genotypes of detoxification, DNA repair, and apoptosis-related SNVs in the survival of 109 patients are presented in Supplementary Table S5.

Factors with significant associations with patients’ survival are presented in Table 4. The median follow-up time of HNSCC patients was 22 months (range: 3–126).

**Table 4:** Tumor aspects and detoxification, DNA-repair, and apoptosis-related single nucleotide variants in survival in 109 patients with head and neck squamous cell carcinoma.

Variables	Event-free survival					Overall survival				
	N of events/N total	Univariate analysis		Multivariate analysis*		N of events/N total	Univariate Cox analysis		Multivariate analysis*	
		*P* value	HR (95% CI)	*P* value	HR (95% CI)		P value	HR (95% CI)	P value	HR (95% CI)
Tumor size										
T1 or T2	16/27	**0.003**	Reference	**0.003**	Reference	18/27	**0.001**	Reference	**0.01**	Reference
T3 or T4	67/82		**2.33 (1.34–4.06)**		**2.33 (1.34–4.06)**	71/82		**3.39 (1.41–4.05)**		**1.94 (1.12–3.36)**
Tumor stage										
I or II	2/6	**0.03**	Reference	0.30	Reference	2/6	**0.02**	Reference	0.12	Reference
III or IV	81/103		**4.50 (1.09–18.53)**		2.23 (0.48–10.38)	87/103		**5.12 (1.25–21.03)**		3.16 (0.72–13.84)
Nodal status										
N1 or N2	28/42	0.08	Reference	0.12	Reference	31/42	0.08	Reference	0.31	Reference
N3 or N4	55/67		1.49 (0.94–2.36)		1.43 (0.90–2.26)	58/67		1.47 (0.95–2.28)		1.25 (0.80–1.97)
*XPD* c.934G>A										
GG	42/59	0.18	Reference	0.31	Reference	45/59	0.15	Reference	0.21	Reference
GA or AA	41/50		1.34 (0.87–2.07)		1.26 (0.80–1.98)	44/50		1.35 (0.89–2.06)		1.32 (0.32–2.05)
*XPD* c.2251A>C										
AA	38/55	0.10	Reference	0.11	Reference	41/55	0.14	Reference	0.12	Reference
AC or CC	45/54		1.44 (0.93–2.22)		1.42 (0.91–2.20)	48/54		1.36 (0.89–2.07)		1.40 (0.91-.2.14)
*ERCC1* c.354C>T										
CC	24/28	0.13	1.43 (0.89–2.31)	0.09	1.50 (0.93–2.42)	25/28	0.19	1.36 (0.85–2.16)	0.22	1.33 (0.83–2.13)
CT or TT	59/81		Reference		Reference	64/81		Reference		Reference
CC or CT	71/89	**0.04**	**1.89 (1.02–3.50)**	0.13	1.61 (0.86–3.00)	76/89	**0.03**	**1.92 (1.06–3.47)**	0.08	1.69 (0.93–3.08)
TT	12/20		Reference		Reference	13/20		Reference		Reference
*EXO1* c.1765G>A										
GG	37/45	0.08	1.46 (0.94–2.26)	0.09	1.44 (0.93–2.24)	39/45	0.20	1.31 (0.86–2.00)	0.22	1.29 (0.85–1.97)
GA or AA	46/64		Reference		Reference	50/64		Reference		Reference
*CASP3* c.-1191A>G										
GG	6/11	0.18	Reference	0.34	Reference	8/11		Reference	0.85	Reference
AA or AG	77/98		1.75 (0.76–4.02)		1.50 (0.64–3.49)	81/98	0.51	1.27 (0.61–2.63)		1.07 (0.51–2.24)
*FAS* c.-671A>G										
GG	22/26	0.09	1.52 (0.93–2.48)	**0.006**	**2.03 (1.22–3.35)**	66/83	0.08	1.51 (0.94–2.44)	**0.006**	**1.97 (1.21–3.20)**
AG or AA	61/83		Reference		Reference	23/26		Reference		Reference
GG or AG	59/75	0.44	Reference	0.12	Reference	65/75	0.15	1.40 (0.88–2.25)	**0.04**	**1.62 (1.00–2.63)**
AA	24/34		1.20 (0.74–1.93)		1.47 (0.89–2.41)	24/34		Reference		Reference
*GSTM1* + *XPC* c.2815A>C										
Present + AA	33/40	**0.03**	**2.19 (1.07–4.48)**	**0.008**	**2.70 (1.29–5.66)**	34/40	**0.05**	**1.89 (0.97–3.68)**	**0.04**	**1.99 (1.01–3.92)**
Null + AC or CC	10/20		Reference		Reference	12/20		Reference		Reference
*GSTMI* + *XPD* c.934G>A										
Present + GG	24/27	**0.03**	**2.06 (1.07–3.96)**	**0.01**	**2.45 (1.17–5.13)**	26/27	**0.02**	**1.99 (1.07–3.69)**	**0.01**	**2.37 (1.17–4.79)**
Null + GA or AA	16/25		Reference		Reference	18/25		Reference		Reference
*GSTMI* + *XPD* c.2251A>C										
Present + AA	26/30	**0.02**	**2.17 (1.10–4.25)**	**0.05**	**1.93 (0.98–3.77)**	28/31	**0.04**	**1.93 (1.02–3.62)**	**0.04**	**1.90 (1.01–3.57)**
Null + AC or CC	14/24		Reference		Reference	16/24		Reference		Reference
*GSTMI* + *XPF* c.2505 T>C										
Present + TT	26/31	**0.04**	**2.00 (1.01–3.92)**	**0.03**	**2.09 (1.06–4.12)**	26/31	0.08	1.75 (0.91–3.35)	0.07	1.79 (0.93–3.46)
Null + TC or CC	22/31		Reference		Reference	15/22		Reference		Reference
*GSTMI* + *MLH1* c.93G>A										
Present + GG	21/29	0.25	1.43 (0.77–2.66)	0.17	1.53 (0.82-.2.87)	25/29	0.15	1.52 (0.85–2.73)	0.09	1.66 (0.92–2.98)
Null + GA or AA	20/31		Reference		Reference	22/31		Reference		Reference
*GSTMI* + *TP53* c.215G>C										
Present + CC or GC	24/30	0.19	1.51 (0.81–2.82)	**0.04**	**1.98 (1.01–3.85)**	25/30	0.31	1.35 (0.74–2.46)	**0.04**	**1.93 (1.01–3.68)**
Null + GG	17/27		Reference		Reference	19/27		Reference		Reference
*GSTMI* + *CASP3* c.-1191A>G										
Present + GG or AG	20/22	**0.04**	**1.95 (1.00–3.79)**	**0.04**	**1.95 (1.00–3.79)**	20/22	**0.04**	**1.96 (1.03–3.74)**	0.06	1.85 (0.97–3.55)
Null + AA	16/25		Reference		Reference	18/25		Reference		Reference
*GSTMI* + *FAS* c.-1378G>A										
Present + AA	48/59	0.33	1.78 (0.55–5.77)	0.46	1.58 (0.46–5.35)	51/59	0.19	2.19 (0.67–7.06)	0.11	2.60 (0.80–8.44)
Null + GA or GG	3/5		Reference		Reference	3/5		Reference		Reference
Present + AA or GA	35/42	0.33	1.50 (0.66–3.39)	0.17	1.76 (0.77–3.62)	37/42	0.19	1.70 (0.75–3.83)	0.15	1.81 (0.80–4.09)
Null + GG	8/10		Reference		Reference	8/10		Reference		Reference
*GSTTI* + *EXO1* c.1762G>A										
Present + GG	6/9	0.17	Reference	0.28	Reference	7/9		Reference		Reference
Null + GA or AA	30/37		1.84 (0.76–4.44)		1.65 (0.66–4.10)	32/37	0.27	1.58 (0.69–3.62)	0.54	1.30 (0.55–3.07)
*GSTTI* + *CASP3* c.-182-247G>T										
Present + TT or GT	2/5	0.14	Reference	0.25	Reference	3/5		Reference	0.52	Reference
Null + GG	45/58		2.86 (0.69–11.83)		2.28 (0.54–9.50)	44/58	0.28	1.91 (0.59–6.16)		1.47 (0.45–4.76)
*GSTPI* c.313A>G + *EXO1* c.1762G>A										
AA + GG	24/32	0.09	Reference	0.06	Reference	25/32	0.13	Reference	0.09	Reference
AG or GG + GA or AA	18/22		1.69 (0.91–3.14)		1.81 (0.95–3.42)	19/22		1.57 (0.86–2.86)		1.69 (0.91–3.12)
*XPC* c.2815A>C + *XPD* c.934G>A										
AA + GG	27/34	0.07	1.88 (0.95–3.73)	0.16	1.63 (0.81–3.27)	31/34	0.14	1.56 (0.86–2.83)	0.27	1.40 (0.76–2.56)
AC or CC + GA or AA	13/25		Reference		Reference	18/25		Reference		Reference
*XPC* c.2815A>C + *XPD* c.2251A>C										
AA + AA	31/37	**0.05**	**1.93 (0.99–3.77)**	0.07	1.85 (0.94–3.62)	34/37	0.17	1.51 (0.83–2.75)	0.20	1.47 (0.81–2.68)
AC or CC + AC or CC	13/24		Reference		Reference	17/24		Reference		Reference
*XPC* c.2815A>C + *XPF* c.2505 T>C										
AA + TT	34/40	0.12	1.62 (0.88–2.99)	0.09	1.69 (0.91–3.13)	33/40	0.26	1.39 (0.77–2.52)	0.19	1.48 (0.81–2.70)
AC or CC + TC or CC	15/24		Reference		Reference	17/24		Reference		Reference
*XPD* c.934G>A + *XPD* c.2251A>C										
GG + AA	35/42	0.10	1.49 (0.91–2.42)	0.13	1.46 (0.88–2.42)	37/42	0.12	1.45 (0.90–2.32)	0.09	1.51 (0.92–2.47)
GA or AA + AC or CC	32/47		Reference		Reference	34/47		Reference		Reference
*XPD* c.934G>A + *XPF* c.2505 T>C										
GG + TT	24/27	**0.05**	**1.83 (0.99–3.38)**	0.06	1.78 (0.97–3.29)	25/27	**0.03**	**1.86 (1.03–3.34)**	**0.01**	**2.04 (1.12–3.71)**
AG or AA + TC or CC	20/29		Reference		Reference	23/29		Reference		Reference
*XPD* c.934G>A + *MLH1* c.93G>A										
GG + GG	17/20	0.12	1.62 (0.86–3.05)	0.28	1.41 (0.75–2.66)	20/20	0.06	1.75 (0.96–3.19)	0.08	1.69 (0.92–3.09)
GA or AA + GA or AA	25/33		Reference		Reference	26/33		Reference		Reference
*XPD* c.934G>A + *MSH2* c.211 + 9C>G										
GG + CC	33/41	0.25	1.49 (0.75–2.97)	0.49	1.28 (0.63–2.59)	36/41	0.17	1.59 (0.80–3.14)	0.47	1.28 (0.64–2.55)
GA or AA + GC or GG	11/16		Reference		Reference	11/16		Reference		Reference
*XPD* c.2251A>C + *XPF* c.2505 T>C										
AA + TT	24/28	**0.04**	**1.98 (1.03–3.80)**	**0.03**	**2.00 (1.04–3.86)**	25/28	**0.05**	**1.84 (0.99–3.40)**	**0.04**	**1.87 (1.01–3.47)**
AC or CC + TC or CC	16/26		Reference		Reference	19/26		Reference		Reference
*XPD* c.2251A>C + *MLH1* c.93G>A										
AA + GG	18/22	0.14	1.59 (0.84–3.00)	0.17	1.54 (0.81–2.92)	21/22	0.11	1.63 (0.89–2.97)	0.06	1.75 (0.96–3.21)
AC or CC + GA or AA	22/31		Reference		Reference	23/31		Reference		Reference
*XPD* c.2251A>C + *MSH2* c.211 + 9C>G										
AA + CC	35/43	0.17	1.67 (0.80–3.48)	0.32	1.46 (0.68–3.10)	38/43	0.14	1.71 (0.82–3.55)	0.42	1.34 (0.64–2.81)
AC or CC + GC or GG	9/14		Reference		Reference	9/14		Reference		Reference
*XPD* c.2251A>C + *CASP3* c.-1191A>G										
AA + GG	42/49	0.13	2.47 (0.76–8.00)	0.13	2.47 (0.76–8.00)	44/49	0.29	1.73 (0.62–4.84)	0.77	1.17 (0.40–3.37)
AC or CC + AG or AA	3/6		Reference		Reference	4/6		Reference		Reference
*XPF* c.2505 T>C + *MSH3* c.3133G>A										
TT + GG	43/54	0.84	1.10 (0.43–2.79)	0.48	1.42 (0.53–3.81)	45/54	0.18	1.99 (0.71–5.57)	0.43	1.51 (0.53–4.29)
TC or CC + GA or AA	5/7		Reference		Reference	4/7		Reference		Reference
*ERCC1* c.354C>T + *MSH3* c.3133G>A										
CC or CT + GG or GA	8/14	0.06	Reference	0.21	Reference	8/14	**0.02**	Reference	0.09	Reference
TT + AA	32/42		2.12 (0.96–4.69)		1.69 (0.73–3.89)	35/42		**2.38 (1.09–5.20)**		1.99 (0.89–4.46)
*ERCC1* c.354C>T + *FAS* c.-1378G>A										
CC or CT + AA or GA	10/16	0.18	Reference	0.90	Reference	11/16	0.17	Reference	0.55	Reference
TT + GG	21/25		1.66 (0.78–3.55)		1.05 (0.44–2.49)	22/25		1.64 (0.79–3.41)		1.27 (0.57–2.84)
*ERCC1* c.354C>T + *FASL* c.-844C>T										
CC or CT + TT or CT	4/9	**0.02**	Reference	**0.05**	Reference	6/9	0.08	Reference	0.15	Reference
TT + CC	52/65		**3.11 (1.12–8.63)**		**2.69 (0.96–7.53)**	55/66		2.10 (0.89–4.90)		1.86 (0.79–4.37)
*MSH3* c.3133G > A + *FAS* c.-671A>G										
GG or GA + GG or AG	11/17	0.46	Reference	0.46	Reference	11/17	0.18	Reference	0.20	Reference
AA + AA	23/31		1.31 (0.63–2.70)		1.31 (0.63–2.72)	27/31		11.62 (0.80–3.28)		1.58 (0.77–3.23)
*TP53* c.215G>C + *FAS* c.-671A>G										
CC or CG + AG or GG	13/17		Reference	0.44	Reference	12/17	0.19	Reference	0.24	Reference
GG + AA	32/41	0.63	1.16 (0.61–2.23)		1.30 (0.66–2.54)	35/41		1.53 (0.79–2.97)		1.50 (0.76–2.96)

*N* number of patients, *HR* hazard ratio, *CI* confidence interval;

*Multivariate analysis adjusted by tumor size, tumor stage and nodal status; Results with significant *P*-values are presented in bold letters. Only single and combined genotypes with *P*-value < 0.20 in univariate analysis were included in multivariate analysis.

At 24 months of follow-up, EFS was lower in patients with specific tumor aspects and genotypes (Kaplan–Meier estimates). In univariate Cox analysis, patients with T3 or T4 tumors, tumors at III or IV stage, *ERCC1* c.354CC or CT, *GSTM1* present plus *XPC* c.2815AA, *GSTM1* present plus *XPD* c.934GG, *GSTM1* present plus *XPD* c.2251AA, *GSTM1* present plus *XPF* c.2505TT, *GSTM1* present plus *CASP3* c.-1191GG or AG, *XPC* c.2815AA plus *XPD* c.2251AA, *XPD* c.934GG plus *XPF* c.2505TT, *XPD* c.2251AA plus *XPF* c.2505TT, and *ERCC1* c.354TT plus *FASL* c.-844CC had up to 4.50 times more chances of presenting progression, relapse, or death by disease effects than others. In multivariate analysis, patients with T3 or T4 tumor, *FAS* c.-671GG, *GSTM1* present plus *XPC* c.2815AA, *GSTM1* present plus *XPD* c.934GG, *GSTM1* present plus *XPD* c.2251AA, *GSTM1* present plus *XPF* c.2505TT, *GSTM1* present plus *TP53* c.215CC or GC, *GSTM1* present plus *CASP3* c.-1191GG or AG, *XPD* c.2251AA plus *XPF* c.2505TT, and *ERCC1* c.354TT plus *FASL* c.-844CC had up to 2.69 times more chances of presenting progression, relapse, or death by disease effects than others.

OS was lower in patients with specific tumor aspects and genotypes at 24 months of follow-up (Kaplan–Meier estimates). Lower OS was also observed in patients with specific tumor aspects and genotypes at 24 months of follow-up (Kaplan–Meier estimates). In univariate Cox analysis, patients with T3 or T4 tumors, tumors at III or IV stage, *ERCC1* c.354CC or CT, *GSTM1* present plus *XPC* c.2815AA, *GSTM1* present plus *XPD* c.934GG, *GSTM1* present plus *XPD* c.2251AA, *GSTM1* present plus *CASP3* c.-1191GG or AG, *XPD* 934GG plus *XPF* c.2505TT, *XPD* c.2251AA plus *XPF* c.2505TT, and *ERCC1* c.354TT plus *MSH3* c.3133AA had up to 5.12 times more chances of evolving to death by any cause than remaining patients. In multivariate analysis, patients with T3 or T4 tumor, *FAS* c.-671GG, *FAS* c.-671GG or AG, *GSTM1* present plus *XPC* c.2815AA, *GSTM1* present plus *XPD* c.934GG, *GSTM1* present plus *XPD* c.2251AA, *GSTM1* present plus *TP53* c.215CC or GC, *XPD* c.934GG plus *XPF* c.2505TT, and *XPD* c.2251AA plus *XPF* c.2505TT had up to 2.37 times more chances of evolving to death by any cause than the remaining patients.

**Figure 1:**
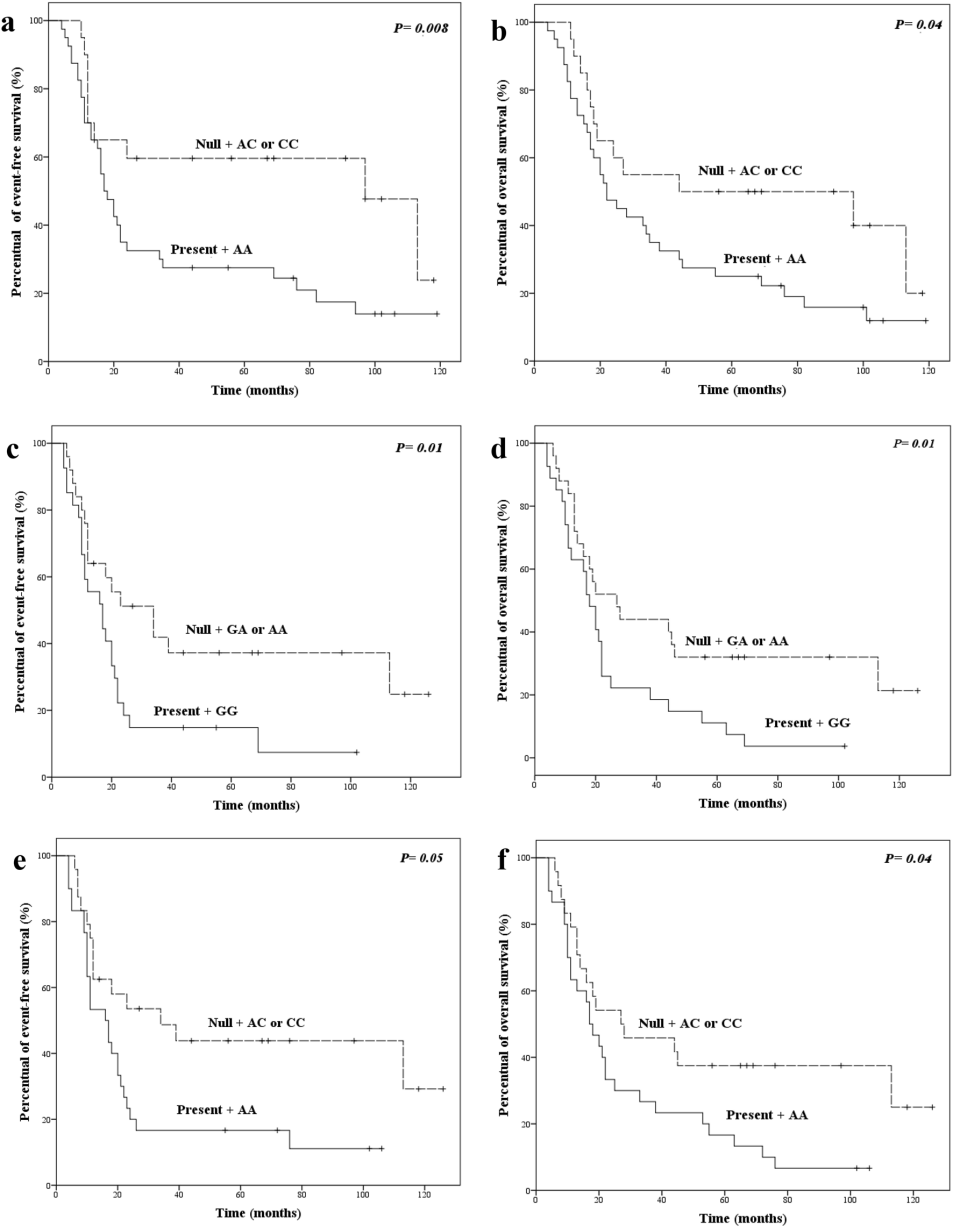
Event-free survival and overall survival in Kaplan Meier multivariate analysis of patients with *GSTM1* plus *XPC* c.2815A>C (**a**, **b**), *GSTM1* plus *XPD* c.934G>A (**c**, **d**), *GSTM1* plus *XPD* c.2251A>C (**e**, **f**) respectively.

EFS and OS of HNSCC patients with *GSTM1* plus *XPC* c.2815A>C, *GSTM1* plus *XPD* c.934G>A, *GSTM1* plus *XPD* c.225A>C, and *GSTM1* plus *TP53* c.215G>C are presented in Fig. 1.

## Discussion

The effects of CDDP and RT have been associated with genetic variability in distinct metabolic pathways^9–18^. Since patients may inherit defects in more than one pathway, we investigated in the current analysis of this prospective study the roles of eighteen SNVs involved in intracellular detoxification, DNA repair, and apoptosis pathways in the outcome of HNSCC patients treated with CDDP chemoradiation. We analyzed only combinations of two SNVs to obtain more consistent results.

We found that patients with large local tumors (T3 or T4) or tumors with large extensions to lymph nodes (N2 or N3) had 3.05 and 4.32 more chances of presenting PR or SD than others in multivariate Cox analysis. The worse response to therapy in these patients was expected in the study because when the tumor size advances, cells change the epithelial-mesenchymal transition^53,54^, having chemotherapy resistance due to abnormalities of cellular drug accumulation, DNA repair, and cytosolic drug inactivation as consequences^55^.

In the present multivariate Cox analysis, we observed that patients with XPC c.2815AC or CC had 3.43 times more chances of achieving PR or SD than others. We found an association between *XPD* c.934G>A and haplotype of *XPD* c.934G>A and c.2251A>C SNVs^15^ and *EXO1* c.1765G>A and haplotype of *EXO1* c.1765G>A and c.2270C>T SNVs^17^, but not of *XPC* c.2815A>C^15^, with response to CDDP chemoradiation in part of this cohort of patients (n = 88) in a previous analysis of NER or MMR pathways, respectively. It is possible the inclusion of new HNSCC patients (n = 21) in the current study may shed light on the real roles of the SNVs in response to CDDP chemoradiation. The association of *XPC* c.2815AC or CC with worse response to CDDP and RT was not expected in the study because the C allele of *XPC* c.2815A>C SNV was previously associated with lower DNA repair^22^, which seems to induce a better response to therapy. Nevertheless, Khan (2000)^56^ did not demonstrate a clear difference in the rate of nucleotide excision repair in the evaluation of the A and C alleles. Thus, further functional studies are needed to define the fundamental role of *XPC* c.2815A>C SNV in the DNA repair of CDDP lesions.

We found in multivariate Cox analysis that patients with advanced tumor size, T3 or T4, had 2.33 more chances of presenting tumor progression, relapse of tumor or death by effects of the disease, and 1.94 more chances of evolving to death than those with localized tumors in multivariate Cox analysis. These associations were also seen in other studies^57–59^, and again, these results may be attributed to changes in morphology and behavior of tumor cells during tumor growth^53,54^, favoring the dissemination of tumors^60,61^ and short survival^55^.

We observed in multivariate Cox analysis that patients with the *FAS* c.-671GG genotype had lower EFS and OS than those with the remaining genotypes and nearly two times more chances of presenting tumor progression, relapse of the tumor, or death than others. Our group previously published this result in the analysis of SNVs on genes of apoptosis pathways in the same cohort of patients^18^. The allele G was previously associated with reduced apoptosis of colorectal cancer cells because it affects the coupled binding of transcription factors SP1 and STAT1 to chromatin, altering complex recruitment for transcriptional activation^33^. It is also biologically plausible that the allele G attenuates transcriptional activation mediated by the SP1/STAT1 FAS complex in HNSCC, which in turn dampens the apoptotic pathway of FAS due to its dysregulated expression^32,62^, favoring the survival of tumor cells, tumor progression or relapse of tumor and death in patients with *FAS* c.-671GG genotype. Combinations of *FAS* c.-671A>G genotypes with other SNVs did not alter the EFS of HNSCC patients in the current analysis. The number of patients stratified by combined genotype may not have been sufficient to identify associations with patient survival.

Patients with *GSTM1* present plus *XPC* c.2815AA, *GSTM1* present plus *XPD* c.934GG (HR: 2.45 for EFS, HR: 2.37 for OS), *GSTM1* present plus *XPD* c.2251AA (HR: 1.93 for EFS, HR: 1.90 for OS), *GSTM1* present plus *TP53* c.215GC or CC (HR: 1.98 for EFS, HR: 1.93 for OS), and *XPD* c.2251AA plus *XPF* c.2505TT (HR: 2.0 for EFS, HR: 1.87 for OS) had more chances of tumor progression, relapse of tumor or death than others, but changes in survival of patients with distinct genotypes of isolated *GSTM1*, *XPD* c.934G>A, *XPD* c.2251A>C, *TP53* c.215G>C, and *XPF* c.2505T>C SNVs were not found in the current multivariate Cox analysis.

The presence of *GSTM1*^9^, *XPD* c.934GG^11,12^, and *XPD* c.2251AA^12^ genotypes were associated with shorter EFS in previous studies conducted by other groups. *XPD* c.934AA genotype was associated with lower EFS (HR: 2.12) and OS (HR: 2.04), but *XPF* c.2505T>C did not alter the survival of 90 HNSCC patients treated with CDDP and/or RT in a previous analysis of this prospective study, which focused on SNVs on genes of NER pathway^15^. It is worth commenting that previous and current analyses were based on a small number of patients with *XPD* c.934AA genotype (n = 10) and were adjusted by different variables. Isolated *TP53* c.215G>C SNV did not alter the survival of 109 HNSCC enrolled in previous^18^ and current analyses of this prospective study.

As far as our knowledge goes, there are no studies about associations of genotypes of different pathways of CDDP metabolism with the survival of HNSCC patients treated by CDDP chemoradiation. Associations of the above-mentioned combined genotypes with short survival were expected in the study. *GSTM1* present enhances CDDP detoxification of cells^4^ and the alleles A of *XPC* c.2815A>C^22^, G of *XPD* c.934G>A^23^, A of *XPD* c.2251A>C^23^, T of *XPF* c.2505T>C^24^, and C of *TP53* c.215G>C^29^ SNVs induce greater DNA repair and less apoptosis of damaged cells, respectively, favoring higher survival of tumor cells and lower survival of HNSCC patients. It is possible that the sum or synergism of functional abnormalities, such as detoxification and apoptosis, as seen in *GSTM1* present plus *TP53* c.215GC or CC combined genotype, and repair double defect, as seen in *XPD* c.2251AA plus *XPF* c.2505TT is necessary to alter the survival of HNSCC cells and HNSCC patients´ survival in the current analysis, and this the most plausible explanation for the association of combined genotypes but not of isolated genotypes with the survival of patients enrolled in the recent analysis of this prospective study.

It is worth commenting that patients’ survival was not substantially altered by isolated or combined genotypes of *GSTP1* c.313A>G, *EXO1* c.1765G>A, and *MSH3* c.3133G>A SNVs in the current analysis of this study, but *GSTP1* c.313GG genotype was associated with lower EFS in a previous analysis of the same cohort of patients (n = 90)^14^ and *EXO1* c.1765GG and *MSH3* c.3133GG genotypes were associated with lower EFS and OS, respectively, in a large sample of HNSCC (n = 397) analyzed previously by our group^16^. The number of patients and statistical adjustments in previous and current analyses may explain differences in results found by our group.

In summary, our data present isolated *XPC* c.2815A>C and *FAS* c.-671A>G SNVs, and for the first time, associations of *GSTM1* with *XPC* c.2815A>C, *XPD* c.934G>A, *XPD* c.2251A>C and *TP53* c.215G>C, and *XPD* c.2251A>C with *XPF* c.2505T>C SNVs, as independent factors for the outcome of HNSCC patients treated with CDD chemoradiation. We are aware that although a considerable number of patients were included in this complex and prospective pharmacogenetic study, we believe that a larger cohort of patients and additional functional analyses of *XPC* c.934G>A SNV in DNA repair may shed light on the roles of the SNVs in response and survival of HNSCC patients treated with CDDP chemoradiation. Thus, we believe that if our data is validated in further studies, specific SNVs on genes of CDDP metabolism can be used to select HNSCC with a high risk for unfavorable outcomes for a differentiated treatment.

### Supplementary Information

Supplementary Table S1.

Supplementary Table S2.

Supplementary Table S3.

Supplementary Table S4.

Supplementary Table S5.

## Data Availability

The datasets used and/or analyzed during the current study are available from the corresponding author upon reasonable request.
