# Supplementary material for: Genetic variability in cisplatin metabolic pathways and outcome of locally advanced head and neck squamous cell carcinoma patients
Source: Sci Rep. 2023 Oct 5;13:16762. doi: 10.1038/s41598-023-44040-7 (PMC10556039; doi:10.1038/s41598-023-44040-7)
Supplement: Supplementary file 1 — Supplementary Table S1. [file 41598_2023_44040_MOESM1_ESM.docx]

**Supplementary information 1**

Genetic variability in cisplatin metabolic pathways and outcome of locally advanced head and neck squamous cell carcinoma patients

Ana Maria Castro Ferreira^1^, João Maurício Carrasco Altemani^2^, Ligia Traldi Macedo^1^, Gustavo Jacob Lourenço^1^, Carmen Silvia Passos Lima^1,2^*

Corresponding author

^*^Carmen S. P. Lima, MD, PhD

Clinical Oncology Service

Department of Anesthesiology, Oncology and Radiology

Faculty of Medical Sciences

University of Campinas

Rua Alexander Fleming, 181

Cidade Universitária “Zeferino Vaz”

Barão Geraldo, Campinas, São Paulo, Brazil

CEP: 13083-970

Phone and fax simile: +55 19 3521 9120

E-mail: [carmenl@fcm.unicamp.br](mailto:carmenl@fcm.unicamp.br)

**Supplementary table S1**. PCR assays

| **Gene** | **Primers** | **Cycle conditions** |
| --- | --- | --- |
| ***GSTM1*** | F- CTGCCCTACTTGATTGATGGG  R- CTGGATTGTAGCAGATCATGC | 30 cycles:  1min at 95°C  1min at 62°C  1min at 72°C |
| ***GSTT1*** | F- TTCCTTACTGGTCCTCACATCTC  R- TCACCGGATCATGGCCAGCA | 30 cycles:  1min at 95°C  1min at 62°C  1min at 72°C |
| ***GSTP1*** | F- ACCCCAGGGCTCTATGGGAA  R- TGAGGGCACAAGAAGCCCCT | 35 cycles:  30s at 94°C  30s at 55°C  30s at 72°C  5min at 95°C  5min at 72°C |
| ***XPC* c.2815A>C** | F- ACCAGCTCTCAAGCAGAAGC  R- CTGCCTCAGTTTGCCTTCTC | 35 cycles:  30s at 95°C  40s at 63°C  40s at 72°C  5min at 95°C  10min at 72°C |
| ***XPD* c.934G>A** | F- GCCCGCTCTGGATTATACG  R- CTATCATCTCCTGGCCCCC | 38 cycles:  45s at 94°C  45s at 60°C  60s at 72°C  3min at 94°C  7min at 72°C |
| ***XPD* c.2251A>C** | F-CTGTTGGTGGGTGCCCGTATCTGTTGGTCT  R-TAATATCGGGGCTCACCCTGCAGCACTTCCT | 30 cycles:  30s at 94°C  30s at 60°C  60s at 72°C  4min at 94°C  5min at 72°C |
| ***XPF* c.2505T>C** | F- TCTCCATGTCCCGCTACTAC  R- GCAGGCACAGGCAAGTTCAA | 35 cycles:  1min at 94°C  1min at 67°C  1min at 72°C |
| ***ERCC1* c.354C>T** | F- TCATCCCTATTGATGGCTTCTGCCC  R- GACCATGCCCAGAGGCTTCTCATAG | 35 cycles:  1min at 94°C  1min at 69°C  1min at 72°C |
| ***MLH1* c.-93G>A** | F- CCGAGCTCCTAAAAACGAAC  R- CTGGCCGCTGGATAACTTC | 36 cycles:  30s at 95°C  30s at 58°C  30s at 72°C  5min at 95°C  10min at 72°C |
| ***MSH2* c.211+9C>G** | F- GACCGGGGCGACTTCTATAC  R -AAAGGAGCCGCGCCACAAGG | 35 cycles:  20s at 94ºC  20s at 54ºC  20s at 72ºC  5min at 95ºC  10min at 72ºC |
| ***MSH3* c.3133G>A** | F- TCACAGTCCTTGGTTATCTTGG  R- ACAAACTGGCGGATAATGGG | 35 cycles:  30s at 94ºC  30s at 55°C  30s at 72ºC  5min at 94°C  10min at 72°C |
| ***EXO1* c.1765G>A** | F- GACACAGATGTAGCACGTAA  R- CTGCGACACATCAGACATAT | 35 cycles:  30s at 94ºC  30s at 55°C  30s at 72ºC  5min at 94°C  10min at 72°C |
| ***TP53* c.215G > C** | F- ATCTACAGTCCCCCTTGCCG  R- GCAACTGACCGTGCAAGTCA | 35 cycles:  40s at 94°C  30s at 56°C  30s at 72°C  4min at 94°C |
| ***CASP3* c.-1191A > G** | F- TGTGTATCCGT GGCCACAG CT  R- GAGAATGGGGGAAGAGGCAGGT | 35 cycles:  45s at 96°C  40s at 56°C  30s at 72°C  5min at 96°C  10min at 72°C |
| ***CASP3* c.-182-247G > T** | F- GCGGTAGCGCCGTCCGTTGC  R- ACCGAGCTCCGAGGGCGGGAG | 35 cycles:  45s at 96°C  40s at 56°C  30s at 72°C  5min at 96°C  10min at 72°C |
| ***FAS* c.-671A > G** | F- CTACCTAAGAGCTATCTACCGTTC  R- GGCTGTCCATGTTGTGGCTGC | 28 cycles:  30s at 94ºC  30s at 62ºC  1min at 72ºC  6min at 94ºC  10min at 72ºC |
| ***FAS* c.-1378G > A** | F- TGTGTGCACAAGGCTGGCGC  R- TGCATCTGTCACTGC ACTTACCACCA | 35 cycles:  30s at 94˚C  30s at 62˚C  45s at 72˚C  2min at 94˚C  7min at 72˚C |
| ***FASL* c.-844 C > T** | F- CAGCTACTCGGAGGCCAAG  R- GCTCTGAGGGGAGAGACCAT | 35 cycles:  30s at 94˚C  30s at 62˚C  45s at 72˚C  2min at 94˚C  7min at 72˚C |

F: forward sequence; R: reverse sequence; s: seconds; min: minutes
