# Supplementary material for: Genetic variability in cisplatin metabolic pathways and outcome of locally advanced head and neck squamous cell carcinoma patients
Source: Sci Rep. 2023 Oct 5;13:16762. doi: 10.1038/s41598-023-44040-7 (PMC10556039; doi:10.1038/s41598-023-44040-7)
Supplement: Supplementary file 2 — Supplementary Table S2. [file 41598_2023_44040_MOESM2_ESM.docx]

**Supplementary information 2**

Genetic variability in cisplatin metabolic pathways and outcome of locally advanced head and neck squamous cell carcinoma patients

Ana Maria Castro Ferreira^1^, João Maurício Carrasco Altemani^2^, Ligia Traldi Macedo^1^, Gustavo Jacob Lourenço^1^, Carmen Silvia Passos Lima^1,2^*

Corresponding author

^*^Carmen S. P. Lima, MD, PhD

Clinical Oncology Service

Department of Anesthesiology, Oncology and Radiology

Faculty of Medical Sciences

University of Campinas

Rua Alexander Fleming, 181

Cidade Universitária “Zeferino Vaz”

Barão Geraldo, Campinas, São Paulo, Brazil

CEP: 13083-970

Phone and fax simile: +55 19 3521 9120

E-mail: [carmenl@fcm.unicamp.br](mailto:carmenl@fcm.unicamp.br)

**Supplementary table S2**. Combination in pairs according to the role of the allele in cisplatin metabolism

| **Gene** | **Allele** | **Paired gene** | **Allele** | **Function** |
| --- | --- | --- | --- | --- |
| *GSTM1* | *Present* | *GSTT1* | *Present* | ND + ND |
|  |  | *GSTP1* c.313A>G | A | ND + ND |
|  |  | *XPC* c.2815A>C | A | ND + NR |
|  |  | *XPD* c.934G>A | G | ND + NR |
|  |  | *XPD* c.2251A>C | A | ND + NR |
|  |  | *XPF* c.2505T>C | T | ND + NR |
|  |  | *ERCC1* c.354C>T | C | ND + NR |
|  |  | *MLH1* c.93G>A | G | ND + NR |
|  |  | *MSH2* c.211+9C>G | C | ND + NR |
|  |  | *MSH3 c*.3133G>A | G | ND + NR |
|  |  | *EXO1* c.1762G>A | G | ND + NR |
|  |  | *TP53* c.215G>C | C | ND + RA |
|  |  | *CASP3* c.-1191A>G | G | ND + RA |
|  |  | *CASP3* c.-182-247G>T | T | ND + RA |
|  |  | *FAS* c.-1378G>A | A | ND + RA |
|  |  | *FAS* c.-671A>G | G | ND + RA |
|  |  | *FASL* c.-844C>T | T | ND + RA |
|  |  |  |  |  |
| *GSTT1* | *Present* | *GSTP1* c.313A>G | A | ND + ND |
|  |  | *XPC* c.2815A>C | A | ND + NR |
|  |  | *XPD* c.934G>A | G | ND + NR |
|  |  | *XPD* c.2251A>C | A | ND + NR |
|  |  | *XPF* c.2505T>C | T | ND + NR |
|  |  | *ERCC1* c.354C>T | C | ND + NR |
|  |  | *MLH1* c.93G>A | G | ND + NR |
|  |  | *MSH2* c.211+9C>G | C | ND + NR |
|  |  | *MSH3 c*.3133G>A | G | ND + NR |
|  |  | *EXO1* c.1762G>A | G | ND + NR |
|  |  | *TP53* c.215G>C | C | ND + RA |
|  |  | *CASP3* c.-1191A>G | G | ND + RA |
|  |  | *CASP3* c.-182-247G>T | T | ND + RA |
|  |  | *FAS* c.-1378G>A | A | ND + RA |
|  |  | *FAS* c.-671A>G | G | ND + RA |
|  |  | *FASL* c.-844C>T | T | ND + RA |
|  |  |  |  |  |
| *GSTP1* c.313A>G | A | *XPC* c.2815A>C | A | ND + NR |
|  |  | *XPD* c.934G>A | G | ND + NR |
|  |  | *XPD* c.2251A>C | A | ND + NR |
|  |  | *XPF* c.2505T>C | T | ND + NR |
|  |  | *ERCC1* c.354C>T | C | ND + NR |
|  |  | *MLH1* c.93G>A | G | ND + NR |
|  |  | *MSH2* c.211+9C>G | C | ND + NR |
|  |  | *MSH3 c*.3133G>A | G | ND + NR |
|  |  | *EXO1* c.1762G>A | G | ND + NR |
|  |  | *TP53* c.215G>C | C | ND + RA |
|  |  | *CASP3* c.-1191A>G | G | ND + RA |
|  |  | *CASP3* c.-182-247G>T | T | ND + RA |
|  |  | *FAS* c.-1378G>A | A | ND + RA |
|  |  | *FAS* c.-671A>G | G | ND + RA |
|  |  | *FASL* c.-844C>T | T | ND + RA |
|  |  |  |  |  |
| *XPC* c.2815A>C | A | *XPD* c.934G>A | G | NR + NR |
|  |  | *XPD* c.2251A>C | A | NR + NR |
|  |  | *XPF* c.2505T>C | T | NR + NR |
|  |  | *ERCC1* c.354C>T | C | NR + NR |
|  |  | *MLH1* c.93G>A | G | NR + NR |
|  |  | *MSH2* c.211+9C>G | C | NR + NR |
|  |  | *MSH3 c*.3133G>A | G | NR + NR |
|  |  | *EXO1* c.1762G>A | G | NR + NR |
|  |  | *TP53* c.215G>C | C | NR + RA |
|  |  | *CASP3* c.-1191A>G | G | NR + RA |
|  |  | *CASP3* c.-182-247G>T | T | NR + RA |
|  |  | *FAS* c.-1378G>A | A | NR + RA |
|  |  | *FAS* c.-671A>G | G | NR + RA |
|  |  | *FASL* c.-844C>T | T | NR + RA |
|  |  |  |  |  |
| *XPD* c.934G>A | G | *XPD* c.2251A>C | A | NR + NR |
|  |  | *XPF* c.2505T>C | T | NR + NR |
|  |  | *ERCC1* c.354C>T | C | NR + NR |
|  |  | *MLH1* c.93G>A | G | NR + NR |
|  |  | *MSH2* c.211+9C>G | C | NR + NR |
|  |  | *MSH3 c*.3133G>A | G | NR + NR |
|  |  | *EXO1* c.1762G>A | G | NR + NR |
|  |  | *TP53* c.215G>C | C | NR + RA |
|  |  | *CASP3* c.-1191A>G | G | NR + RA |
|  |  | *CASP3* c.-182-247G>T | T | NR + RA |
|  |  | *FAS* c.-1378G>A | A | NR + RA |
|  |  | *FAS* c.-671A>G | G | NR + RA |
|  |  | *FASL* c.-844C>T | T | NR + RA |
|  |  |  |  |  |
| *XPD* c.2251A>C | A | *XPF* c.2505T>C | T | NR + NR |
|  |  | *ERCC1* c.354C>T | C | NR + NR |
|  |  | *MLH1* c.93G>A | G | NR + NR |
|  |  | *MSH2* c.211+9C>G | C | NR + NR |
|  |  | *MSH3 c*.3133G>A | G | NR + NR |
|  |  | *EXO1* c.1762G>A | G | NR + NR |
|  |  | *TP53* c.215G>C | C | NR + RA |
|  |  | *CASP3* c.-1191A>G | G | NR + RA |
|  |  | *CASP3* c.-182-247G>T | T | NR + RA |
|  |  | *FAS* c.-1378G>A | A | NR + RA |
|  |  | *FAS* c.-671A>G | G | NR + RA |
|  |  | *FASL* c.-844C>T | T | NR + RA |
|  |  |  |  |  |
| *XPF* c.2505T>C | T | *ERCC1* c.354C>T | C | NR + NR |
|  |  | *MLH1* c.93G>A | G | NR + NR |
|  |  | *MSH2* c.211+9C>G | C | NR + NR |
|  |  | *MSH3 c*.3133G>A | G | NR + NR |
|  |  | *EXO1* c.1762G>A | G | NR + NR |
|  |  | *TP53* c.215G>C | C | NR + RA |
|  |  | *CASP3* c.-1191A>G | G | NR + RA |
|  |  | *CASP3* c.-182-247G>T | T | NR + RA |
|  |  | *FAS* c.-1378G>A | A | NR + RA |
|  |  | *FAS* c.-671A>G | G | NR + RA |
|  |  | *FASL* c.-844C>T | T | NR + RA |
|  |  |  |  |  |
| *ERCC1* c.354C>T | C | *MLH1* c.93G>A | G | NR + NR |
|  |  | *MSH2* c.211+9C>G | C | NR + NR |
|  |  | *MSH3 c*.3133G>A | G | NR + NR |
|  |  | *EXO1* c.1762G>A | G | NR + NR |
|  |  | *TP53* c.215G>C | C | NR + RA |
|  |  | *CASP3* c.-1191A>G | G | NR + RA |
|  |  | *CASP3* c.-182-247G>T | T | NR + RA |
|  |  | *FAS* c.-1378G>A | A | NR + RA |
|  |  | *FAS* c.-671A>G | G | NR + RA |
|  |  | *FASL* c.-844C>T | T | NR + RA |
| *MLH1* c.93G>A | G | *MSH2* c.211+9C>G | C | NR + NR |
|  |  | *MSH3 c*.3133G>A | G | NR + NR |
|  |  | *EXO1* c.1762G>A | G | NR + NR |
|  |  | *TP53* c.215G>C | C | NR + RA |
|  |  | *CASP3* c.-1191A>G | G | NR + RA |
|  |  | *CASP3* c.-182-247G>T | T | NR + RA |
|  |  | *FAS* c.-1378G>A | A | NR + RA |
|  |  | *FAS* c.-671A>G | G | NR + RA |
|  |  | *FASL* c.-844C>T | T | NR + RA |
|  |  |  |  |  |
| *MSH2* c.211+9C>G | C | *MSH3 c*.3133G>A | G | NR + NR |
|  |  | *EXO1* c.1762G>A | G | NR + NR |
|  |  | *TP53* c.215G>C | C | NR + RA |
|  |  | *CASP3* c.-1191A>G | G | NR + RA |
|  |  | *CASP3* c.-182-247G>T | T | NR + RA |
|  |  | *FAS* c.-1378G>A | A | NR + RA |
|  |  | *FAS* c.-671A>G | G | NR + RA |
|  |  | *FASL* c.-844C>T | T | NR + RA |
|  |  |  |  |  |
| *MSH3 c*.3133G>A | G | *EXO1* c.1762G>A | G | NR + NR |
|  |  | *TP53* c.215G>C | C | NR + RA |
|  |  | *CASP3* c.-1191A>G | G | NR + RA |
|  |  | *CASP3* c.-182-247G>T | T | NR + RA |
|  |  | *FAS* c.-1378G>A | A | NR + RA |
|  |  | *FAS* c.-671A>G | G | NR + RA |
|  |  | *FASL* c.-844C>T | T | NR + RA |
|  |  |  |  |  |
| *EXO1* c.1762G>A | G | *TP53* c.215G>C | C | NR + RA |
|  |  | *CASP3* c.-1191A>G | G | NR + RA |
|  |  | *CASP3* c.-182-247G>T | T | NR + RA |
|  |  | *FAS* c.-1378G>A | A | NR + RA |
|  |  | *FAS* c.-671A>G | G | NR + RA |
|  |  | *FASL* c.-844C>T | T | NR + RA |
|  |  |  |  |  |
| *TP53* c.215G>C | C | *CASP3* c.-1191A>G | G | RA + RA |
|  |  | *CASP3* c.-182-247G>T | T | RA + RA |
|  |  | *FAS* c.-1378G>A | A | RA + RA |
|  |  | *FAS* c.-671A>G | G | RA + RA |
|  |  | *FASL* c.-844C>T | T | RA + RA |
|  |  |  |  |  |
| *CASP3* c.-1191A>G | G | *CASP3* c.-182-247G>T | T | RA + RA |
|  |  | *FAS* c.-1378G>A | A | RA + RA |
|  |  | *FAS* c.-671A>G | G | RA + RA |
|  |  | *FASL* c.-844C>T | T | RA + RA |
|  |  |  |  |  |
| *CASP3* c.-182-247G>T | T | *FAS* c.-1378G>A | A | RA + RA |
|  |  | *FAS* c.-671A>G | G | RA + RA |
|  |  | *FASL* c.-844C>T | T | RA + RA |
|  |  |  |  |  |
| *FAS* c.-1378G>A | A | *FAS* c.-671A>G | G | RA + RA |
|  |  | *FASL* c.-844C>T | T | RA + RA |
|  |  |  |  |  |
| *FAS* c.-671A>G | G | *FASL* c.-844C>T | T | RA + RA |

ND: normal detoxification; NR: normal repair; RA: reduced apoptosis
