# Supplementary material for: Genetic variability in cisplatin metabolic pathways and outcome of locally advanced head and neck squamous cell carcinoma patients
Source: Sci Rep. 2023 Oct 5;13:16762. doi: 10.1038/s41598-023-44040-7 (PMC10556039; doi:10.1038/s41598-023-44040-7)
Supplement: Supplementary file 3 — Supplementary Table S3. [file 41598_2023_44040_MOESM3_ESM.docx]

**Supplementary information 3**

Genetic variability in cisplatin metabolic pathways and outcome of locally advanced head and neck squamous cell carcinoma patients

Ana Maria Castro Ferreira^1^, João Maurício Carrasco Altemani^2^, Ligia Traldi Macedo^1^, Gustavo Jacob Lourenço^1^, Carmen Silvia Passos Lima^1,2^*

Corresponding author

^*^Carmen S. P. Lima, MD, PhD

Clinical Oncology Service

Department of Anesthesiology, Oncology and Radiology

Faculty of Medical Sciences

University of Campinas

Rua Alexander Fleming, 181

Cidade Universitária “Zeferino Vaz”

Barão Geraldo, Campinas, São Paulo, Brazil

CEP: 13083-970

Phone and fax simile: +55 19 3521 9120

E-mail: [carmenl@fcm.unicamp.br](mailto:carmenl@fcm.unicamp.br)

**Supplementary table S3**. Clinicopathological aspects of 88 patients with head and neck squamous cell carcinoma in response rate to cisplatin chemoradiotherapy

| **Variable** | **N** | **Response rate** | | | |
| --- | --- | --- | --- | --- | --- |
|  |  | **CR** N (%) or mean (SD)** | **PR or SD** N (%) or mean (SD)** | **Univariate analysis** | |
|  |  |  |  | **OR (95% CI)** | ***P* value** |
| **Median age** |  |  |  |  |  |
| ≤ 56 years | 46 | 7 (33.3) | 39 (58.2) | 2.78 (0.99-7.79) | 0.06 |
| > 56 years | 42 | 14 (66.7) | 28 (41.8) | Reference |  |
| **Gender** |  |  |  |  |  |
| Male | 82 | 19 (90.5) | 63 (94.0) | 1.65 (0.28-9.76) | 0.57 |
| Female | 6 | 2 (9.5) | 4 (6.0) | Reference |  |
| **Body mass index (kg/m^2^)** | 88 | 20.4 (4.0) | 19.4 (4.16) | 1.06 (0.94-1.19) | 0.31 |
| **Tumor location** |  |  |  |  |  |
| Oral cavity or pharynx | 22 | 6 (28.6) | 16 (23.9) | Reference | 0.66 |
| Larynx | 66 | 15 (71.4) | 51 (76.1) | 1.27 (0.42-3.83) |  |
| **Histological grade^*^** |  |  |  |  |  |
| Well or moderately | 59 | 12 (75.0) | 47 (85.5) | 1.95 (0.50-7.61) | 0.33 |
| Poorly or undifferentiated | 12 | 4 (25.0) | 8 (14.5) | Reference |  |
| **Tumor size** |  |  |  |  |  |
| T1 or T2 | 23 | 9 (42.9) | 14 (20.9) | Reference | **0.05** |
| T3 or T4 | 65 | 12 (57.1) | 53 (79.1) | **2.83 (0.99-8.08)** |  |
| **Nodal status** |  |  |  |  |  |
| N0 or N1 | 30 | 13 (61.9) | 17 (25.4) | Reference | **0.003** |
| N2 or N3 | 58 | 8 (38.1) | 50 (74.6) | **4.77 (1.69-13.50)** |  |
| **Tumor stage** |  |  |  |  |  |
| I or II | 4 | 2 (9.5) | 2 (3.0) | Reference | 0.23 |
| III or IV | 84 | 19 (90.5) | 65 (97.0) | 3.42 (0.45-25.93) |  |
| **Dose of CDDP (mg/m^2^)** | 88 | 244.5 (43.8) | 253.8 (45.4) | 0.99 (0.98-1.00) | 0.41 |

N: number of patients; CR: complete response; PR: partial response; SD: stable disease; SD: standard deviation; OR: odds ratio; CI: confidence interval. ^*^The numbers of patients differed from the total quoted in the study because it was not possible to obtain available tumor fragments and consistent data of response to chemoradiation in some cases, respectively. Results with significant *P-*values are presented in bold letters. Variables with *P* values < 0.20 were included in the multivariate analysis
