# Supplementary material for: Genetic variability in cisplatin metabolic pathways and outcome of locally advanced head and neck squamous cell carcinoma patients
Source: Sci Rep. 2023 Oct 5;13:16762. doi: 10.1038/s41598-023-44040-7 (PMC10556039; doi:10.1038/s41598-023-44040-7)
Supplement: Supplementary file 4 — Supplementary Table S4. [file 41598_2023_44040_MOESM4_ESM.docx]

**Supplementary information 4**

Genetic variability in cisplatin metabolic pathways and outcome of locally advanced head and neck squamous cell carcinoma patients

Ana Maria Castro Ferreira^1^, João Maurício Carrasco Altemani^2^, Ligia Traldi Macedo^1^, Gustavo Jacob Lourenço^1^, Carmen Silvia Passos Lima^1,2^*

Corresponding author

^*^Carmen S. P. Lima, MD, PhD

Clinical Oncology Service

Department of Anesthesiology, Oncology and Radiology

Faculty of Medical Sciences

University of Campinas

Rua Alexander Fleming, 181

Cidade Universitária “Zeferino Vaz”

Barão Geraldo, Campinas, São Paulo, Brazil

CEP: 13083-970

Phone and fax simile: +55 19 3521 9120

E-mail: [carmenl@fcm.unicamp.br](mailto:carmenl@fcm.unicamp.br)

**Supplementary table S4**. Genotypes of detoxification, DNA-repair, and apoptosis-related single nucleotide variants of 88 patients with head and neck squamous cell carcinoma in response rate to chemoradiotherapy

| **Genotype** | **N** | **Response rate** | | | |
| --- | --- | --- | --- | --- | --- |
|  |  | **CR N (%)** | **PR or SD N (%)** | **Univariate analysis** | |
|  |  |  |  | **OR (95% CI)** | ***P* value** |
| ***GSTM1*** |  |  |  |  |  |
| Present | 38 | 7 (33.3) | 31 (46.3) | 1.72 (0.61-4.80) | 0.29 |
| Null | 50 | 14 (66.7) | 36 (53.7) | Reference |  |
| ***GSTT1*** |  |  |  |  |  |
| Present | 73 | 16 (76.2) | 57 (85.1) | 1.78 (0.53-5.96) | 0.34 |
| Null | 15 | 5 (23.8) | 10 (14.9) | Reference |  |
| ***GSTP1* c.313A>G** |  |  |  |  |  |
| AA | 41 | 10 (47.6) | 31 (46.3) | Reference | 0.91 |
| AG or GG | 47 | 11 (52.4) | 36 (53.7) | 1.05 (0.39-2.81) |  |
| AA or AG | 83 | 20 (95.2) | 63 (94.0) | Reference | 0.83 |
| GG | 5 | 1 (4.8) | 4 (6.0) | 1.27 (0.13-12.02) |  |
| ***XPC* c.2815A>C** |  |  |  |  |  |
| AA | 31 | 10 (47.6) | 21 (31.3) | Reference | 0.17 |
| AC or CC | 57 | 11 (52.4) | 46 (68.7) | 1.99 (0.73-5.41) |  |
| AA or AC | 76 | 18 (85.7) | 58 (86.6) | 1.07 (0.26-4.39) | 0.92 |
| CC | 12 | 3 (14.3) | 9 (13.4) | Reference |  |
| ***XPD* c.934G>A** |  |  |  |  |  |
| GG | 47 | 11 (52.4) | 36 (53.7) | 1.05 (0.39-2.81) | 0.91 |
| GA or AA | 41 | 10 (47.6) | 31 (46.3) | Reference |  |
| GG or GA | 80 | 18 (85.7) | 62 (92.5) | 2.06 (0.45-9.49) | 0.35 |
| AA | 8 | 3 (14.3) | 5 (7.5) | Reference |  |
| ***XPD* c.2251A>C** |  |  |  |  |  |
| AA | 44 | 11 (52.4) | 33 (49.3) | Reference | 0.80 |
| AC or CC | 44 | 10 (47.6) | 34 (50.7) | 1.13 (0.42-3.02) |  |
| AA or AC | 79 | 19 (90.5) | 60 (89.6) | Reference | 0.90 |
| CC | 9 | 2 (9.5) | 7 (10.4) | 1.10 (0.21-5.79) |  |
| ***XPF* c.2505T>C** |  |  |  |  |  |
| TT | 37 | 10 (47.6) | 27 (40.3) | Reference | 0.55 |
| TC or CC | 51 | 11 (52.4) | 40 (59.7) | 1.34 (0.50-3.60) |  |
| TT or TC | 80 | 20 (95.2) | 60 (89.6) | Reference | 0.44 |
| CC | 8 | 1 (4.8) | 7 (10.4) | 2.33 (0.27-20.14) |  |
| ***ERCC1* c.354C>T** |  |  |  |  |  |
| CC | 24 | 7 (33.3) | 17 (25.4) | Reference | 0.47 |
| CT or TT | 64 | 14 (66.7) | 50 (74.6) | 1.47 (0.50-4.24) |  |
| CC or CT | 72 | 17 (81.0) | 55 (82.1) | 1.07 (0.30-3.78) | 0.90 |
| TT | 16 | 4 (19.0) | 12 (17.9) | Reference |  |
| ***MLH1* c.93G>A** |  |  |  |  |  |
| GG | 52 | 13 (61.9) | 39 (58.2) | Reference | 0.76 |
| GA or AA | 36 | 8 (38.1) | 28 (41.8) | 1.16 (0.42-3.18) |  |
| GG or GA | 84 | 19 (90.5) | 65 (97.0) | 3.42 (0.45-25.93) | 0.23 |
| AA | 4 | 2 (0.5) | 2 (3.0) | Reference |  |
| ***MSH2* c.211+9C>G** |  |  |  |  |  |
| CC | 19 | 7 (33.3) | 12 (17.9) | Reference | 0.14 |
| CG or GG | 69 | 14 (66.7) | 55 (82.1) | 2.29 (0.76-6.89) |  |
| CC or CG | 67 | 15 (71.4) | 52 (77.6) | 1.38 (0.45-4.19) | 0.56 |
| GG | 21 | 6 (28.6) | 15 (22.4) | Reference |  |
| ***MSH3* c.3133G>A** |  |  |  |  |  |
| GG | 6 | 1 (4.8) | 5 (7.5) | 1.61 (0.17-14.63) | 0.67 |
| GA or AA | 82 | 20 (95.2) | 62 (92.5) | Reference |  |
| GG or GA | 37 | 9 (42.9) | 28 (41.8) | Reference | 0.93 |
| AA | 51 | 12 (57.1) | 39 (58.2) | 1.04 (0.38-2.81) |  |
| ***EXO1* c.1765G>A** |  |  |  |  |  |
| GG | 37 | 11 (52.4) | 26 (38.8) | 2.73 (0.64-4.65) | 0.27 |
| GA or AA | 51 | 10 (47.6) | 41 (61.2) | Reference |  |
| GG or GA | 77 | 18 (85.7) | 59 (88.1) | 1.22 (0.29-5.12) | 0.77 |
| AA | 11 | 3 (14.3) | 8 (11.9) | Reference |  |
| ***TP53* c.215G>C** |  |  |  |  |  |
| CC | 9 | 1 (4.8) | 8 (11.9) | 2.71 (0.31-23.04) | 0.36 |
| GC or GG | 79 | 20 (95.2) | 59 (88.1) | Reference |  |
| CC or GC | 49 | 12 (57.1) | 37 (55.2) | 1.08 (0.40-2.90) | 0.87 |
| GG | 39 | 9 (42.9) | 30 (44.8) | Reference |  |
| ***CASP3* c.-1191A>G** |  |  |  |  |  |
| GG | 9 | 2 (9.5) | 7 (10.4) | 1.10 (0.21-5.79) | 0.90 |
| GA or AA | 79 | 19 (90.5) | 60 (89.6) | Reference |  |
| GG or GA | 36 | 13 (61.9) | 39 (58.2) | Reference | 0.76 |
| AA | 52 | 8 (38.1) | 28 (41.8) | 1.16 (0.42-3.18) |  |
| ***CASP3* c.-182-247G>T** |  |  |  |  |  |
| TT | 76 | 18 (85.7) | 58 (87.9) | 1.47 (0.29-7.43) | 0.63 |
| GT or GG | 11 | 3 (14.3) | 8 (12.1) | Reference |  |
| TT or GT | 29 | 9 (42.9) | 20 (30.6) | Reference | 0.74 |
| GG | 58 | 12 (57.1) | 46 (69.7) | 1.18 (0.43-3.19) |  |
| ***FAS* c.-1378G>A** |  |  |  |  |  |
| AA | 6 | 3 (14.3) | 3 (4.5) | Reference | 0.14 |
| GA or GG | 82 | 18 (85.7) | 64 (95.5) | 3.55 (0.66-19.14) |  |
| AA or GA | 27 | 10 (47.6) | 17 (25.4) | **Reference** | **0.05** |
| GG | 61 | 11 (52.4) | 50 (74.6) | **2.67 (0.96-7.40)** |  |
| ***FAS* c.-671A>G** |  |  |  |  |  |
| GG | 21 | 5 (23.8) | 16 (23.9) | 1.00 (0.31-3.17) | 0.99 |
| AG or AA | 67 | 16 (76.2) | 51 (76.1) | Reference |  |
| GG or AG | 62 | 14 (66.7) | 48 (71.6) | 1.26 (0.44-3.61) | 0.66 |
| AA | 26 | 7 (33.3) | 19 (28.4) | Reference |  |
| ***FASL* c.-844C>T** |  |  |  |  |  |
| TT | 22 | 5 (23.8) | 17 (25.4) | 1.08 (0.34-3.41) | 0.88 |
| CC or CT | 66 | 16 (76.2) | 50 (74.6) | Reference |  |
| TT or CT | 60 | 17 (81.0) | 43 (64.2) | Reference | 0.15 |
| CC | 28 | 4 (19.0) | 24 (35.8) | 2.37 (0.71-7.86) |  |
| ***GSTM1 + GSTT1*** |  |  |  |  |  |
| Present + Present | 8 | 3 (37.5) | 5 (16.1) | Reference | 0.19 |
| Null + Null | 31 | 5 (62.5) | 26 (83.9) | 3.12 (0.55-17.45) |  |
| ***GSTM1 + GSTP1* c.313A>G** |  |  |  |  |  |
| Present + AA | 26 | 9 (64.3) | 17 (58.6) | Reference | 0.72 |
| Null + AG or GG | 17 | 5 (37.5) | 12 (41.4) | 1.27 (0.34-4.75) |  |
| Present + AA or AG | 2 | 0 (0.0) | 2 (6.5) | NE | NE |
| Null + GG | 35 | 6 (100.0) | 29 (93.5) |  |  |
| ***GSTM1 + XPC* c.2815A>C** |  |  |  |  |  |
| Present + AA | 35 | 7 (70.0) | 28 (68.3) | Reference | 0.91 |
| Null + AC or CC | 16 | 3 (30.0) | 13 (31.7) | 1.08 (0.24-4.87) |  |
| Present + AA or AC | 6 | 2 (25.0) | 4 (13.3) | Reference | 0.42 |
| Null + CC | 32 | 6 (75.0) | 26 (86.7) | 2.16 (0.31-14.71) |  |
| ***GSTM1 + XPD* c.934G>A** |  |  |  |  |  |
| Present + GG | 23 | 7 (63.6) | 16 (50.0) | Reference | 0.43 |
| Null + GA or AA | 20 | 4 (36.4) | 16 (50.0) | 1.75 (0.42-7.17) |  |
| Present + GG or GA | 5 | 3 (30.0) | 2 (6.7) | Reference | 0.07 |
| Null + AA | 35 | 7 (70.0) | 28 (93.3) | 6.00 (0.71-7.86) |  |
| ***GSTM1 + XPD* c.2251A>C** |  |  |  |  |  |
| Present + AA | 25 | 7 (63.6) | 18 (54.5) | Reference | 0.59 |
| Null + AC or CC | 19 | 4 (36.4) | 15 (45.5) | 1.45 (0.35-5.95) |  |
| Present + AA or AC | 4 | 2 (22.2) | 2 (7.1) | Reference | 0.22 |
| Null + CC | 33 | 7 (77.8) | 26 (92.9) | 3.71 (0.44-31.26) |  |
| ***GSTM1 +XPF* c.2505T>C** |  |  |  |  |  |
| Present + TT | 28 | 6 (75.0) | 22 (62.9) | Reference | 0.51 |
| Null + TC or CC | 15 | 2 (25.0) | 13 (37.1) | 1.77 (0.31-10.11) |  |
| Present + TT or TC | 4 | 0 (0.0) | 4 (12.5) | NE | NE |
| Null + CC | 34 | 6 (100.0) | 28 (87.5) |  |  |
| ***GSTM1 + ERCC1* c.354C>T** |  |  |  |  |  |
| Present + CC | 38 | 8 (88.9) | 30 (73.2) | Reference | 0.33 |
| Null + CT or TT | 12 | 1 (11.1) | 11 (26.8) | 2.93 (0.32-26.22) |  |
| Present + CC or CT | 10 | 3 (33.3) | 7 (21.2) | Reference | 0.45 |
| Null + TT | 32 | 6 (66.7) | 26 (78.8) | 1.85 (0.36-9.36) |  |
| ***GSTM1 + MLH1* c.93G>A** |  |  |  |  |  |
| Present + GG | 23 | 5 (55.6) | 18 (46.2) | Reference | 0.61 |
| Null + GA or AA | 25 | 4 (44.4) | 21 (53.8) | 1.45 (0.33-6.26) |  |
| Present + GG or GA | 3 | 1 (14.3) | 2 (6.1) | Reference | 0.46 |
| Null + AA | 37 | 6 (85.7) | 31 (93.9) | 2.58 (0.20-33.24) |  |
| ***GSTM1 + MSH2* c.211+9C>G** |  |  |  |  |  |
| Present + CC | 41 | 10 (76.9) | 31 (81.6) | 1.32 (0.28-6.12) | 0.71 |
| Null + CG or GG | 10 | 3 (23.1) | 7 (18.4) | Reference |  |
| Present + CC or CG | 12 | 6 (46.2) | 6 (21.4) | Reference | 0.11 |
| Null + GG | 29 | 7 (53.8) | 22 (78.6) | 3.14 (0.76-12.94) |  |
| ***GSTM1 + MSH3* c.3133G>A** |  |  |  |  |  |
| Present + GG | 46 | 14 (93.3) | 32 (97.0) | 2.28 (0.13-39.20) | 0.56 |
| Null + GA or AA | 2 | 1 (6.7) | 1 (3.0) | Reference |  |
| Present + GG or GA | 28 | 8 (72.7) | 20 (62.5) | Reference | 0.54 |
| Null + AA | 15 | 3 (27.3) | 12 (37.5) | 1.60 (0.35-7.22) |  |
| ***GSTM1 + EXO1* c.1765G>A** |  |  |  |  |  |
| Present + GG | 28 | 5 (71.4) | 23 (36.1) | Reference | 0.70 |
| Null + GA or AA | 15 | 2 (28.6) | 12 (36.1) | 1.41 (0.23-8.33) |  |
| Present + GG or GA | 5 | 1 (16.7) | 4 (12.9) | Reference | 0.80 |
| Null + AA | 32 | 5 (83.3) | 27 (87.1) | 1.35 (0.12-14.73) |  |
| ***GSTM1 + TP53* c.215G>C** |  |  |  |  |  |
| Present + CC | 4 | 0 (0.0) | 4 (12.5) | NE | NE |
| Null + GC or GG | 34 | 6 (100.0) | 28 (87.5) |  |  |
| Present + CC or GC | 23 | 6 (60.0) | 17 (48.6) | Reference | 0.52 |
| Null + GG | 22 | 4 (40.0) | 18 (51.4) | 1.58 (0.38-6.62) |  |
| ***GSTM1 + CASP3* c.-1191A>G** |  |  |  |  |  |
| Present + GG | 45 | 13 (92.9) | 32 (91.4) | Reference | 0.86 |
| Null + GA or AA | 4 | 1 (7.1) | 3 (8.6) | 1.21 (0.11-12.82) |  |
| Present + GG or GA | 19 | 5 (55.6) | 14 (45.2) | Reference | 0.58 |
| Null + AA | 21 | 4 (44.4) | 17 (54.8) | 1.51 (0.34-6.75) |  |
| ***GSTM1 + CASP3* c.-182-247G>T** |  |  |  |  |  |
| Present + TT | 41 | 12 (100.0) | 29 (93.5) | NE | NE |
| Null + TT or GT | 2 | 0 (0.0) | 2 (6.5) |  |  |
| Present + TT or GT | 19 | 5 (62.5) | 14 (42.4) | Reference | 0.31 |
| Null + GG | 23 | 3 (37.5) | 19 (57.6) | 2.26 (0.46-11.08) |  |
| ***GSTM1 + FAS* c.-1378G>A** |  |  |  |  |  |
| Present + AA | 48 | 13 (86.7) | 35 (94.6) | 2.69 (0.34-21.14) | 0.34 |
| Null + GA or GG | 4 | 2 (13.3) | 2 (5.4) | Reference |  |
| Present + AA or GA | 32 | 7 (70.0) | 25 (80.6) | 1.78 (0.35-9.02) | 0.48 |
| Null + GG | 9 | 3 (30.0) | 6 (19.4) | Reference |  |
| ***GSTM1 + FAS* c.-671A>G** |  |  |  |  |  |
| Present + GG | 37 | 10 (90.9) | 27 (79.4) | Reference | 0.40 |
| Null + AG or AA | 8 | 1 (9.1) | 7 (20.6) | 2.59 (0.28-23.80) |  |
| Present + GG or AG | 11 | 6 (50.0) | 5 (22.7) | Reference | 0.11 |
| Null + AA | 23 | 6 (50.0) | 17 (77.3) | 3.40 (0.75-15.36) |  |
| ***GSTM1 + FASL* c.-844C>T** |  |  |  |  |  |
| Present + TT | 39 | 11 (84.6) | 28 (75.7) | Reference | 0.50 |
| Null + CT or TT | 11 | 2 (15.4) | 9 (24.3) | 1.76 (0.32-9.51) |  |
| Present + TT or CT | 17 | 3 (33.3) | 14 (40.0) | 1.33 (0.28-6.23) | 0.71 |
| Null + CC | 27 | 6 (66.7) | 21 (60.0) | Reference |  |
| ***GSTT1 + GSTP1* c.313A>G** |  |  |  |  |  |
| Present + AA | 8 | 3 (27.3) | 5 (16.1) | Reference | 0.42 |
| Null + AG or GG | 34 | 8 (72.7) | 26 (83.9) | 1.95 (0.38-10.01) |  |
| Present + AA or AG |  | NE |  | NE | NE |
| Null + GG |  |  |  |  |  |
| ***GSTT1 + XPC* c.2815A>C** |  |  |  |  |  |
| Present + AA | 9 | 3 (27.3) | 6 (26.1) | Reference | 0.94 |
| Null + AC or CC | 25 | 8 (72.7) | 17 (73.9) | 1.06 (0.21-5.37) |  |
| ***GSTTI + XPD* c.934G>A** |  |  |  |  |  |
| Present + GG | 8 | 2 (20.0) | 6 (15.8) | Reference | 0.75 |
| Null + GA or AA | 40 | 8 (80.0) | 32 (84.2) | 1.33 (0.22-7.89) |  |
| Present + GG or GA | 1 | 1 (6.7) | 0 (0.0) |  |  |
| Null + AA | 66 | 14 (93.3) | 52 (100.0) | NE | NE |
| ***GSTT1 + XPD* c.2251A>C** |  |  |  |  |  |
| Present + AA | 8 | 3 (25.9) | 5 (15.2) | Reference | 0.44 |
| Null + AC or CC | 37 | 9 (75.0) | 28 (84.8) | 1.86 (0.37-9.39) |  |
| Present + AA or AC | 2 | 2 (11.1) | 0 (0.0) |  |  |
| Null + CC | 66 | 16 (88.9) | 50 (100.0) | NE | NE |
| ***GSTT1 + XPF* c.2505T>C** |  |  |  |  |  |
| Present + TT | 9 | 2 (22.2) | 7 (22.6) | 1.02 (0.17-6.07) | 0.98 |
| Null + TC or CC | 31 | 7 (77.8) | 24 (77.4) | Reference |  |
| Present + TT or TC | 2 | 1 (5.9) | 1 (1.9) | Reference | 0.42 |
| Null + CC | 67 | 16 (94.1) | 51 (98.1) | 3.18 (0.18-53.91) |  |
| ***GSTT1 + ERCC1* c.354C>T** |  |  |  |  |  |
| Present + CC | 11 | 3 (37.5) | 8 (34.8) | Reference | 0.89 |
| Null + CT or TT | 20 | 5 (62.5) | 1 (65.2) | 1.12 (0.21-5.96) |  |
| Present + CC or CT | 1 | 0 (0.0) | 1 (2.1) |  |  |
| Null | 58 | 12 (100.0) | 46 (97.9) | NE | NE |
| ***GSTT1 + MLH1* c.93G>A** |  |  |  |  |  |
| Present + GG | 7 | 3 (21.4) | 4 (10.8) | Reference | 0.33 |
| Null + GA or AA | 44 | 11 (78.6) | 33 (89.2) | 2.25 (0.43-11.65) |  |
| Present + GG or GA |  | NE | NE | NE | NE |
| Null + AA |  |  |  |  |  |
| ***GSTT1 + MSH2* c.211+9C>G** |  |  |  |  |  |
| Present + CC | 10 | 8 (44.4) | 2 (33.3) | Reference | 0.63 |
| Null + CG or GG | 14 | 10 (55.6) | 4 (66.7) | 1.60 (0.23-11.08) |  |
| Present + CC or CG | 4 | 3 (6.3) | 1 (8.3) | 1.36 (0.12-14.40) | 0.79 |
| Null + GG | 56 | 45 (93.8) | 11 (91.7) | Reference |  |
| ***GSTT1 + MSH3* c.3133G>A** |  |  |  |  |  |
| Present + GG | 14 | 4 (100.0) | 10 (66.7) |  |  |
| Null + GA + AA | 5 | 0 (0.0) | 5 (33.3) | NE | NE |
| Present + GG or GA | 7 | 2 (25.0) | 5 (17.9) | Reference | 0.65 |
| Null + AA | 29 | 6 (75.0) | 23 (82.1) | 1.53 (0.23-9.94) |  |
| ***GSTT1 + EXO1* c.1762G>A** |  |  |  |  |  |
| Present + GG | 7 | 2 (20.0) | 5 (19.2) | Reference | 0.95 |
| Null + GA or AA | 29 | 8 (80.0) | 21 (80.8) | 1.05 (0.16-6.55) |  |
| Present + GG or GA | 1 | 0 (0.0) | 1 (2.0) |  |  |
| Null + AA | 63 | 13 (100.0) | 50 (98.0) | NE | NE |
| ***GSTT1 + TP53* c.215G>C** |  |  |  |  |  |
| Present + CC | 14 | 5 (83.3) | 9 (56.3) | Reference | 0.26 |
| Null + GC or GG | 8 | 1 (16.7) | 7 (43.8) | 3.88 (0.36-41.32) |  |
| Present + CC or GC | 6 | 2 (18.2) | 4 (11.4) | Reference | 0.56 |
| Null + GG | 40 | 9 (81.8) | 31 (88.6) | 1.72 (0.27-10.98) |  |
| ***GSTT1 + CASP3* c.-1191A>G** |  |  |  |  |  |
| Present + GG | 14 | 4 (80.0) | 10 (58.8) | Reference | 0.39 |
| Null + AG or AA | 8 | 1 (20.0) | 7 (41.2) | 2.80 (0.25-30.70) |  |
| Present + GG or AG | 6 | 1 (10.0) | 5 (12.8) | 1.32 (0.13-12.80) | 0.80 |
| Null + AA | 43 | 9 (90.0) | 34 (87.2) | Reference |  |
| ***GSTT1 + CASP3* c.-182-247G>T** |  |  |  |  |  |
| Present + TT | 13 | 5 (71.4) | 8 (53.3) | Reference | 0.42 |
| Null + GT or GG | 9 | 2 (28.6)1 | 7 (46.7) | 2.18 (0.31-15.04) |  |
| Present + TT or GT | 5 | 2 (15.4) | 3 (8.3) | Reference | 0.10 |
| Null + GG | 44 | 11 (84.6) | 33 (91.7) | 4.95 (0.72-33.89) |  |
| ***GSTT1 + FAS* c.-1378G>A** |  |  |  |  |  |
| Present + AA | 13 | 4 (66.7) | 9 (81.8) | 2.25 (0.22-22.14) | 0.48 |
| Null + GA or GG | 4 | 2 (33.3) | 2 (18.2) | Reference |  |
| Present + AA or GA | 10 | 2 (22.2) | 8 (34.8) | 1.86 (0.31-11.18) | 0.49 |
| Null + GG | 22 | 7 (77.8) | 15 (65.2) | Reference |  |
| ***GSTT1 + FAS* c.-671A>G** |  |  |  |  |  |
| Present + GG | 12 | 4 (50.0) | 8 (36.4) | Reference | 0.50 |
| Null + AG or AA | 18 | 4 (50.0) | 14 (63.6) | 1.75 (0.34-8.98) |  |
| Present + GG or AG | 7 | 1 (9.1) | 6 (12.0) | 1.36 (0.14-12.62) | 0.78 |
| Null + AA | 54 | 10 (90.9) | 44 (88.0) | Reference |  |
| ***GSTT1 + FASL* c.-844C>T** |  |  |  |  |  |
| Present + TT | 13 | 4 (50.0) | 9 (36.0) | 1.77 (0.35-8.88) | 0.48 |
| Null + CT or CC | 20 | 4 (50.0) | 16 (64.0) | Reference |  |
| Present + TT or CT | 5 | 0 (0.0) | 5 (11.6) |  |  |
| Null + CC | 50 | 12 (100.0) | 38 (88.4) | NE | NE |
| ***GSTP1* c.313A>G *+ XPC* c.2815A>C** |  |  |  |  |  |
| AA + AA | 31 | 5 (55.6) | 26 (70.3) | 1.89 (0.42-8.40) | 0.40 |
| AG or GG + AC or CC | 15 | 4 (44.4) | 11 (29.7) | Reference |  |
| AA or AG + AA or AC | 1 | 0 (0.0) | 1 (1.8) |  |  |
| CC + CC | 72 | 17 (100.0) | 55 (98.2) | NE | NE |
| ***GSTP1* c.313A>G *+ XPD* c.934G>A** |  |  |  |  |  |
| AA + GG | 23 | 5 (50.0) | 18 (50.0) | 1.00 (0.24-4.06) | 1.00 |
| AG or GG + GA or AA | 23 | 5 (50.0) | 18 (50.0) | Reference |  |
| AA or AG + GG or GA | 2 | 0 (0.0) | 2 (3.2) |  |  |
| GG + AA | 77 | 17 (100.0) | 60 (96.8) | NE | NE |
| ***GSTP1* c.313A>G *+ XPD* c.2251A>C** |  |  |  |  |  |
| AA + AA | 23 | 6 (50.0) | 17 (54.8) | 1.21 (0.32-4.61) | 0.77 |
| AG or GG + AC or CC | 20 | 6 (50.0) | 14 (42.5) | Reference |  |
| AA or AG + AA or AC | 0 | 0 (0.0) | 2 (3.3) |  |  |
| AA + CC | 76 | 18 (100.0) | 58 (96.7) | NE | NE |
| ***GSTP1* c.313A>G *+ XPF* c.2505T>C** |  |  |  |  |  |
| AA + TT | 27 | 6 (54.4) | 21 (63.6) | 1.45 (0.36-5.81) | 0.59 |
| AG or GG + TC or CC | 17 | 5 (45.5) | 12 (36.4) | Reference |  |
| AA or AG + TT or TC | 1 | 0 (0.0) | 1 (1.7) |  |  |
| GG | 76 | 19 (100.0) | 57(98.3) | NE | NE |
| ***GSTP1* c.313A>G *+ ERCC1* c.354C>T** |  |  |  |  |  |
| AA + CC | 34 | 7 (70.0) | 27 (77.1) | 1.44 (0.30-6.92) | 0.64 |
| AG or GG + CT or TT | 11 | 3 (30.0) | 8 (2.9) | Reference |  |
| AA or AG + CC or CT | 1 | 0 (0.0) | 1 (1.9) |  |  |
| GG + TT | 68 | 16 (100.0) | 52 (98.1) | NE | NE |
| ***GSTP1* c.313A>G *+ MLH1* c.93G>A** |  |  |  |  |  |
| AA + GG | 18 | 3 (37.5) | 15 (45.5) | 1.38 (0.28-6.79) | 0.68 |
| AG or GG + GA or AA | 23 | 5 (62.5) | 18 (54.5) | Reference |  |
| AA or AG + GG or GA |  | NE | NE | NE | NE |
| GG + AA |  |  |  |  |  |
| ***GSTP1* c.313A>G *+ MSH2* c.211+9C>G** |  |  |  |  |  |
| AA + CC | 37 | 6 (75.0) | 31 (81.6) | 1.47 (0.24-8.91) | 0.67 |
| AG or GG + CG or GG | 9 | 2 (25.0) | 7 (18.2) | Reference |  |
| A or AG + CC or CG | 1 | 0 (0.0) | 1 (2.0) |  |  |
| GG + GG | 63 | 14 (100.0) | 49 (98.0) | NE | NE |
| ***GSTP1* c.313A>G *+ MSH3* c.3133G>A** |  |  |  |  |  |
| AA + GG | 47 | 11 (91.7) | 36 (87.8) | Reference | 0.71 |
| AG or GG + GA or AA | 6 | 1 (8.3) | 5 (12.5) | 1.52 (0.16-14.50) |  |
| AA or AG + GG or GA | 5 | 1 (10.0) | 4 (12.5) | 1.28 (0.12-13.03) | 0.83 |
| GG + AA | 37 | 9 (90.0) | 28 (87.5) | Reference |  |
| ***GSTP1* c.313A>G *+ EXO1* c.1762G>A** |  |  |  |  |  |
| AA + GG | 27 | 5 (50.0) | 22 (64.7) | 1.83 (0.44-7.62) | 0.40 |
| AG or GG + GA or AA | 17 | 5 (50.0) | 12 (35.3)0 | Reference |  |
| AA or AG + GG or GA | 1 | 1 (5.3) | 0 (0.0) |  |  |
| GG or AA | 73 | 18 (94.7) | 55 (100.0) | NE | NE |
| ***GSTP1* c.313A>G *+ TP53* c.215G>C** |  |  |  |  |  |
| AA + CC | 42 | 10 (100.0) | 32 (88.9) |  |  |
| AG or GG + GC or CC | 4 | 0 (0.0) | 4 (11.1) | NE | NE |
| AA or AG + CC or GC | 3 | 1 (7.7) | 2 (5.4) | Reference | 0.76 |
| GG + GG | 47 | 12 (92.3) | 35 (94.6) | 1.45 (0.12-17.56) |  |
| ***GSTP1* c.313A>G *+ CASP3* c.-1191A>G** |  |  |  |  |  |
| AA + GG | 41 | 10 (90.9) | 31 (93.9) | 1.55 (0.12-18.95) | 0.73 |
| AG or GG + AG or AA | 3 | 1 (9.1) | 2 (6.1) | Reference |  |
| AA or AG or GG or AG | 4 | 1 (7.1) | 3 (7.3) | 1.02 (0.09-10.75) | 0.98 |
| GG + AA | 51 | 13 (92.9) | 38 (92.7) | Reference |  |
| ***GSTP1* c.313A>G *+ CASP3* c.-182-247G>T** |  |  |  |  |  |
| AA + TT | 42 | 10 (90.9) | 32 (86.5) | Reference | 0.69 |
| AG or GG + GT or GG | 6 | 1 (9.1) | 5 (13.5) | 1.56 (0.16-14.99) |  |
| AA or AG + TT or GT | 2 | 1 (6.7) | 1 (2.6) | Reference | 0.50 |
| GG + GG | 51 | 14 (93.3) | 37 (97.4) | 2.64 (0.15-45.19) |  |
| ***GSTP1* c.313A>G *+ FAS* c.-1378G>A** |  |  |  |  |  |
| AA + AA | 44 | 10 (83.3) | 34 (97.1) | 6.80 (0.55-82.99) | 0.13 |
| AG or GG + GA or GG | 3 | 2 (16.7) | 1 (2.9) | Reference |  |
| AA or AG + AA or GA | 3 | 0 (0.0) | 3 (15.8) |  |  |
| GG + GG | 25 | 9 (100.0) | 16 (84.3) | NE | NE |
| ***GSTP1* c.313A>G *+ FASL* c.-844C>T** |  |  |  |  |  |
| AA + TT | 37 | 8 (80.0) | 29 (74.4) | Reference | 0.71 |
| AG or GG + CT or CC | 12 | 2 (20.0) | 10 (25.6) | 1.37 (0.25-7.61) |  |
| AA or AG + TT or CT | 1 | 0 (0.0) | 1 (2.2) |  |  |
| GG + CC | 58 | 13 (100.0) | 45 (97.8) | NE | NE |
| ***XPC* c.2815A>C *+ XPD* c.934G>A** |  |  |  |  |  |
| AA + GG | 28 | 5 (50.0) | 23 (63.9) | 1.76 (0.43-7.27) | 0.42 |
| AC or CC + GA or AA | 18 | 5 (50.0) | 13 (36.1) | Reference |  |
| AA or AC + GG or GA | 2 | 2 (10.5) | 0 (0.0) |  |  |
| GG + AA | 70 | 17 (89.5) | 53 (100.0) | NE | NE |
| ***XPC* c.2815A>C *+ XPD* c.2251A>C** |  |  |  |  |  |
| AA + AA | 31 | 5 (50.0) | 26 (66.7) | 2.00 (0.49-8.16) | 0.33 |
| AC or CC + AC or CC | 18 | 5 (50.0) | 13 (33.3) | Reference |  |
| AA or AC + AA or AC | 1 | 0 (0.0) | 1 (1.9) |  |  |
| CC or CC | 68 | 16 (100.0) | 52 (98.1) | NE | NE |
| ***XPC* c.2815A>C *+ ERCC1* c.354C>T** |  |  |  |  |  |
| AA + CC | 39 | 6 (75.0) | 33 (86.8) | 2.75 (0.40-18.52) | 0.40 |
| AC or CC + CT or TT | 6 | 2 (25.0) | 5 (13.2) | Reference |  |
| AA or AC + CC or CT |  | NE | NE | NE | NE |
| CC or TT |  |  |  |  |  |
| ***XPC* c.2815A>C *+ MLH1* c.93G>A** |  |  |  |  |  |
| AA + GG | 25 | 4 (40.0) | 21 (60.0) | 2.25 (0.53-9.44) | 0.26 |
| AC or CC + GA or AA | 20 | 6 (60.0) | 14 (40.0) | Reference |  |
| AA or AC + GG or GA |  | NE | NE | NE | NE |
| CC or AA |  |  |  |  |  |
| ***XPC* c.2815A>C *+ MSH2* c.211+9C>G** |  |  |  |  |  |
| AA + CC | 45 | 7 (70.0) | 38 (90.5) | 4.07 (0.75-22.29) | 0.10 |
| AC or CC + GC or GG | 7 | 3 (30.0) | 4 (9.5) | Reference |  |
| AA or AC + CC or GC | 5 | 1 (7.1) | 4 (7.8) | 1.10 (0.11-10.77) | 0.93 |
| CC + GG | 60 | 13 (92.9) | 47 (92.2) | Reference |  |
| ***XPC* c.2815A>C *+ MSH3* c.3133G>A** |  |  |  |  |  |
| AA + GG | 52 | 10 (100.0) | 42 (97.7) |  |  |
| AC or CC + AG or AA | 1 | 0 (0.0) | 1 (2.3) | NE | NE |
| AA or AC + AG or GG | 6 | 2 (20.0) | 4 (14.8) | Reference | 0.70 |
| CC+ AA | 31 | 8 (80.0) | 23 (85.2) | 1.43 (0.22-9.40) |  |
| ***XPC* c.2815A>C *+ EXO1* c.1762G>A** |  |  |  |  |  |
| AA + GG | 30 | 5 (50.0) | 25 (83.3) | **5.00 (1.04-23.98)** | **0.04** |
| AC or CC + GA or AA | 10 | 5 (50.0) | 5 (16.7) | Reference |  |
| AA or AC + GG or GA | 1 | 1 (5.9) | 0 (0.0) |  |  |
| CC + AA | 66 | 16 (94.1) | 50 (100.0) | NE | NE |
| ***XPC* c.2815A>C *+ TP53* c.215G>C** |  |  |  |  |  |
| AA + CC | 52 | 10 (100.0) | 42 (91.3) |  |  |
| AC or CC + GC or CC | 4 | 0 (0.0) | 4 (8.7) | NE | NE |
| AA or AC + GC or CC | 5 | 1 (9.1) | 4 (11.1) | 1.25 (0.12-12.51) | 0.84 |
| CC + GG | 42 | 10 (90.9) | 32 (88.9) | Reference |  |
| ***XPC* c.2815A>C *+ CASP3* c.-1191A>G** |  |  |  |  |  |
| AA + GG | 49 | 9 (100.0) | 40 (97.6) |  |  |
| AC or CC + GG or AG | 1 | 0 (0.0) | 1 (2.4) | NE | NE |
| AA or AC + GG or AG | 4 | 1 (8.3) | 3 (8.3) | Reference | 1.00 |
| CC + AA | 44 | 11 (91.7) | 33 (91.7) | 1.00 (0.09-10.63) |  |
| ***XPC* c.2815A>C *+ CASP3* c.-182-247G>T** |  |  |  |  |  |
| AA + TT | 48 | 10 (90.9) | 38 (97.4) | 0.26 (0.01-4.58) | 0.36 |
| AC or CC + GT or GG | 2 | 1 (9.1) | 1 (2.6) | Reference |  |
| AA or AC + TT or GT | 7 | 1 (7.7) | 6 (14.0) | 1.63 (0.26-10.16) | 0.59 |
| CC + GG | 49 | 12 (92.3) | 37 (86.0) | Reference |  |
| ***XPC* c.2815A>C *+ FAS* c.-1378G>A** |  |  |  |  |  |
| AA + AA | 54 | 2 (16.7) | 1 (2.2) | Reference | 0.08 |
| AC or CC+ GA or GG | 3 | 10 (83.3) | 44 (97.8) | 8.80 (0.72-106.85) |  |
| AA or AC+ GA or AA | 8 | 1 (11.1) | 7 (25.8) | 3.73 (0.38-35.92) | 0.25 |
| CC + GG | 23 | 8 (88.9) | 15 (68.2) | Reference |  |
| ***XPC* c.2815A>C *+ FAS* c.-671A>G** |  |  |  |  |  |
| AA + GG | 45 | 9 (75.0) | 36 (85.7) | 2.00 (0.41-9.58) | 0.38 |
| AC or CC+ AG or AA | 9 | 3 (25.0) | 6 (14.3) | Reference |  |
| AA or AC+ GG or AG | 5 | 1 (7.7) | 4 (8.5) | 1.11 (0.11-10.94) | 0.92 |
| CC + AA | 55 | 12 (92.3) | 43 (91.5) | Reference |  |
| ***XPC* c.2815A>C *+ FASL* c.-844C>T** |  |  |  |  |  |
| AA + TT | 45 | 9 (75.0) | 36 (83.7) | 1.71 (0.36-7.97) | 0.49 |
| AC or CC + CT or CC | 10 | 3 (25.0) | 7 (16.3) | Reference |  |
| AA or AC + CT or TT | 6 | 1 (6.3) | 5 (11.4) | 1.92 (0.20-17.85) | 0.56 |
| CC + CC | 54 | 15 (93.8) | 39 (88.6) | Reference |  |
| ***XPD* c.934G>A *+ XPD* c.2251A>C** |  |  |  |  |  |
| GG + AA | 33 | 7 (46.7) | 26 (48.1) | 1.06 (0.33-3.33) | 0.91 |
| GA or AA + AC or CC | 36 | 8 (53.3) | 28 (51.9) | Reference |  |
| GG or AG + AA or AC | 5 | 1 (5.6) | 4 (6.3) | 1.15 (0.12-11.00) | 0.90 |
| AA + CC | 76 | 17 (94.4) | 59 (93.7) | Reference |  |
| ***XPD* c.934G>A *+ XPF* c.2505T>C** |  |  |  |  |  |
| GG + TT | 26 | 5 (50.0) | 21 (55.3) | 1.23 (0.30-4.98) | 0.76 |
| AG or AA + TC or CC | 22 | 5 (50.0) | 17 (44.7) | Reference |  |
| GG or AG + TT or TC | 3 | 0 (0.0) | 3 (4.9) |  |  |
| AA + CC | 75 | 17 (100.0) | 58 (95.1) | NE | NE |
| ***XPD* c.934G>A *+ ERCC1* c.354C>T** |  |  |  |  |  |
| GG + CC | 28 | 7 (63.6) | 21 (75.0) | 1.71 (0.38-7.65) | 0.48 |
| AG or AA + CT or TT | 11 | 4 (36.4) | 7 (25.0) | Reference |  |
| GG or AG + CC or CT |  | NE | NE | NE | NE |
| AA + TT |  |  |  |  |  |
| ***XPD* c.934G>A *+ MLH1* c.93G>A** |  |  |  |  |  |
| GG + GG | 16 | 2 (28.6) | 14 (38.9) | 1.59 (0.27-9.35) | 0.60 |
| GA or AA + GA or AA | 27 | 5 (71.4) | 22 (61.1) | Reference |  |
| GG or GA + GG or GA |  | NE | NE | NE | NE |
| AA + AA |  |  |  |  |  |
| ***XPD* c.934G>A *+ MSH2* c.211+9C>G** |  |  |  |  |  |
| GG + CC | 34 | 8 (61.5) | 26 (78.8) | 2.32 (0.57-9.36) | 0.23 |
| GA or AA + CG or GG | 12 | 5 (38.5) | 7 (21.2) | Reference |  |
| GG or AG + CC or CG | 2 | 1 (7.1) | 1 (2.0) | Reference | 0.36 |
| AA + GG | 61 | 13 (92.9) | 48 (98.0) | 3.69 (0.21-63.11) |  |
| ***XPD* c.934G>A *+ MSH3* c.3133G>A** |  |  |  |  |  |
| GG + GG | 38 | 9 (100.0) | 29 (90.6) |  |  |
| GA or AA + GA or AA | 3 | 0 (0.0) | 3 (9.4) | NE | NE |
| GG or GA + GA or GG | 5 | 1 (12.5) | 4 (12.9) | Reference | 0.97 |
| AA + AA | 34 | 7 (87.5) | 27 (97.1) | 1.03 (1.00-10.80) |  |
| ***XPD* c.934G>A *+ EXO1* c.1765G>A** |  |  |  |  |  |
| GG + GG | 23 | 4 (44.4) | 19 (57.6) | 1.69 (0.38-7.49) | 0.48 |
| GA or AA + GA or AA | 19 | 5 (55.6) | 14 (42.4) | Reference |  |
| GG or GA + GG or GA | 1 | 0 (0.0) | 1 (1.8) | NE | NE |
| AA + AA | 70 | 15 (100.0) | 55 (98.2) |  |  |
| ***XPD* c.934G>A *+ TP53* c.215G>C** |  |  |  |  |  |
| GG + CC | 36 | 9 (100.0) | 27 (87.1) |  |  |
| GA or AA + GC or GG | 4 | 0 (0.0) | 4 (12.9) | NE | NE |
| GG or GA + CC or GC | 2 | 1 (9.1) | 1 (2.9) | Reference | 0.41 |
| AA + GG | 43 | 10 (90.9) | 33 (97.1) | 3.30 (0.18-57.66) |  |
| ***XPD* c.934G>A *+ CASP3* c.-1191A>G** |  |  |  |  |  |
| GG + GG | 38 | 10 (83.3) | 28 (87.5) | 1.40 (0.22-8.85) | 0.72 |
| GA or AA + AG or AA | 6 | 2 (16.7) | 4 (12.5) | Reference |  |
| GG or GA + GG or AG | 4 | 1 (8.3) | 3 (7.5) | Reference | 0.92 |
| AA + AA | 48 | 11 (91.7) | 37 (92.5) | 1.12 (0.10-11.88) |  |
| ***XPD* c.934G>A *+ CASP3* c.-182-247G>T** |  |  |  |  |  |
| GG + TT | 37 | 10 (83.3) | 27 (84.4) | 1.08 (0.18-6.48) | 0.93 |
| AG or AA + GT or GG | 7 | 2 (16.7) | 5 (15.6) | Reference |  |
| GG or AG + TT or GT | 3 | 1 (9.1) | 2 (5.0) | Reference | 0.61 |
| AA + GG | 49 | 12 (92.3) | 37 (94.9) | 1.90 (0.15-23.13) |  |
| ***XPD* c.934G>A *+ FAS* c.-1378G>A** |  |  |  |  |  |
| GG + AA |  | NE | NE | NE | NE |
| GA or AA + GA or GG |  |  |  |  |  |
| GG or GA + AA or GA | 4 | 1 (11.1) | 3 (16.7) | 1.60 (0.14-18.00) | 0.70 |
| AA +GG | 23 | 8 (88.9) | 15 (83.3) | Reference |  |
| ***XPD* c.934G>A *+ FAS* c.-671A>G** |  |  |  |  |  |
| GG + GG | 28 | 6 (85.7) | 22 (75.9) | Reference | 0.57 |
| GA or AA + AG or AA | 8 | 1 (14.3) | 7 (24.1) | 1.90 (0.19-18.69) |  |
| GG or GA + GG or AG | 3 | 2 (13.3) | 1 (2.2) | Reference | 0.13 |
| AA + AA | 57 | 13 (86.7) | 44 (97.8) | 6.76 (0.56-80.74) |  |
| ***XPD* c.934G>A *+ FASL* c.-844C>T** |  |  |  |  |  |
| GG + TT | 30 | 7 (77.8) | 23 (71.9) | Reference | 0.72 |
| AG or AA + CT or TT | 11 | 2 (22.2) | 9 (28.1) | 1.37 (0.23-7.88) |  |
| GG or AG + TT or CT | 4 | 1 (6.3) | 3 (6.8) | 1.09 (0.10-11.38) | 0.93 |
| AA + CC | 56 | 15 (93.8) | 41 (93.2) | Reference |  |
| ***XPD* c.2251A>C *+ XPF* c.2505T>C** |  |  |  |  |  |
| AA + TT | 27 | 4 (50.0) | 23 (59.0) | 1.43 (0.31-6.61) | 0.64 |
| AC or CC + TC or CC | 20 | 4 (50.0) | 16 (41.0) | Reference |  |
| AA or AC + TT or TC | 3 | 0 (0.0) | 3 (5.1) | NE | NE |
| CC + CC | 74 | 18 (100.0) | 56 (94.9) |  |  |
| ***XPD* c.2251A>C *+ ERCC1* c.354C>T** |  |  |  |  |  |
| AA + CC | 27 | 7 (63.6) | 20 (87.0) | 3.81 (0.67-21.41) | 0.12 |
| AC or CC + CT or TT | 7 | 4 (36.4) | 3 (13.0) | Reference |  |
| AA or AC + CC or CT |  | NE | NE | NE | NE |
| CC or TT |  |  |  |  |  |
| ***XPD* c.2251A>C *+ MLH1* c.93G>A** |  |  |  |  |  |
| AA + GG | 18 | 3 (33.3) | 15 (42.9) | 1.50 (0.32-6.99) | 0.60 |
| AC or CC + GA or AA | 26 | 6 (66.7) | 20 (57.1) | Reference |  |
| AA or AC + GG or GA |  | NE | NE | NE | NE |
| CC or AA |  |  |  |  |  |
| ***XPD* c.2251A>C *+ MSH2* c.211+9C>G** |  |  |  |  |  |
| AA + CC | 36 | 7 (63.6) | 29 (80.6) | 2.36 (0.53-10.40) | 0.25 |
| AC or CC + CG or GG | 11 | 4 (36.4) | 7 (19.4) | Reference |  |
| AA or AC + CC or CG | 2 | 0 (0.0) | 2 (4.1) | NE | NE |
| CC + GG | 60 | 13 (100.0) | 47 (95.9) |  |  |
| ***XPD* c.2251A>C *+ MSH3* c.3133G>A** |  |  |  |  |  |
| AA + GG | 41 | 9 (100.0) | 32 (91.4) |  |  |
| AC or CC + GA or AA | 3 | 0 (0.0) | 3 (8.6) | NE | NE |
| AA or AC + GG or GA | 6 | 1 (11.1) | 5 (16.1) | 1.53 (0.15-15.17) | 0.71 |
| CC + AA | 34 | 8 (88.9) | 26 (83.9) | Reference |  |
| ***XPD* c.2251A>C *+ EXO1* c.1765G>A** |  |  |  |  |  |
| AA + GG | 23 | 3 (42.9) | 20 (62.5) | 2.22 (0.42-11.67) | 0.34 |
| AC or CC + GA or AA | 16 | 4 (57.1) | 12 (37.5) | Reference |  |
| AA or AC + GG or GA | 1 | 0 (0.0) | 1 (1.9) | NE | NE |
| CC + AA | 69 | 16 (100.0) | 53 (98.1) |  |  |
| ***XPD* c.2251A>C *+ TP53* c.215G>C** |  |  |  |  |  |
| AA + CC | 41 | 10 (90.9) | 31 (86.1) | Reference | 0.67 |
| AC or CC + GC or GG | 6 | 1 (9.1) | 5 (13.9) | 1.61 (0.16-15.49) |  |
| AA or AC + CC or GC | 3 | 1 (8.3) | 2 (5.9) | Reference | 0.76 |
| CC + GG | 43 | 11 (91.7) | 32 (94.1) | 1.45 (0.12-17.65) |  |
| ***XPD* c.2251A>C *+ CASP3* c.-1191A>G** |  |  |  |  |  |
| AA + GG | 41 | 10 (83.3) | 31 (88.6) | 1.55 (0.24-9.76) | 0.64 |
| AC or CC + AG or AA | 6 | 2 (16.7) | 4 (11.4) | Reference |  |
| AA or AC + GG or AG | 4 | 1 (7.7) | 3 (7.9) | 1.02 (0.09-10.85) | 0.98 |
| CC + AA | 47 | 12 (92.3) | 35 (92.1) | Reference |  |
| ***XPD* c.2251A>C *+ FAS* c.-1378G>A** |  |  |  |  |  |
| AA + AA |  | NE | NE | NE | NE |
| AC or CC + GA or GG |  |  |  |  |  |
| AA or AC + AA or GA | 7 | 1 (10.0) | 6 (27.3) | 3.37 (0.34-32.63) | 0.29 |
| CC + GG | 25 | 9 (90.0) | 16 (72.7) | Reference |  |
| ***XPD* c.2251A>C *+ FAS* c.-671A>G** |  |  |  |  |  |
| AA + GG | 30 | 7 (77.8) | 23 (82.1) | 1.31 (0.20-8.31) | 0.77 |
| AC or CC + AG or AA | 7 | 2 (22.2) | 5 (17.9) | Reference |  |
| AA or AC + GG or AG | 3 | 1 (7.1) | 2 (4.4) | Reference | 0.69 |
| CC + AA | 56 | 13 (92.9) | 43 (95.6) | 1.65 (0.13-19.73) |  |
| ***XPD* c.2251A>C *+ FASL* c.-844C>T** |  |  |  |  |  |
| AA + TT | 35 | 9 (69.2) | 26 (74.7) | 1.28 (0.31-5.21) | 0.72 |
| AC or CC + CT or CC | 13 | 4 (30.8) | 9 (25.7) | Reference |  |
| AA or AC + TT or CT | 2 | 0 (0.0) | 2 (5.0) | NE | NE |
| CC + CC | 53 | 15 (100.0) | 38 (95.0) |  |  |
| ***XPF* c.2505T>C *+ ERCC1* c.354C>T** |  |  |  |  |  |
| TT + CC | 36 | 6 (75.0) | 30 (81.1) | 1.42 (0.23-8.63) | 0.69 |
| TC or CC + CT or TT | 9 | 2 (25.0) | 7 (18.9) | Reference |  |
| TT or TC + CC or CT |  | NE | NE | NE | NE |
| CC or TT |  |  |  |  |  |
| ***XPF* c.2505T>C *+ MLH1* c.93G>A** |  |  |  |  |  |
| TT + GG | 20 | 3 (37.5) | 17 (51.5) | 1.77 (0.36-8.64) | 0.48 |
| TC or CC + GA or AA | 21 | 5 (62.5) | 16 (48.5) | Reference |  |
| TT or TC + GG or GA | 1 | 0 (0.0) | 1 (1.7) | NE | NE |
| CC or AA | 77 | 18 (100.0) | 59 (98.3) |  |  |
| ***XPF* c.2505T>C *+ MSH3* c.3133G>A** |  |  |  |  |  |
| TT + GG | 49 | 11 (91.7) | 38 (92.7) | 1.15 (0.10-12.20) | 0.90 |
| TC or CC + GA or AA | 4 | 1 (8.3) | 3 (7.3) | Reference |  |
| TT or TC + GG or GA | 5 | 1 (10.0) | 4 (13.8) | 1.44 (0.14-14.65) | 0.75 |
| CC + AA | 34 | 9 (90.0) | 25 (86.2) | Reference |  |
| ***XPF* c.2505T>C *+ EXO1* c.1765G>A** |  |  |  |  |  |
| TT + GG | 31 | 5 (50.0) | 26 (68.4) | 2.16 (0.52-8.92) | 0.28 |
| TC or CC + GA or AA | 17 | 5 (50.0) | 12 (31.6) | Reference |  |
| TT or TC + GG or GA | 1 | 0 (0.0) | 1 (1.9) | NE | NE |
| CC + AA | 70 | 17 (100.0) | 53 (98.1) |  |  |
| ***XPF* c.2505T>C *+ CASP3* c.-182-247G>T** |  |  |  |  |  |
| TT + TT | 46 | 11 (84.6) | 35 (89.7) | 1.59 (0.25-9.89) | 0.61 |
| TC or CC + GT or GG | 6 | 2 (15.4) | 4 (10.3) | Reference |  |
| TT or TC + TT or GT | 5 | 1 (7.7) | 4 (9.5) | 1.51 (0.15-14.73) | 0.84 |
| CC + GG | 51 | 12 (92.3) | 38 (90.5) | Reference |  |
| ***XPF* c.2505T>C *+ FAS* c.-1378G>A** |  |  |  |  |  |
| TT + AA | 48 | 10 (83.3) | 38 (97.4) | 7.60 (0.62-92.53) | 0.11 |
| TC or CC + GA or GG | 3 | 2 (16.7) | 1 (2.6) | Reference |  |
| TT or TC + AA or GA | 6 | 1 (9.1) | 5 (25.0) | 3.33 (0.33-32.95) | 0.30 |
| CC + GG | 25 | 10 (90.9) | 15 (75.0) | Reference |  |
| ***XPF* c.2505T>C *+ FASL* c.-844C>T** |  |  |  |  |  |
| TT + TT | 40 | 8 (80.0) | 32 (78.0) | Reference | 0.89 |
| TC or CC + CT or CC | 11 | 2 (20.0) | 9 (22.0) | 1.12 (0.20-6.26) |  |
| TT or TC + TT or CT | 3 | 0 (0.0) | 3 (7.1) | NE | NE |
| CC or CC | 55 | 16 (100.0) | 39 (92.9) |  |  |
| ***ERCC1* c.354C>T *+ MLH1* c.93G>A** |  |  |  |  |  |
| CC+ GG | 25 | 4 (57.1) | 21 (67.7) | 1.57 (0.29-8.41) | 0.59 |
| CT or TT + GA or AA | 13 | 3 (42.9) | 10 (32.3) | Reference |  |
| CC or CT + GG or GA | 1 | 1 (5.9) | 0 (0.0) |  |  |
| TT + AA | 69 | 16 (94.1) | 53 (100.0) | NE | NE |
| ***ERCC1* c.354C>T *+ MSH2* c.211+9C>G** |  |  |  |  |  |
| CC + CC | 50 | 10 (76.9) | 40 (95.2) | 6.00 (0.88-40.87) | 0.06 |
| CT or TT + CG or GG | 5 | 3 (23.1) | 2 (4.8) | Reference |  |
| CC or CT+ CC or CG | 3 | 1 (7.7) | 2 (4.5) | Reference | 0.65 |
| TT + GG | 54 | 12 (92.3) | 42 (95.5) | 1.75 (0.14-20.99) |  |
| ***ERCC1* c.354C>T *+ MSH3* c.3133G>A** |  |  |  |  |  |
| CC + GG | 59 | 13 (100.0) | 46 (97.9) |  |  |
| CT or TT + GA or AA | 1 | 0 (0.0) | 1 (2.1) | NE | NE |
| CC or CT+ GG or GA | 12 | 3 (27.3) | 9 (26.5) | Reference | 0.95 |
| TT + AA | 33 | 8 (72.7) | 25 (73.5) | 1.04 (0.22-4.81) |  |
| ***ERCC1* c.354C>T *+ EXO1* c.1765G>A** |  |  |  |  |  |
| CC + GG | 37 | 7 (63.6) | 30 (83.3) | 2.85 (0.63-12.92) | 0.17 |
| CT or TT+ GA or AA | 10 | 4 (36.4) | 6 (16.7) | Reference |  |
| CC or CT + GG or GA | 3 | 0 (0.0) | 3 (5.7) | NE | NE |
| TT + AA | 64 | 14 (100.0) | 50 (94.3) |  |  |
| ***ERCC1* c.354C>T *+ TP53* c.215G>C** |  |  |  |  |  |
| CC + CC | 58 | 14 (93.3) | 44 (95.7) | 1.21 (0.22-6.65) | 0.72 |
| CT or TT + GC or GG | 3 | 1 (6.7) | 2 (4.3) | Reference |  |
| CC or CT + CC or GC | 10 | 2 (16.7) | 8 (19.5) | 1.21 (0.22-6.65) | 0.82 |
| TT + GG | 43 | 10 (83.3) | 33 (80.5) | Reference |  |
| ***ERCC1* c.354C>T *+ CASP3* c.-1191A>G** |  |  |  |  |  |
| CC + GG | 59 | 13 (92.9) | 46 (93.9) | 1.17 (0.11-12.31) | 0.89 |
| CT or TT+ AG or AA | 4 | 1 (7.1) | 3 (6.1) | Reference |  |
| CC or CT+ GG or AG | 7 | 2 (15.4) | 5 (13.5) | Reference | 0.86 |
| TT + AA | 43 | 11 (84.6) | 32 (86.5) | 1.16 (0.19-6.88) |  |
| ***ERCC1* c.354C>T *+ CASP3* c.-182-247G>T** |  |  |  |  |  |
| CC + TT | 56 | 13 (92.9) | 43 (95.6) | 1.65 (0.13-19.73) | 0.69 |
| CT or TT+ GT or GG | 3 | 1 (7.1) | 2 (4.4) | Reference |  |
| CC or CT+ TT or GT | 5 | 1 (8.3) | 4 (11.1) | 1.37 (0.13-13.65) | 0.80 |
| TT + GG | 43 | 11 (91.7) | 32 (88.9) | Reference |  |
| ***ERCC1* c.354C>T *+ FAS* c.-1378G>A** |  |  |  |  |  |
| CC + AA |  | NE | NE | NE | NE |
| CT or TT + GA or GG |  |  |  |  |  |
| CC or CT+ AA or GA | 12 | 2 (20.0) | 10 (40.0) | 2.66 (0.46-15.25) | 0.27 |
| TT + GG | 23 | 8 (80.0) | 15 (60.0) | Reference |  |
| ***ERCC1* c.354C>T *+ FAS* c.-671A>G** |  |  |  |  |  |
| CC + GG | 48 | 11 (84.6) | 37 (92.5) | 2.24 (0.33-15.16) | 0.40 |
| CT or TT+ AG or AA | 5 | 2 (15.4) | 3 (7.5) | Reference |  |
| CC or CT + GG or AG | 4 | 2 (14.3) | 2 (5.0) | Reference | 0.27 |
| TT + AA | 50 | 12 (85.7) | 38 (95.0) | 3.16 (0.40-24.96) |  |
| ***ERCC1* c.354C>T *+ FASL* c.-844C>T** |  |  |  |  |  |
| CC + TT | 46 | 10 (90.9) | 36 (92.3) | 1.20 (0.11-12.86) | 0.88 |
| CT or TT+ CT or CC | 4 | 1 (9.1) | 3 (7.7) | Reference |  |
| CC or CT+ TT or CT | 8 | 2 (11.8) | 6 (14.0) | 1.21 (0.22-6.71) | 0.82 |
| TT + CC | 52 | 15 (88.2) | 37 (86.0) | Reference |  |
| ***MLH1* c.93G>A *+ MSH2* c.211+9C>G** |  |  |  |  |  |
| GG + CC | 27 | 5 (55.6) | 22 (78.6) | 2.93 (0.59-14.45) | 0.18 |
| GA or AA + GC or GG | 10 | 4 (44.4) | 6 (21.4) | Reference |  |
| GG or GA + CC or CG | 1 | 1 (6.7) | 0 (0.0) |  |  |
| AA + GG | 64 | 14 (93.3) | 50 (100.0) | NE | NE |
| ***MLH1* c.93G>A *+ MSH3* c.3133G>A** |  |  |  |  |  |
| GG + GG | 34 | 8 (88.9) | 26 (89.7) | 1.08 (0.09-11.91) | 0.94 |
| GA or AA + GA or AA | 4 | 1 (11.1) | 3 (10.3) | Reference |  |
| GG or AG + GG or GA | 2 | 1 (11.1) | 1 (3.6) | Reference | 0.94 |
| AA + AA | 35 | 8 (88.9) | 27 (96.7) | 1.08 (0.09-11.91) |  |
| ***MLH1* c.93G>A *+ EXO1* c.1762G>A** |  |  |  |  |  |
| GG + GG | 18 | 4 (36.4) | 14 (53.8) | 2.04 (0.47-8.70) | 0.33 |
| AG or AA + GA or AA | 19 | 7 (63.6) | 12 (46.2) | Reference |  |
| GG or GA + GG or GA | 2 | 1 (1.1) | 1 (3.6) |  |  |
| AA + AA | 35 | 8 (88.9) | 27 (96.4) | NE | NE |
| ***MLH1* c.93G>A *+ TP53* c.215G>C** |  |  |  |  |  |
| GG + CC | 32 | 8 (88.9) | 24 (85.7) | Reference | 0.80 |
| GA or AA + GC or GG | 5 | 1 (11.1) | 4 (14.3) | 1.33 (0.12-13.74) |  |
| GG or GA + CC or GC |  | NE | NE | NE | NE |
| AA + GG |  |  |  |  |  |
| ***MLH1* c.93G>A *+ CASP3* c.-1191A>G** |  |  |  |  |  |
| GG + GG | 30 | 7 (87.5) | 23 (92.0) | 1.64 (0.12-20.93) | 0.70 |
| GA or AA + AG or AA | 3 | 1 (12.5) | 2 (8.0) | Reference |  |
| GG or AG or GG or AG | 2 | 1 (7.7) | 1 (2.6) | Reference | 0.42 |
| AA + AA | 50 | 12 (92.3) | 38 (97.4) | 3.16 (0.18-54.56) |  |
| ***MLH1* c.93G>A *+ CASP3* c.-182-247G>T** |  |  |  |  |  |
| GG + TT | 30 | 6 (100.0) | 24 (82.8) |  |  |
| GA or AA + GT or TT | 5 | 0 (0.0) | 5 (17.2) | NE | NE |
| GG or AG + TT or GT | 2 | 1 (8.3) | 1 (2.4) | Reference | 0.37 |
| AA + GG | 52 | 11 (91.7) | 40 (97.6) | 3.63 (0.21-62.92) |  |
| ***MLH1* c.93G>A *+ FAS* c.-1378G>A** |  |  |  |  |  |
| GG + AA | 35 | 8 (72.7) | 27 (93.1) | 5.06 (0.71-35.77) | 0.10 |
| AG or AA + GA or GG | 5 | 3 (27.3) | 2 (6.9) | Reference |  |
| GG or AG + AA or GA | 2 | 1 (10.0) | 1 (5.9) | Reference | 0.69 |
| AA + GG | 25 | 9 (90.0) | 16 (94.1) | 1.77 (0.09-31.97) |  |
| ***MLH1* c.93G>A *+ FAS* c.-671A>G** |  |  |  |  |  |
| GG + GG | 27 | 7 (63.6) | 20 (71.4) | 1.42 (0.32-6.25) | 0.63 |
| GA or AA + AG or AA | 12 | 4 (36.4) | 8 (28.6) | Reference |  |
| GG or GA + GG or AG | 1 | 1 (7.1) | 0 (0.0) |  |  |
| AA + AA | 59 | 13 (92.9) | 46 (100.0) | NE | NE |
| ***MLH1* c.93G>A *+ FASL* c.-844C>T** |  |  |  |  |  |
| GG + TT | 26 | 7 (63.6) | 19 (70.4) | 1.35 (0.30-5.96) | 0.68 |
| AG or AA + CT or TT | 12 | 4 (36.4) | 8 (29.6) | Reference |  |
| GG or AG + TT or CT | 1 | 1 (5.9) | 0 (0.0) |  |  |
| AA + CC | 57 | 16 (94.1) | 41 (100.0) | NE | NE |
| ***MSH2* c.211+9C>G *+ MSH3* c.3133G>A** |  |  |  |  |  |
| CC + GG | 65 | 13 (100.0) | 52 (96.3) |  |  |
| CG or GG + GA or AA | 2 | 0 (0.0) | 2 (3.7) | NE | NE |
| CC or GC + GG or AG | 12 | 3 (33.3) | 9 (29.0) | Reference | 0.84 |
| GG + AA | 28 | 6 (66.7) | 22 (71.0) | 1.22 (0.25-5.98) |  |
| ***MSH2* c.211+9C>G *+ EXO1* c.1765G>A** |  |  |  |  |  |
| CC + GG | 41 | 7 (63.6) | 34 (87.2) | 3.88 (0.82-18.23) | 0.08 |
| GC or GG + GA or AA | 9 | 4 (36.4) | 5 (12.8) | Reference |  |
| CC or GC + GG or GA | 3 | 0 (0.0) | 3 (6.0) | NE | NE |
| GG + AA | 59 | 12 (100.0) | 47 (94.0) |  |  |
| ***MSH2* c.211+9C>G *+ TP53* c.215G>C** |  |  |  |  |  |
| CC + CC | 62 | 13 (100.0) | 49 (96.1) |  |  |
| CG or GG + GC or GG | 2 | 0 (0.0) | 2 (3.9) | NE | NE |
| CC or CG + CC or GC | 6 | 1 (12.5) | 5 (15.6) | 1.29 (0.13-12.96) | 0.82 |
| GG + GG | 34 | 7 (87.5) | 27 (84.4) | Reference |  |
| ***MSH2* c.211+9C>G *+ CASP3* c.-1191A>G** |  |  |  |  |  |
| CC + GG | 64 | 14 (87.5) | 50 (96.2) | 1.10 (0.24-4.86) | 0.90 |
| CG or GG + AG or AA | 4 | 2 (12.5) | 2 (3.8) | Reference |  |
| CC or CG + GG or AG | 12 | 3 (23.1) | 9 (21.4) | Reference | 0.61 |
| GG + AA | 43 | 10 (76.9) | 33 (78.6) | 1.61 (0.25-10.22) |  |
| ***MSH2* c.211+9C>G *+ CASP3* c.-182-247G>T** |  |  |  |  |  |
| CC + TT | 60 | 12 (100.0) | 48 (96.0) |  |  |
| CG or GG + GT or TT | 2 | 0 (0.0) | 2 (4.0) | NE | NE |
| CC or CG + TT or GT | 8 | 3 (21.4) | 5 (14.3) | Reference | 0.47 |
| GG + GG | 41 | 11 (78.6) | 30 (85.7) | 1.77 (0.37-8.55) |  |
| ***MSH2* c.211+9C>G *+ FAS* c.-1378G>A** |  |  |  |  |  |
| CC + AA | 66 | 12 (92.3) | 54 (96.4) | 2.25 (0.18-26.88) | 0.52 |
| CG or GG + GA or GG | 3 | 1 (7.7) | 2 (3.6) | Reference |  |
| CC or CG + AA or GA | 16 | 3 (30.0) | 13 (46.4) | 2.02 (0.43-9.46) | 0.37 |
| GG + GG | 22 | 7 (70.0) | 15 (53.6) | Reference |  |
| ***MSH2* c.211+9C>G *+ FAS* c.-671A>G** |  |  |  |  |  |
| CC + GG | 50 | 10 (90.9) | 40 (97.6) | 4.00 (0.23-69.64) | 0.34 |
| CG or GG + AG or GG | 2 | 1 (9.1) | 1 (2.4) | Reference |  |
| CC or CG + AG or GG | 6 | 1 (10.0) | 5 (11.6) | 1.18 (0.12-11.42) | 0.88 |
| GG + AA | 47 | 9 (90.9) | 38 (88.4) | Reference |  |
| ***MSH2* c.211+9C>G *+ FASL* c.-844C>T** |  |  |  |  |  |
| CC + TT | 54 | 11 (84.6) | 43 (89.6) | 1.56 (0.26-9.16) | 0.62 |
| GC or GG + CT or TT | 7 | 2 (15.4) | 5 (10.4) | Reference |  |
| CC or GC + TT or CT | 4 | 1 (7.7) | 3 (8.8) | 1.16 (0.11-1229) | 0.90 |
| GG + CC | 43 | 12 (92.3) | 31 (91.2) | Reference |  |
| ***MSH3* c.3133G>A *+ EXO1* c.1762G>A** |  |  |  |  |  |
| GG + GG | 47 | 9 (100.0) | 38 (95.0) |  |  |
| AG or AA + GA or AA | 2 | 0 (0.0) | 2 (5.0) | NE | NE |
| GG or AG + GG or GA | 7 | 3 (25.0) | 4 (14.3) | Reference | 0.41 |
| AA + AA | 33 | 9 (75.0) | 24 (85.7) | 2.00 (0.37-10.74) |  |
| ***MSH3* c.3133G>A *+ TP53* c.215G>C** |  |  |  |  |  |
| GG + CC |  | NE | NE | NE | NE |
| GA or AA + GC or GG |  |  |  |  |  |
| GG or GA + CC or GC | 21 | 5 (50.0) | 16 (53.3) | 1.14 (0.27-4.78) | 0.85 |
| AA + GG | 19 | 5 (50.0) | 14 (46.7) | Reference |  |
| ***MSH3* c.3133G>A *+ CASP3* c.-1191A>G** |  |  |  |  |  |
| GG + GG |  | NE | NE | NE | NE |
| GA or AA + AG or AA |  |  |  |  |  |
| GG or AG + GG or AG | 21 | 6 (54.5) | 16 (50.0) | Reference | 0.79 |
| AA + AA | 22 | 5 (45.5) | 16 (50.0) | 1.20 (0.30-4.74) |  |
| ***MSH3* c.3133G>A *+ CASP3* c.-182-247G>T** |  |  |  |  |  |
| GG + TT | 72 | 18 (100.0) | 54 (98.2) |  |  |
| GA or AA + GT or GG | 1 | 0 (0.0) | 1 (1.8) | NE | NE |
| GG or AG + TT or GT | 22 | 6 (42.9) | 16 (48.5) | 1.13 (0.32-3.98) | 0.84 |
| AA + GG | 25 | 8 (57.1) | 17 (51.5) | Reference |  |
| ***MSH3* c.3133G>A *+ FAS* c.-1378G>A** |  |  |  |  |  |
| GG +AA | 77 | 18 (94.7) | 59 (100.0) | NE | NE |
| GA or AA + GA or GG | 1 | 1 (5.3) | 0 (0.0) |  |  |
| GG or GA + AA or GA | 35 | 5 (62.5) | 30 (78.9) | 2.25 (0.44-11.48) | 0.33 |
| AA +GG | 11 | 3 (37.5) | 8 (21.1) | Reference |  |
| ***MSH3* c.3133G>A *+ FAS* c.-671A>G** |  |  |  |  |  |
| GG + GG | 62 | 15 (100.0) | 47 (97.9) |  |  |
| GA or AA + AG or AA | 1 | 0 (0.0) | 1 (2.1) | NE | NE |
| GG or GA + GG or AG | 12 | 2 (33.3) | 10 (34.5) | 1.05 (0.16-6.77) | 0.95 |
| AA + AA | 23 | 4 (66.7) | 19 (65.5) | Reference |  |
| ***MSH3* c.3133G>A *+ FASL* c.-844C>T** |  |  |  |  |  |
| GG + TT | 61 | 15 (100.0) | 46 (97.9) |  |  |
| GA or AA + CT or CC | 1 | 0 (0.0) | 1 (2.1) | NE | NE |
| GG or GA + TT or CT | 20 | 4 (30.8) | 16 (44.4) | 1.80 (0.46-6.93) | 0.39 |
| AA + CC | 29 | 9 (69.2) | 20 (55.6) | Reference |  |
| ***EXO1* c.1762G>A *+ TP53* c.215G>C** |  |  |  |  |  |
| GG + CC | 45 | 10 (90.9) | 35 (94.6) | 1.75 (0.14-21.34) | 0.66 |
| GA or AA + GC or GG | 3 | 1 (9.1) | 2 (5.4) | Reference |  |
| GG or AG + CC or GC | 5 | 3 (20.0) | 2 (6.1) | Reference | 0.16 |
| AA + GG | 43 | 12 (80.0) | 31 (93.9) | 3.87 (0.57-26.14) |  |
| ***EXO1* c.1762G>A *+ CASP3* c.-1191A>G** |  |  |  |  |  |
| GG + GG | 47 | 9 (90.0) | 38 (90.5) | 1.05 (0.10-10.61) | 0.96 |
| GA or AA + AG or AA | 5 | 1 (10.0) | 4 (9.5) | Reference |  |
| GG or AG + GG or AG | 6 | 1 (8.3) | 5 (12.2) | 1.52 (0.16-14.50) | 0.71 |
| AA + AA | 47 | 11 (91.7) | 36 (87.8) | Reference |  |
| ***EXO1* c.1762G>A *+ CASP3* c.-182-247G>T** |  |  |  |  |  |
| GG + TT | 43 | 2 (16.7) | 1 (2.9) | Reference | 0.13 |
| AG or AA + GT or GG | 3 | 10 (83.3) | 33 (97.1) | 6.60 (0.54-80.61) |  |
| GG or AG + TT or GT | 5 | 2 (13.3) | 3 (7.9) | Reference | 0.21 |
| AA + GG | 48 | 13 (86.7) | 35 (92.1) | 3.00 (0.53-16.89) |  |
| ***EXO1* c.1765G>A *+ FAS* c.-1378G>A** |  |  |  |  |  |
| GG + AA | 49 | 9 (81.8) | 40 (95.2) | 4.44 (0.55-35.90) | 0.16 |
| GA or AA + GA or GG | 4 | 2 (18.2) | 2 (4.8) | Reference |  |
| GG or GA + AA or GA | 9 | 1 (11.1) | 8 (32.0) | 3.76 (0.40-35.44) | 0.24 |
| AA + GG | 25 | 8 (88.9) | 17 (68.0) | Reference |  |
| ***EXO1* c.1762G>A *+ FAS* c.-671A>G** |  |  |  |  |  |
| GG + GG | 42 | 7 (77.8) | 35 (77.8) | 1.00 (0.17-5.59) | 1.00 |
| GA or AA + AG or AA | 12 | 2 (22.2) | 10 (22.2) | Reference |  |
| GG or GA + GG or AG | 1 | 0 (0.0) | 1 (2.4) | NE | NE |
| AA + AA | 52 | 11 (100.0) | 41 (97.6) |  |  |
| ***EXO1* c.1765G>A *+ FASL* c.-844C>T** |  |  |  |  |  |
| GG + TT | 40 | 8 (72.7) | 32 (80.0) | 1.50 (0.32-6.97) | 0.60 |
| AG or AA + CT or CC | 11 | 3 (27.3) | 8 (20.0) | Reference |  |
| GG or GA + TT or CT | 4 | 1 (6.3) | 3 (7.3) | 1.18 (0.11-12.30) | 0.88 |
| AA + CC | 53 | 15 (93.8) | 38 (92.7) | Reference |  |
| ***TP53* c.215G>C *+ CASP3* c.-182-247G>T** |  |  |  |  |  |
| CC + TT | 70 | 18 (100.0) | 52 (96.3) |  |  |
| GC or GG + GT or GG | 2 | 0 (0.0) | 2 (3.7) | NE | NE |
| CC or GC + TT or GT | 15 | 3 (27.3) | 12 (35.3) | 1.14 (0.27-4.75) | 0.85 |
| GG + GG | 30 | 8 (72.7.4) | 22 (64.7) | Reference |  |
| ***TP53* c.215G>C *+ FAS* c.-1378G>A** |  |  |  |  |  |
| CC + AA |  | NE | NE | NE | NE |
| GC or GG + GA or GG |  |  |  |  |  |
| CC or GC + AA or GA | 27 | 6 (54.5) | 9 (29.0) | Reference | 0.13 |
| GG + GG | 15 | 5 (45.5) | 22 (71.0) | 2.93 (0.71-12.10) |  |
| ***TP53* c.215G>C *+ FAS* c.-671A>G** |  |  |  |  |  |
| CC + GG | 59 | 16 (94.1) | 43 (100.0) | NE | NE |
| GC or GG + AG or AA | 1 | 1 (5.9) | 0 (0.0) |  |  |
| CC or GC + AG or GG | 13 | 3 (27.3) | 10 (26.3) | Reference | 0.95 |
| GG + AA | 36 | 8 (72.7) | 28 (73.7) | 1.05 (0.23-4.75) |  |
| ***TP53* c.215G>C *+ FASL* c.-844C>T** |  |  |  |  |  |
| CC + TT | 63 | 16 (94.1) | 47 (90.4) | Reference | 0.63 |
| GC or GG + CT or CT | 6 | 1 (5.9) | 5 (9.6) | 1.70 (0.18-15.68) |  |
| CC or GC + TT or CT | 11 | 1 (10.0) | 10 (30.3) | 3.91 (0.43-35.15) | 0.22 |
| GG + CC | 32 | 9 (90.0) | 23 (69.7) | Reference |  |
| ***CASP3* c.-1191A>G *+ CASP3* c.-182-247G>T** |  |  |  |  |  |
| GG + TT |  | NE | NE | NE | NE |
| AG or AA + GT or GG |  |  |  |  |  |
| GG or AG + TT or GT | 9 | 1 (12.5) | 8 (28.6) | 2.80 (0.29-26.56) | 0.50 |
| AA + GG | 27 | 7 (87.5) | 20 (71.4) | Reference |  |
| ***CASP3* c.-1191A>G *+ FAS* c.-1378G>A** |  |  |  |  |  |
| GG +AA |  | NE | NE | NE | NE |
| AG or AA + GA or GG |  |  |  |  |  |
| GG or AG + AA or GA | 25 | 4 (40.0) | 21 (67.7) | 3.15 (0.72-13.73) | 0.12 |
| AA + GG | 16 | 6 (60.0) | 10 (32.3) | Reference |  |
| ***CASP3* c.-1191A>G *+ FAS* c.-671A>G** |  |  |  |  |  |
| GG + GG | 59 | 14 (100.0) | 45 (97.8) |  |  |
| AG or AA + AG or AA | 1 | 0 (0.0) | 1 (2.2) | NE | NE |
| GG or AG + GG or AG | 11 | 3 (25.0) | 8 (22.2) | Reference | 0.84 |
| AA + AA | 37 | 9 (75.0) | 28 (77.8) | 1.16 (0.25-5.35) |  |
| ***CASP3* c.-1191A>G *+ FASL* c.-844C>T** |  |  |  |  |  |
| GG + TT | 61 | 15 (93.8) | 46 (93.9) | 1.02 (0.09-10.58) | 0.98 |
| AG or AA + CT or CC | 4 | 1 (6.3) | 3 (6.1) | Reference |  |
| GG or AG + TT or CT | 10 | 1 (9.1) | 9 (27.3) | 3.75 (0.41-33.63) | 0.23 |
| AA + CC | 34 | 10 (90.9) | 24 (72.7) | Reference |  |
| ***CASP3* c.-182-247G>T *+ FAS* c.-671A>G** |  |  |  |  |  |
| TT or GG | 57 | 14 (100.0) | 43 (97.7) |  |  |
| GT or GG + AG or AA | 1 | 0 (0.0) | 1 (2.3) | NE | NE |
| TT or GT + GG or AG | 8 | 1 (11.1) | 7 (20.0) | 2.00 (0.21-18.74) | 0.83 |
| GG + AA | 36 | 8 (88.9) | 28 (80.0) | Reference |  |
| ***CASP3* c.-182-247G>T *+ FASL* c.-844C>T** |  |  |  |  |  |
| TT + TT | 57 | 14 (100.0) | 43 (95.6) |  |  |
| GT or GG + CT or CC | 2 | 0 (0.0) | 2 (4.4) | NE | NE |
| TT or GT + TT or CT | 10 | 3 (18.8) | 7 (23.3) | 1.04 (0.21-4.98) | 0.95 |
| GG + CC | 36 | 13 (81.3) | 23 (76.7) | Reference |  |
| ***FAS* c.-671A>G + *FASL* c.-844C>T** |  |  |  |  |  |
| GG + TT | 51 | 13 (86.7) | 38 (90.5) | 1.46 (0.23-8.93) | 0.22 |
| AG or AA + CT or CC | 6 | 2 (13.3) | 4 (9.5) | Reference |  |
| GG or AG + TT or CT | 7 | 1 (8.3) | 6 (16.7) | 2.20 (0.23-20.39) | 0.48 |
| AA + CC | 41 | 11 (91.7) | 30 (83.3) | Reference |  |

N: number of patients. CR: complete response. PR: partial response. SD: stable disease. OR: odds ratio. CI: confidence interval. NE: not evaluated. Results with significant *P-*values are presented in bold letters. Variables with *P* values < 0.20 and those with more than 10 individuals in each group were included in the multivariate analysis
