# Supplementary material for: Genetic variability in cisplatin metabolic pathways and outcome of locally advanced head and neck squamous cell carcinoma patients
Source: Sci Rep. 2023 Oct 5;13:16762. doi: 10.1038/s41598-023-44040-7 (PMC10556039; doi:10.1038/s41598-023-44040-7)
Supplement: Supplementary file 5 — Supplementary Table S5. [file 41598_2023_44040_MOESM5_ESM.docx]

**Supplementary information 5**

Genetic variability in cisplatin metabolic pathways and outcome of locally advanced head and neck squamous cell carcinoma patients

Ana Maria Castro Ferreira^1^, João Maurício Carrasco Altemani^2^, Ligia Traldi Macedo^1^, Gustavo Jacob Lourenço^1^, Carmen Silvia Passos Lima^1,2^*

Corresponding author

^*^Carmen S. P. Lima, MD, PhD

Clinical Oncology Service

Department of Anesthesiology, Oncology and Radiology

Faculty of Medical Sciences

University of Campinas

Rua Alexander Fleming, 181

Cidade Universitária “Zeferino Vaz”

Barão Geraldo, Campinas, São Paulo, Brazil

CEP: 13083-970

Phone and fax simile: +55 19 3521 9120

E-mail: [carmenl@fcm.unicamp.br](mailto:carmenl@fcm.unicamp.br)

**Supplementary table S5**. Clinicopathological aspects, and genotypes of detoxification, DNA-repair, and apoptosis-related single nucleotide variants and survival of 109 patients with head and neck squamous cell carcinoma

| **Variable** | **Event-free survival** | | |  | **Overall survival** | | |
| --- | --- | --- | --- | --- | --- | --- | --- |
|  | **Univariate Cox analysis** | | |  | **Univariate Cox analysis** | | |
|  | **N of event/ N total** | ***P-*value** | **HR (95% CI)** |  | **N of event/ N total** | ***P-*value** | **HR (95% CI)** |
| **Median age** |  |  |  |  |  |  |  |
| ≤ 56 years | 44/57 |  | Reference |  | 46/57 | 0.92 | Reference |
| > 56 years | 39/52 | 0.47 | 0.85 (0.55-1.31) |  | 43/52 |  | 0.98 (0.64-1.48) |
| **Tumor size** |  |  |  |  |  |  |  |
| T1 or T2 | 16/27 | **0.003** | Reference |  | 18/27 | **0.001** | Reference |
| T3 or T4 | 67/82 |  | **2.33 (1.34-4.06)** |  | 71/82 |  | **3.39 (1.41-4.05)** |
| **Nodal status** |  |  |  |  |  |  |  |
| N1 or N2 | 28/42 | 0.08 | Reference |  | 31/42 | 0.08 | Reference |
| N3 or N4 | 55/67 |  | 1.49 (0.94-2.36) |  | 58/67 |  | 1.47 (0.95-2.28) |
| **Tumor stage** |  |  |  |  |  |  |  |
| I or II | 2/6 | **0.03** | Reference |  | 2/6 | **0.02** | Reference |
| III or IV | 81/103 |  | **4.50 (1.09-18.53)** |  | 87/103 |  | **5.12 (1.25-21.03)** |
| **Average time to treatment delivery (days)** |  |  |  |  |  |  |  |
| ≤ 123 | 49/65 | 0.99 | 1.00 (0.64-1.56) |  | 54/65 | 0.66 | 1.10 (0.71-1.70) |
| > 123 | 32/42 |  | Reference |  | 33/42 |  | Reference |
| ***GSTM1*** | | | | | | | |
| Present | 33/48 | 0.21 | Reference |  | 36/48 | 0.22 | Reference |
| Null | 50/61 |  | 1.42 (0.91-2.21) |  | 53/61 |  | 1.39 (0.91-2.13) |
| ***GSTT1*** |  |  |  |  |  |  |  |
| Present | 70/92 | 0.65 | 1.14 (0.63-2.06) |  | 75/92 | 0.64 | 1.14 (0.64-2.03) |
| Null | 13/17 |  | Reference |  | 14/17 |  | Reference |
| ***GSTP1* c.313A>G** |  |  |  |  |  |  |  |
| AA | 40/54 | 0.82 | Reference |  | 44/54 | 0.55 | 1.13 (0.74-1.71) |
| AG or GG | 43/55 |  | 1.50 (0.68-1.61) |  | 45/55 |  | Reference |
| AA or AG | 77/103 | **0.02** | **2.69 (1.13-6.38)** |  | 85/103 | 0.68 | 1.23 (0.45-3.36) |
| GG | 6/6 |  | Reference |  | 4/6 |  | Reference |
| ***XPC* c.2815A>C** |  |  |  |  |  |  |  |
| AA | 27/41 | 0.25 | Reference |  | 31/41 | 0.47 | Reference |
| AC or CC | 56/68 |  | 1.30 (0.73-1.95) |  | 58/68 |  | 1.17 (0.75-181) |
| AA or AC | 72/96 | 0.42 | 1.29 (0.68-2.44) |  | 77/96 | 0.61 | 1.17 (0.63-2.15) |
| CC | 11/13 |  | Reference |  | 12/13 |  | Reference |
| ***XPD* c.934G>A** |  |  |  |  |  |  |  |
| GG | 42/59 | 0.18 | Reference |  | 45/59 | 0.15 | Reference |
| GA or AA | 41/50 |  | 1.34 (0.87-2.07) |  | 44/50 |  | 1.35 (0.89-2.06) |
| GG or GA | 73/98 |  | Reference |  | 78/98 |  | Reference |
| AA | 10/11 | **0.01** | **2.37 (1.20 (4.65)** |  | 11/11 | **0.005** | **2.51 (1.31-4.80)** |
| ***XPD* c.2251A>C** |  |  |  |  |  |  |  |
| AA | 38/55 | 0.10 | Reference |  | 41/55 | 0.14 | Reference |
| AC or CC | 45/54 |  | 1.44 (0.93-2.22) |  | 48/54 |  | 1.36 (0.89-2.07) |
| AA or AC | 76/100 |  | Reference |  | 80/100 |  | Reference |
| CC | 7/9 | 0.95 | 1.02 (0.47-2.22) |  | 9/9 | 0.52 | 1.25 (0.62-2.50) |
| ***XPF* c.2505T>C** |  |  |  |  |  |  |  |
| TT | 37/52 | 0.22 | 1.30 (0.84-2.02) |  | 42/52 | 0.33 | Reference |
| TC or CC | 46/57 |  | Reference |  | 47/57 |  | 1.23 (0.80-1.87) |
| TT or TC | 78/101 | 0.69 | 1.19 (0.48-2.97) |  | 83/101 | 0.97 | 1.01 (0.44-2.32) |
| CC | 5/8 |  | Reference |  | 6/8 |  | Reference |
| ***ERCC1* c.354C>T** |  |  |  |  |  |  |  |
| CC | 24/28 | 0.13 | 1.43 (0.89-2.31) |  | 25/28 | 0.19 | 1.36 (0.85-2.16) |
| CT or TT | 59/81 |  | Reference |  | 64/81 |  | Reference |
| CC or CT | 71/89 | **0.04** | **1.89 (1.02-3.50)** |  | 76/89 | **0.03** | **1.92 (1.06-3.47)** |
| TT | 12/20 |  | Reference |  | 13/20 |  | Reference |
| ***MLH1* c.93G>A** |  |  |  |  |  |  |  |
| GG | 49/63 | 0.84 | 1.04 (0.67-1.62) |  | 50/63 | 0.55 | Reference |
| GA or AA | 34/46 |  | Reference |  | 39/46 |  | 1.13 (0.74-1.72) |
| GG or GA | 80/105 | 0.87 | 1.09 (0.34-3.48) |  | 86/105 | 0.63 | 1.31 (0.41-4.18) |
| AA | 3/4 |  | Reference |  | 3/4 |  |  |
| ***MSH2* c.211+9C>G** |  |  |  |  |  |  |  |
| CC | 19/25 | 0.95 | Reference |  | 70/84 | 0.70 | Reference |
| CG or GG | 64/84 |  | 1.01 (0.60-1.69) |  | 19/25 |  | 1.10 (0.66-1.83) |
| CC or CG | 61/82 | 0.82 | Reference |  | 67/82 | 0.83 | Reference |
| GG | 22/27 |  | 1.05 (0.64-1.72) |  | 22/27 |  | 1.05 (0.64-1.70) |
| ***MSH3* c.3133G>A** |  |  |  |  |  |  |  |
| GG | 8/10 | 0.71 | Reference |  | 6/10 | 0.23 | Reference |
| GA or AA | 75/99 |  | 1.14 (0.55-2.38) |  | 83/99 |  | 1.65 (0.72-3.79) |
| GG or GA | 36/48 | 0.79 | 1.06 (0.68-1.64) |  | 40/48 | 0.57 | 1.12 (0.74-1.71) |
| AA | 47/61 |  | Reference |  | 49/61 |  | Reference |
| ***EXO1* c.1765G>A** |  |  |  |  |  |  |  |
| GG | 37/45 | 0.08 | 1.46 (0.94-2.26) |  | 39/45 | 0.20 | 1.31 (0.86-2.00) |
| GA or AA | 46/64 |  | Reference |  | 50/64 |  | Reference |
| GG or GA | 75/98 | 0.55 | 1.24 (0.59-2.58) |  | 79/98 | 0.82 | 1.07 (0.55-2.08) |
| AA | 8/11 |  | Reference |  | 10/11 |  | Reference |
| ***TP53* c.215G>C** |  |  |  |  |  |  |  |
| CC | 8/11 | 0.70 | Reference |  | 8/11 | 0.76 | 1.11 (0.54-3.31) |
| GG or GC | 75/98 |  | 1.14 (0.55-2.38) |  | 81/98 |  | Reference |
| CC or GC | 43/58 | 0.83 | Reference |  | 47/58 |  | 1.03 (0.68-1.56) |
| GG | 40/51 |  | 1.04 (0.67-1.61) |  | 42/51 | 0.87 | Reference |
| ***CASP3* c.-1191A>G** |  |  |  |  |  |  |  |
| GG | 6/11 | 0.18 | Reference |  | 8/11 |  | Reference |
| AA or AG | 77/98 |  | 1.75 (0.76-4.02) |  | 81/98 | 0.51 | 1.27 (0.61-2.63) |
| AG or GG | 46/64 | 0.27 | Reference |  | 51/64 |  | Reference |
| AA | 37/45 |  | 1.27 (0.82-1.96) |  | 38/45 | 0.22 | 1.29 (0.85-1.97) |
| ***CASP3* c.-182-247G>T** |  |  |  |  |  |  |  |
| TT | 27/39 | 0.61 | Reference |  | 34/39 | 0.76 | 1.06 (0.69-1.64) |
| GT or GG | 56/70 |  | 0.88 (0.56-1.41) |  | 55/70 |  | Reference |
| TT or GT | 72/96 | 0.88 | Reference |  | 78/96 |  | Reference |
| GG | 11/13 |  | 1.05 (0.55-1.98) |  | 11/13 | 0.92 | 1.03 (0.54-1.94) |
| ***FAS*** **c.-1378G>A** |  |  |  |  |  |  |  |
| AA | 5/7 | 0.78 | Reference |  | 5/7 |  | Reference |
| GA or GG | 78/102 |  | 1.13 (0.46-2.81) |  | 84/102 | 0.55 | 1.31 (0.53-3.24) |
| AA or GA | 23/29 | 0.78 | Reference |  | 24/29 | 0.66 | Reference |
| GG | 60/80 |  | 1.07 (0.66-1.73) |  | 65/80 |  | 1.10 (0.69-1.77) |
| ***FAS* c.-671A>G** |  |  |  |  |  |  |  |
| GG | 22/26 | 0.09 | 1.52 (0.93-2.48) |  | 66/83 | 0.08 | 1.51 (0.94-2.44) |
| AG or AA | 61/83 |  | Reference |  | 23/26 |  | Reference |
| GG or AG | 59/75 | 0.44 | Reference |  | 65/75 | 0.15 | 1.40 (0.88-2.25) |
| AA | 24/34 |  | 1.20 (0.74-1.93) |  | 24/34 |  | Reference |
| ***FASL* c.-844C>T** |  |  |  |  |  |  |  |
| TT | 21/27 | 0.48 | 1.19 (0.72-1.96) |  | 21/27 | 0.74 | 1.08 (0.66-1.77) |
| CT or CC | 62/82 |  | Reference |  | 68/82 |  | Reference |
| TT or CT | 60/77 | 0.56 | 1.15 (0.71-1.86) |  | 62/77 |  | Reference |
| CC | 23/32 |  | Reference |  | 27/32 | 0.85 | 1.04 (0.66-1.64) |
| ***GSTM1 + GSTT1*** |  |  |  |  |  |  |  |
| Present + Present | 8/10 | 0.64 | 1.20 (0.54-2.65) |  | 8/10 | 0.71 | 1.15 (0.52-2.53) |
| Null + Null | 28/41 |  | Reference |  | 30/41 |  | Reference |
| ***GSTM1* + *GSTP1* c.313A>G** |  |  |  |  |  |  |  |
| Present + AA | 8/10 | 0.64 | 1.20 (0.54-2.65) |  | 8/10 | 0.71 | 1.15 (0.52-2.53) |
| Null + AG or GG | 28/41 |  | Reference |  | 30/41 |  | Reference |
| Present + AA or AG | 2/2 | 0.13 | 3.07 (0.69-13.48) |  | 1/ 2 | 0.79 | 1.29 (0.17-9.53) |
| Null + GG | 29/44 |  | Reference |  | 33/44 |  | Reference |
| ***GSTM1 + XPC* c.2815A>C** |  |  |  |  |  |  |  |
| Present + AA | 33/40 | **0.03** | **2.19 (1.07-4.48)** |  | 34/40 | **0.05** | **1.89 (0.97-3.68)** |
| Null + AC or CC | 10/20 |  | Reference |  | 12/20 |  | Reference |
| Present + AA or AC | 6/6 | 0.06 | 2.29 (0.93-5.63) |  | 6/6 | 0.20 | 1.79 (0.73-4.36) |
| Null + CC | 28/41 |  | Reference |  | 30/41 |  | Reference |
| ***GSTM1 + XPD* c.934G>A** |  |  |  |  |  |  |  |
| Present + GG | 24/27 | **0.03** | **2.06 (1.07-3.96)** |  | 26/27 | **0.02** | **1.99 (1.07-3.69)** |
| Null + GA or AA | 16/25 |  | Reference |  | 18/25 |  | Reference |
| Present + GG or GA | 6/6 | **0.009** | **3.47 (1.36-8.82)** |  | 6/6 | **0.01** | 3.19 (1.26-8.07) |
| Null + AA | 29/43 |  | Reference |  | 31/43 |  | Reference |
| ***GSTM1 +XPD* c.2251A>C** |  |  |  |  |  |  |  |
| Present + AA | 26/30 | **0.02** | **2.17 (1.10-4.25)** |  | 28/31 | **0.04** | **1.93 (1.02-3.62)** |
| Null + AC or CC | 14/24 |  | Reference |  | 16/24 |  | Reference |
| Present + AA or AC | 3/ 4 | 0.79 | 1.17 (0.35-3.87) |  | 4/4 | 0.49 | 1.44 (0.50-4.11) |
| Null + CC | 29/43 |  | Reference |  | 31/43 |  | Reference |
| ***GSTM1 +XPF* c.2505T>C** |  |  |  |  |  |  |  |
| Present + TT | 26/31 | **0.04** | **2.00 (1.01-3.92)** |  | 26/31 | 0.08 | 1.75 (0.91-3.35) |
| Null + TC or CC | 22/31 |  | Reference |  | 15/22 |  | Reference |
| Present + TT or TC | 3/ 4 | 0.44 | 1.59 (0.48-5.27) |  | 3/ 4 | 0.35 | 1.75 (0.53-5.81) |
| Null + CC | 31/44 |  | Reference |  | 33/44 |  | Reference |
| ***GSTM1 + ERCC1* c.354C>T** |  |  |  |  |  |  |  |
| Present + CC | 38/47 | 0.85 | Reference |  | 40/47 |  | Reference |
| Null + CT or TT | 12/14 |  | 1.06 (0.55-2.03) |  | 12/14 | 0.89 | 1.04 (0.54-1.99) |
| Present + CC or CT | 9/12 | 0.52 | Reference |  | 9/12 |  | Reference |
| Null + TT | 30/40 |  | 1.27 (0.60-2.69) |  | 32/40 | 0.31 | 1.46 (0.69-3.08) |
| ***GSTM1 + MLH1* c.93G>A** |  |  |  |  |  |  |  |
| Present + GG | 21/29 | 0.25 | 1.43 (0.77-2.66) |  | 25/29 | 0.15 | 1.52 (0.85-2.73) |
| Null + GA or AA | 20/31 |  | Reference |  | 22/31 |  | Reference |
| Present + GG or GA | 3/3 | 0.44 | 1.58 (0.48-5.19) |  | 3/3 | 0.64 | 1.32 (0.40-4.31) |
| Null + AA | 33/47 |  | Reference |  | 36/47 |  | Reference |
| ***GSTM1 + MSH2* c.211+9C>G** |  |  |  |  |  |  |  |
| Present + CC | 36/49 | 0.51 | 1.27 (0.61-2.63) |  | 43/49 | 0.27 | 1.48 (0.72-3.05) |
| Null + CG or GG | 9/13 |  | Reference |  | 9/13 |  | Reference |
| Present + CC or CG | 14/16 | 0.39 | 1.47 (0.75-2.88) |  | 14/16 | 0.34 | 1.36 (0.71-2.61) |
| Null + GG | 25/37 |  | Reference |  | 28/37 |  | Reference |
| ***GSTM1 + MSH3* c.3133G>A** |  |  |  |  |  |  |  |
| Present + GG | 45/55 | 0.44 | 1.58 (0.49-5.14) |  | 48/55 | 0.06 | 6.45 (0.88-46.92) |
| Null + GA or AA | 3/4 |  | Reference |  | 1/4 |  | Reference |
| Present + GG or GA | 26/33 | 0.25 | 1.49 (0.75-2.97) |  | 27/33 | 0.39 | 1.32 (0.69-2.53) |
| Null + AA | 12/20 |  | Reference |  | 14/20 |  | Reference |
| ***GSTM1 + EXO1* c.1762G>A** |  |  |  |  |  |  |  |
| Present + GG | 26/33 | 0.98 | 1.00 (0.51-1.96) |  | 26/33 | 0.69 | 1.14 (0.57-2.27) |
| Null + GA or AA | 13/17 |  | Reference |  | 12/17 |  | Reference |
| Present + GG or GA | 5/5 | 0.20 | 1.87 (0.71-4.91) |  | 5/5 | 0.12 | 2.14 (0.81-5.65) |
| Null + AA | 30/42 |  | Reference |  | 31/42 |  | Reference |
| ***GSTM1 + TP53* c.215G>C** |  |  |  |  |  |  |  |
| Present + CC | 46/55 | 0.44 | 1.49 (0.53-4.16) |  | 48/55 | 0.30 | 1.85 (0.57-5.97) |
| Null + GG or GC | 4/5 |  | Reference |  | 3/5 |  | Reference |
| Present + CC or GC | 24/30 | 0.19 | 1.51 (0.81-2.82) |  | 25/30 | 0.31 | 1.35 (0.74-2.46) |
| Null + GG | 17/27 |  | Reference |  | 19/27 |  | Reference |
| ***GSTM1 + CASP3* c.-1191A>G** |  |  |  |  |  |  |  |
| Present + GG | 48/56 | 0.46 | 1.46 (0.52-4.01) |  | 49/56 | 0.49 | 1.42 (0.51-3.96) |
| Null + AA or AG | 4/6 |  | Reference |  | 4/6 |  | Reference |
| Present + GG or AG | 20/22 | **0.04** | **1.95 (1.00-3.79)** |  | 20/22 | **0.04** | **1.96 (1.03-3.74)** |
| Null + AA | 16/25 |  | Reference |  | 18/25 |  | Reference |
| ***GSTM1 + CASP3* c.-182-247G>T** |  |  |  |  |  |  |  |
| Present + TT | 40/50 | 0.48 | 2.04 (0.27-14.89) |  | 43/50 | 0.44 | 2.17 (0.29-15.88) |
| Null + GT or GG | 1/2 |  | Reference |  | 1/2 |  | Reference |
| Present + TT or GT | 15/20 | 0.43 | 1.30 (0.66-2.55) |  | 18/20 | 0.19 | 1.52 (0.80-2.91) |
| Null + GG | 21/29 |  | Reference |  | 20/29 |  | Reference |
| ***GSTM1 + FAS* c.-1378G>A** |  |  |  |  |  |  |  |
| Present + AA | 48/59 | 0.33 | 1.78 (0.55-5.77) |  | 51/59 | 0.19 | 2.19 (0.67-7.06) |
| Null + GA or GG | 3/5 |  | Reference |  | 3/5 |  | Reference |
| Present + AA or GA | 35/42 | 0.33 | 1.50 (0.66-3.39) |  | 37/42 | 0.19 | 1.70 (0.75-3.83) |
| Null +GG | 8/10 |  | Reference |  | 8/10 |  | Reference |
| ***GSTM1 + FAS* c.-671A>G** |  |  |  |  |  |  |  |
| Present + GG | 37/45 |  | Reference |  | 39/45 |  | Reference |
| Null + AG or AA | 9/10 | 0.54 | 1.25 (0.60-4.61) |  | 9/10 | 0.55 | 1.24 (0.60-2.57) |
| Present + GG or AG | 3/10 | 0.58 | 1.23 (0.57-2.67) |  | 10/13 | 0.97 | 1.01 (0.47-2.14) |
| Null + AA | 19/27 |  | Reference |  | 22/27 |  | Reference |
| ***GSTM1 + FASL* c.-844C>T** |  |  |  |  |  |  |  |
| Present + TT | 39/47 | 0.70 | 1.14 (0.57-2.29) |  | 41/47 | 0.50 | 1.27 (0.61-2.63) |
| Null + CT or CC | 10/13 |  | Reference |  | 9/13 |  | Reference |
| Present + CT or TT | 17/20 | 0.45 | 1.26 (0.68-2.32) |  | 18/20 | 0.26 | 1.41 (0.77-2.58) |
| Null + CC | 27/36 |  | Reference |  | 27/36 |  | Reference |
| ***GSTTI + GSTP1* c.313A>G** |  |  |  |  |  |  |  |
| Present + AA | 7/8 |  | Reference |  | 7/8 |  | Reference |
| Null + AG or GG | 34/45 | 0.85 | 1.08 (0.47-2.45) |  | 37/45 | 0.71 | 1.16 (0.51-2.62) |
| Present + AA or AG |  |  | NE |  |  |  | NE |
| Null + GG |  |  |  |  |  |  |  |
| ***GSTTI + XPC* c.2815A>C** |  |  |  |  |  |  |  |
| Present + AA | 7/10 | 0.92 | 1.04 (0.44-2.46) |  | 8/10 | 0.76 | 1.13 (0.51-2.51) |
| Null + AC or CC | 21/34 |  | Reference |  | 25/34 |  | Reference |
| Present + AA or AC | 1/ 2 | 0.49 | 1.97 (0.27-14.31) |  | 2/2 | 0.99 | 1.00 (0.24-4.11) |
| Null + CC | 60/81 |  | Reference |  | 65/81 |  | Reference |
| ***GSTTI + XPD* c.934G>A** |  |  |  |  |  |  |  |
| Present + GG | 7/9 | 0.80 | 1.10 (0.49-2.49) |  | 8/9 | 0.78 | 1.11 (0.51-2.39) |
| Null + GA or AA | 36/51 |  | Reference |  | 39/51 |  | Reference |
| Present + GG or GA | 1/1 | 0.18 | 3.85 (0.51-28.77) |  | 1/1 | 0.68 | 1.51 (0.20-11.03) |
| Null + AA | 61/82 |  | Reference |  | 65/82 |  | Reference |
| ***GSTT1 + XPD* c.2251A>C** |  |  |  |  |  |  |  |
| Present + AA | 7/9 | 0.78 | 1.12 (0.49-2.55) |  | 8/9 | 0.89 | 1.05 (0.48-2.27) |
| Null + AC or CC | 32/47 |  | Reference |  | 35/47 |  | Reference |
| Present + AA or AC | 2/2 | 0.77 | 1.22 (0.29-5.02) |  | 2/2 | 0.90 | 1.08 (0.26-4.45) |
| Null + CC | 65/85 |  | Reference |  | 68/85 |  | Reference |
| ***GSTT1 + XPF* c.2505T>C** |  |  |  |  |  |  |  |
| Present + TT | 9/10 | 0.30 | 1.47 (0.69-3.14) |  | 9/10 | 0.31 | 1.46 (0.69-3.10) |
| Null + TC or CC | 33/45 |  | Reference |  | 37/45 |  | Reference |
| Present + TT or TC | 1/2 | 0.46 | 2.11 (0.28-15.51) |  | 1/ 2 | 0.42 | 2.25 (0.30-16.50) |
| Null + CC | 66/86 |  | Reference |  | 70/86 |  | Reference |
| ***GSTTI + ERCC1* c.354C>T** |  |  |  |  |  |  |  |
| Present + CC | 11/13 | 0.25 | Reference |  | 11/13 |  | Reference |
| Null + CT or TT | 22/24 |  | 1.52 (0.73-3.17) |  | 22/24 | 0.32 | 1.45 (0.69-3.04) |
| Present + CC or CT | 2/2 | 0.40 | 1.82 (0.44-7.55) |  | 2/2 | 0.27 | 2.21 (0.53-9.19) |
| Null + TT | 60/74 |  | Reference |  | 64/74 |  | Reference |
| ***GSTTI + MLH1* c.93G>A** |  |  |  |  |  |  |  |
| Present + GG | 6/8 | 0.92 | Reference |  | 6/8 |  | Reference |
| Null + GA or AA | 42/54 |  | 1.04 (0.44-2.46) |  | 42/54 | 0.84 | 1.09 (0.46-2.57) |
| Present + GG or GA |  |  | NE |  |  |  | NE |
| Null + AA |  |  |  |  |  |  |  |
| ***GSTTI + MSH2* c.211+9C>G** |  |  |  |  |  |  |  |
| Present + CC | 9/12 | 0.75 | 1.13 (0.49-2.60) |  | 10/12 | 0.98 | 1.00 (0.445-2.25) |
| Null + CG or GG | 15/20 |  | Reference |  | 15/20 |  | Reference |
| Present + CC or CG | 3/5 | 0.73 | 1.22 (0.38-3.93) |  | 4/5 | 0.99 | 1.00 (0.36-2.77) |
| Null + GG | 51/70 |  | Reference |  | 57/70 |  | Reference |
| ***GSTTI + MSH3* c.3133G>A** |  |  |  |  |  |  |  |
| Present + GG | 12/15 |  | Reference |  | 13/15 |  | Reference |
| Null + GA or AA | 7/8 | 0.26 | 1.71 (0.66-4.45) |  | 5/8 | 0.58 | 1.33 (0.46-3.81) |
| Present + GG or GA | 6/7 |  | Reference |  | 6/7 |  | Reference |
| Null + AA | 29/38 | 0.78 | 1.13 (0.46-2.75) |  | 32/38 | 0.74 | 1.15 (0.47-2.80) |
| ***GSTTI + EXO1* c.1762G>A** |  |  |  |  |  |  |  |
| Present + GG | 6/9 | 0.17 | Reference |  | 7/9 |  | Reference |
| Null + GA or AA | 30/37 |  | 1.84 (0.76-4.44) |  | 32/37 | 0.27 | 1.58 (0.69-3.62) |
| Present + GG or GA | 1/1 | 0.75 | 1.36 (0.18-9.95) |  | 1/1 | 0.60 | 1.69 (0.23-12.36) |
| Null + AA | 63/82 |  | Reference |  | 66/82 |  | Reference |
| ***GSTTI + TP53* c.215G>C** |  |  |  |  |  |  |  |
| Present + CC | 12/16 | 0.91 | 1.05 (0.41-2.67) |  | 13/16 | 0.93 | 1.03 (0.41-2.61) |
| Null + GC or GG | 7/10 |  | Reference |  | 7/10 |  | Reference |
| Present + CC or GC | 6/8 | 0.96 | Reference |  | 6/8 |  | Reference |
| Null + GG | 36/49 |  | 1.02 (0.42-2.43) |  | 39/49 | 0.83 | 1.09 (0.46-2.61) |
| ***GSTTI + CASP3* c.-1191A>G** |  |  |  |  |  |  |  |
| Present + GG | 13/16 | 0.51 | 1.38 (0.52-3.65) |  | 14/16 | 0.93 | 1.04 (0.42-2.52) |
| Null + AG or AA | 6/10 |  | Reference |  | 8/10 |  | Reference |
| Present + GG or AG | 7/7 | 0.33 | 1.49 (0.66-3.34) |  | 7/7 | 0.21 | 1.65 (0.74-3.70) |
| Null + AA | 40/54 |  | Reference |  | 44/54 |  | Reference |
| ***GSTTI + CASP3* c.-182-247G>T** |  |  |  |  |  |  |  |
| Present + TT | 10/14 |  | Reference |  | 11/14 |  | Reference |
| Null + GT or GG | 8/10 | 0.83 | 1.10 (0.43-2.80) |  | 8/10 | 0.90 | 1.05 (0.42-2.65) |
| Present + TT or GT | 2/5 |  | Reference |  | 3/5 |  | Reference |
| Null + GG | 45/58 | 0.14 | 2.86 (0.69-11.83) |  | 44/58 | 0.28 | 1.91 (0.59-6.16) |
| ***GSTTI + FAS* c.-1378G>A** |  |  |  |  |  |  |  |
| Present + AA | 11/15 | 0.87 | 1.11 (0.30-4.00) |  | 12/15 | 0.75 | 1.22 (0.34-44.37) |
| Null + GA or GG | 3/5 |  | Reference |  | 3/5 |  | Reference |
| Present + AA or GA | 9/12 |  | Reference |  | 10/12 |  | Reference |
| Null + GG | 19/24 | 0.99 | 1.00 (0.45-2.20) |  | 20/24 | 0.87 | 1.06 (0.49-2.27) |
| ***GSTTI + FAS* c.-671A>G** |  |  |  |  |  |  |  |
| Present + GG | 11/14 | 0.24 | Reference |  | 12/14 |  | Reference |
| Null + AG or AA | 20/23 |  | 1.55 (0.74-3.26) |  | 21/23 | 0.20 | 1.58 (0.77-3.24) |
| Present + GG or AG | 6/7 | 0.68 | 1.19 (0.50-2.78) |  | 7/7 | 0.48 | 1.32 (0.60-2.93) |
| Null + AA | 52/65 |  | Reference |  | 58/65 |  | Reference |
| ***GSTTI + FASL* c.-844C>T** |  |  |  |  |  |  |  |
| Present + TT | 11/14 | 0.46 | Reference |  | 12/14 |  | Reference |
| Null + CT or CC | 29/24 |  | 1.31 (0.62-2.78) |  | 19/24 | 0.53 | 1.27 (0.61-2.64) |
| Present + TT or CT | 4/5 | 0.94 | 1.03 (0.37-2.88) |  | 5/5 | 0.48 | 1.39 (0.55-3.50) |
| Null + CC | 51/65 |  | Reference |  | 53/65 |  | Reference |
| ***GSTP1* c.313A>G *+ XPC* c.2815A>C** |  |  |  |  |  |  |  |
| AA + AA | 29/35 | 0.43 | 1.30 (0.67-2.51) |  | 30/35 | 0.91 | 1.03 (0.56-1.90) |
| AG or GG + AC+CC | 13/21 |  | Reference |  | 16/21 |  | Reference |
| AA or AG + AA or AC | 1/1 | 0.22 | 3.48 (0.47-25.83) |  | 1/1 | 0.90 | 1.13 (0.15-8.17) |
| GG + CC | 67/91 |  | Reference |  | 74/91 |  | Reference |
| ***GSTP1* c.313A>G *+ XPD* c.934G>A** |  |  |  |  |  |  |  |
| AA + GG | 21/27 | 0.47 | 1.25 (0.67-2.35) |  | 23/27 | 0.58 | 1.17 (0.65-2.12) |
| AG or GG + GA or AA | 20/31 |  | Reference |  | 23/31 |  | Reference |
| AA or AG + GG or GA | 2/2 | 0.09 | 3.40 (0.80-14.34) |  | 2/2 | **0.04** | **4.29 (1.00-18.30)** |
| GG or AA | 69/94 |  | Reference |  | 76/94 |  | Reference |
| ***GSTP1* c.313A>G *+ XPD* c.2251A>C** |  |  |  |  |  |  |  |
| AA + AA | 24/28 | 0.54 | 1.22 (0.63-2.36) |  | 25/28 | 0.49 | 1.22 (0.67-2.22) |
| AG or GG + AC or CC | 19/28 |  | Reference |  | 21/28 |  | Reference |
| AA or AG + AA or AC | 2/2 | 0.14 | 2.92 (0.69-12.22) |  | 2/2 | 0.07 | 3.74 (0.88-15.86) |
| GG + CC | 72/96 |  | Reference |  | 78/96 |  | Reference |
| ***GSTP1* c.313A>G *+ XPF* c.2505T>C** |  |  |  |  |  |  |  |
| AA + TT | 24/29 | 0.46 | 1.26 (0.68-2.33) |  | 24/29 | 0.75 | 1.10 (0.60-1.99) |
| AG or GG + TC or CC | 18/26 |  | Reference |  | 21/26 |  | Reference |
| AA or AG + TT or TC | 1/1 | 0.30 | 2.83 (0.38-20.81) |  | 1/1 | 0.18 | 3.88 (0.52-28.83) |
| GG + CC | 73/96 |  | Reference |  | 80/96 |  | Reference |
| ***GSTP1* c.313A>G + *ERCC1* c.354C>T** |  |  |  |  |  |  |  |
| AA + CC | 31/40 |  | Reference |  | 33/40 |  | Reference |
| AG or GG + CT or TT | 12/13 | **0.02** | **2.14 (1.08-4.25)** |  | 13/13 | **0.01** | **2.29 (1.17-4.45)** |
| AA or AG + CC or CT | 1/1 | 0.52 | 1.89 (0.26-13.86) |  | 0/1 | 0.41 | 20.93(0.01-31254.07) |
| GG + TT | 66/84 |  | Reference |  | 72/84 |  | Reference |
| ***GSTP1* c.313A>G *+ MLH1* c.93G>A** |  |  |  |  |  |  |  |
| AA + GG | 17/22 | 0.88 | Reference |  | 20/22 | 0.89 | Reference |
| AG or GG + GA or AA | 20/23 |  | 1.04 (0.55-1.97) |  | 25/30 |  | 1.04 (0.57-1.89) |
| AA or AG + GG or GA |  |  | NE |  |  |  | NE |
| GG + AA |  |  |  |  |  |  |  |
| ***GSTP1* c.313A>G *+ MSH2* c.211+9C>G** |  |  |  |  |  |  |  |
| AA + CC | 33/44 | 0.68 | 1.16 (0.55-2.45) |  | 35/44 | 0.66 | 1.17 (0.56-2.45) |
| AG or GG + GC or GG | 9/14 |  | Reference |  | 9/14 |  | Reference |
| AA or AG + CC or GC | 2/2 | 0.35 | 1.95 (0.46-8.18) |  | 0/2 | 0.29 | 21.66 (0.07-6560.45) |
| GG + GG | 57/78 |  | Reference |  | 63/78 |  | Reference |
| ***GSTP1* c.313A>G *+ MSH3* c.3133G>A** |  |  |  |  |  |  |  |
| AA + GG | 40/52 | 0.93 | Reference |  | 44/52 | 0.48 | Reference |
| AG or GG + GA or AA | 5/7 |  | 1.03 (0.40-2.64) |  | 5/7 |  | 1.39 (0.55-3.52) |
| AA or AG + GG or GA | 5/5 | **0.05** | **2.60 (0.96-7.01)** |  | 44/52 | 0.93 | 1.04 (0.37-2.92) |
| GG + AA | 35/47 |  | Reference |  | 5/7 |  | Reference |
| ***GSTP1* c.313A>G *+ EXO1* c.1762G>A** |  |  |  |  |  |  |  |
| AA + GG | 24/32 | 0.09 | Reference |  | 25/32 | 0.13 | Reference |
| AG or GG + GA or AA | 18/22 |  | 1.69 (0.91-3.14) |  | 19/22 |  | 1.57 (0.86-2.86) |
| AA or AG + GG or GA | 1/1 | 0.46 | 2.09 (0.28-15.26) |  | 1/1 | 0.58 | 1.74 (0.24-12.68) |
| GG + AA | 70/93 |  | Reference |  | 76/93 |  | Reference |
| ***GSTP1* c.313A>G *+ TP53* c.215G>C** |  |  |  |  |  |  |  |
| AA + CC | 38/48 |  | Reference |  | 40/48 |  | Reference |
| AG or GG +GC or GG | 3/4 | 0.87 | 1.09 (0.33-3.57) |  | 3/4 | 0.98 | 1.00 (0.31-3.28) |
| AA or AG + CC or GC | 4/4 | **0.05** | **2.85 (0.97-8.33)** |  | 3/ 4 | 0.88 | 1.09 (0.33-3.52) |
| GG + GG | 41/56 |  | Reference |  | 46/56 |  | Reference |
| ***GSTP1* c.313A>G *+ CASP3* c.-1191A>G** |  |  |  |  |  |  |  |
| AA + GG | 39/48 | 0.41 | 1.80 (0.43-7.52) |  | 39/48 | 0.46 | 1.71 ((0.41-7.13) |
| AG or GG + AG or AA | 2/4 |  | Reference |  | 2//4 |  | Reference |
| AA or AG + GG or AG | 4/4 | **0.04** | **2.94 (1.00-8.60)** |  | 3/ 4 | 0.63 | 1.33 (0.41-4.29) |
| GG + AA | 44/62 |  | Reference |  | 50/62 |  | Reference |
| ***GSTP1* c.313A>G + *CASP3* c.-182-247G>T** |  |  |  |  |  |  |  |
| AA + TT | 39/50 |  | Reference |  | 41/50 |  | Reference |
| AG or GG + GT or TT | 7/8 | 0.67 | 1.19 (0.53-2.66) |  | 7/8 | 0.54 | 1.28 (0.57-2.87) |
| AA or AG + TT or GT | 2/2 | 0.11 | 3.21 (0.75-13.73) |  | 2/2 | 0.88 | 1.11 (0.27-4.58) |
| GG + GG | 52/66 |  | Reference |  | 53/66 |  | Reference |
| ***GSTP1* c.313A>G + *FAS* c.-1378G>A** |  |  |  |  |  |  |  |
| AA + AA | 40/51 | 0.45 | 1.72 (0.41-7.18) |  | 42/51 | 0.30 | 2.10 (0.50-8.79) |
| AG or GG + GA or GG | 2/3 |  | Reference |  | 2/3 |  | Reference |
| AA or AG + AA or GA | 4/4 | 0.08 | 2.80 (0.87-9.01) |  | 2/4 | 0.47 | 1.69 (0.39-7.35) |
| GG + GG | 21/27 |  | Reference |  | 22/27 |  | Reference |
| ***GSTP1* c.313A>G *+ FAS* c.-671A>G** |  |  |  |  |  |  |  |
| AA or AG + GG or AG | 2/2 | 0.20 | 2.53 (0.60-10.69) |  | 1/ 2 | 0.31 | 2.76 (0.38-20.03) |
| GG + AA | 55/71 |  | Reference |  | 62/71 |  | Reference |
| ***GSTP1* c.313A>G *+ FASL* c.-844C>T** |  |  |  |  |  |  |  |
| AA + TT | 34/44 | 0.48 | Reference |  | 37/44 | 0.36 | Reference |
| AG or GG + CT or CC | 12/16 |  | 1.26 (0.65-2.45) |  | 13/16 |  | 1.34 (0.71-2.53) |
| AA or AG + TT or CT | 3/3 | 0.06 | 3.10 (0.92-10.38) |  | 3/3 | 0.31 | 1.81 (0.56-5.83) |
| GG + CC | 57/74 |  | Reference |  | 61/74 |  | Reference |
| ***XPC* c.2815A>C *+ XPD* c.934G>A** |  |  |  |  |  |  |  |
| AA + GG | 27/34 | 0.07 | 1.88 (0.95-3.73) |  | 31/34 | 0.14 | 1.56 (0.86-2.83) |
| AC or CC + GA or AA | 13/25 |  | Reference |  | 18/25 |  | Reference |
| AA or AC + GG or GA | 2/2 | 0.33 | 2.02 (0.49-8.33) |  | 2/2 | 0.42 | 1.78 (0.43-7.37) |
| CC + AA | 64/87 |  | Reference |  | 68/87 |  | Reference |
| ***XPC* c.2815A>C *+ XPD* c.2251A>C** |  |  |  |  |  |  |  |
| AA + AA | 31/37 | **0.05** | **1.93 (0.99-3.77)** |  | 34/37 | 0.17 | 1.51 (0.83-2.75) |
| AC or CC + AC or CC | 13/24 |  | Reference |  | 17/24 |  | Reference |
| AA or AC + AA or AC | 1/1 | 0.54 | 1.86 (0.25-13.58) |  | 1/1 | 0.44 | 2.15 (0.29-15.79) |
| CC + CC | 66/88 |  | Reference |  | 69/88 |  | Reference |
| ***XPC* c.2815A>C *+ XPF* c.2505T>C** |  |  |  |  |  |  |  |
| AA + TT | 34/40 | 0.12 | 1.62 (0.88-2.99) |  | 33/40 | 0.26 | 1.39 (0.77-2.52) |
| AC or CC + TC or CC | 15/24 |  | Reference |  | 17/24 |  | Reference |
| AA or AC + TT or TC |  |  | NE |  |  |  | NE |
| CC + CC |  |  |  |  |  |  |  |
| ***XPC* c.2815A>C + *ERCC1* c.354C>T** |  |  |  |  |  |  |  |
| AA + CC | 39/48 |  | Reference |  | 40/48 |  | Reference |
| AC or CC + CT or TT | 7/8 | 0.49 | 1.33 (0.59-3.00) |  | 7/8 | 0.57 | 0.79 (0.35-1.77) |
| AA or AC + CC or CT | 1/1 | 0.07 | 6.61 (0.85-51.25) |  | 1/1 | **0.04** | **8.15 (1.03-64.38)** |
| CC + TT | 61/77 |  | Reference |  | 65/77 |  | Reference |
| ***XPC* c.2815A>C *+ MLH1* c.93G>A** |  |  |  |  |  |  |  |
| AA + GG | 24/31 | 0.36 | 1.33 (0.71-2.48) |  | 26/31 | 0.36 | 1.32 (0.72-2.41) |
| AC or CC + GA or AA | 17/26 |  | Reference |  | 28/26 |  | Reference |
| AA or AC + GG or GA |  |  | NE |  |  |  | NE |
| CC + AA |  |  |  |  |  |  |  |
| ***XPC* c.2815A>C *+ MSH2* c.211+9C>G** |  |  |  |  |  |  |  |
| AA + CC | 45/53 | 0.46 | 1.32 (0.62-2.82) |  | 47/53 | 0.64 | 1.19 (0.56-2.53) |
| AC or CC + CG or GG | 8/10 |  | Reference |  | 8/10 |  | Reference |
| AA or AC + CC or CG | 3/5 |  | Reference |  | 4/5 |  | Reference |
| CC + GG | 53/74 | 0.62 | 1.33 (0.41-4.27) |  | 59/74 | 0.88 | 1.07 (0.39-2.96) |
| ***XPC* c.2815A>C *+ EXO1* c.1762G>A** |  |  |  |  |  |  |  |
| AA + GG | 28/35 | 0.71 | Reference |  | 29/35 | 0.70 | Reference |
| AC or CC + GA or AA | 9/12 |  | 1.15 (0.54-2.45) |  | 10/12 |  | 1.14 (0.55-2.35) |
| AA or AC + GG or GA | 1/1 |  | Reference |  | 1/1 |  | Reference |
| CC + AA | 65/86 | 0.95 | 1.05 (0.14-7.65) |  | 68/86 | 0.94 | 1.07 (0.14-7.77) |
| ***XPD* c.934G>A *+ XPD* c.2251A>C** |  |  |  |  |  |  |  |
| GG + AA | 35/42 | 0.10 | 1.49 (0.91-2.42) |  | 37/42 | 0.12 | 1.45 (0.90-2.32) |
| GA or AA + AC or CC | 32/47 |  | Reference |  | 34/47 |  | Reference |
| GG or GA + AA or AC | 4/5 | 0.35 | 1.61 (0.58-4.45) |  | 5/5 | 0.19 | 1.83 (0.73-4.56) |
| AA + CC | 70/94 |  | Reference |  | 74/94 |  | Reference |
| ***XPD* c.934G>A *+ XPF* c.2505T>C** |  |  |  |  |  |  |  |
| GG + TT | 24/27 | **0.05** | **1.83 (0.99-3.38)** |  | 25/27 | **0.03** | **1.86 (1.03-3.34)** |
| GA or AA + TC or CC | 20/29 |  | Reference |  | 23/29 |  | Reference |
| GG or GA + TT or TC | 2/3 | 0.88 | 1.11 (0.27-4.56) |  | 3/3 | 0.36 | 1.71 (0.53-5.48) |
| AA + CC | 70/93 |  | Reference |  | 75/93 |  | Reference |
| ***XPD* c.934G>A *+ MLH1* c.93G>A** |  |  |  |  |  |  |  |
| GG + GG | 17/20 | 0.12 | 1.62 (0.86-3.05) |  | 20/20 | 0.06 | 1.75 (0.96-3.19) |
| GA or AA + GA or AA | 25/33 |  | Reference |  | 26/33 |  | Reference |
| GG or GA + GG or GA |  |  | NE |  |  |  | NE |
| AA + AA |  |  |  |  |  |  |  |
| ***XPD* c.934G>A *+ MSH2* c.211+9C>G** |  |  |  |  |  |  |  |
| GG + CC | 33/41 | 0.25 | 1.49 (0.75-2.97) |  | 36/41 | 0.17 | 1.59 (0.80-3.14) |
| GA or AA + GC or GG | 11/16 |  | Reference |  | 11/16 |  | Reference |
| GG or GA + CC or GC | 1/ 2 |  | Reference |  | 2/2 |  | Reference |
| AA + GG | 52/73 | 0.59 | 1.72 (0.23-12.48) |  | 58/73 | 0.84 | 1.14 (0.27-4.72) |
| ***XPD* c.934G>A *+ EXO1* c.1762G>A** |  |  |  |  |  |  |  |
| GG + GG | 23/28 | 0.71 | Reference |  | 24/28 | 0.91 | Reference |
| GA or AA + GA or AA | 19/23 |  | 1.12 (0.61-2.06) |  | 19/23 |  | 1.03 (0.56-1.89) |
| GG or GA + GG or GA | 0/1 |  | NE |  |  |  | NE |
| AA + AA | 65/88 |  |  |  |  |  |  |
| ***XPD* c.934G>A *+ CASP3* c.-1191A>G** |  |  |  |  |  |  |  |
| GG + GG | 39/45 | 0.27 | 1.77 (0.63-4.98) |  | 40/45 | 0.33 | 1.65 (0.59-4.64) |
| GA or AA + AG or AA | 4/6 |  | Reference |  | 4/6 |  | Reference |
| GG or GA + GG or AG | 5/5 | **0.03** | **2.81 (1.08-7.32)** |  | 5/5 | **0.01** | **3.58 (1.35-9.45)** |
| AA + AA | 41/58 |  | Reference |  | 45/58 |  | Reference |
| ***XPD* c.934G>A *+ FAS* c.-671A>G** |  |  |  |  |  |  |  |
| GG + GG | 28/37 | 0.74 | 1.13 (0.53-2.41) |  | 31/37 | 0.82 | 1.08 (0.52-2.22) |
| GA or AA + AG or AA | 9/13 |  | Reference |  | 10/13 |  | Reference |
| GG or GA + GG or AG | 5/5 | **0.006** | **3.89 (1.47-10.26)** |  | 5/5 | **0.04** | **2.67 (1.04-6.84)** |
| AA + AA | 54/69 |  | Reference |  | 59/69 |  | Reference |
| ***XPD* c.934G>A *+ FASL* c.-844C>T** |  |  |  |  |  |  |  |
| GG + TT | 31/38 | 0.82 | 1.08 (0.54-2.16) |  | 33/38 | 0.42 | 1.33 (0.65-2.71) |
| GA or AA + CT or CC | 11/15 |  | Reference |  | 10/15 |  | Reference |
| GG or GA + TT or CT | 5/5 | **0.01** | **3.33 (1.28-8.64)** |  | 5/5 | **0.009** | **3.58 (1.37-9.33)** |
| AA + CC | 55/71 |  | Reference |  | 56/71 |  | Reference |
| ***XPD* c.2251A>C *+ XPF* c.2505 T>C** |  |  |  |  |  |  |  |
| AA + TT | 24/28 | **0.04** | **1.98 (1.03-3.80)** |  | 25/28 | **0.05** | **1.84 (0.99-3.40)** |
| AC or CC + TC or CC | 16/26 |  | Reference |  | 19/26 |  | Reference |
| AA or AC + TT or TC | 2/3 | 0.99 | 1.00 (0.24-4.12) |  | 3/3 | 0.46 | 1.53 (0.48-4.88) |
| CC + CC | 73/95 |  | Reference |  | 77/95 |  | Reference |
| ***XPD* c.2251A>C *+ ERCC1* c.354C>T** |  |  |  |  |  |  |  |
| AA + CC | 27/34 | 0.51 | 1.34 (0.55-3.25) |  | 28/34 | 0.33 | 1.59 (0.61-4.14) |
| AC or CC + CT or TT | 6/8 |  | Reference |  | 5/8 |  | Reference |
| AA or AC + CC or CT |  |  | NE |  |  |  | NE |
| CC + TT |  |  |  |  |  |  |  |
| ***XPD* c.2251A>C *+ MLH1* c.93G>A** |  |  |  |  |  |  |  |
| AA + GG | 18/22 | 0.14 | 1.59 (0.84-3.00) |  | 21/22 | 0.11 | 1.63 (0.89-2.97) |
| AC or CC + GA or AA | 22/31 |  | Reference |  | 23/31 |  | Reference |
| AA or AC + GG or GA |  |  | NE |  |  |  | NE |
| CC + AA |  |  |  |  |  |  |  |
| ***XPD* c.2251A>C *+ MSH2* c.211+9C>G** |  |  |  |  |  |  |  |
| AA + CC | 35/43 | 0.17 | 1.67 (0.80-3.48) |  | 38/43 | 0.14 | 1.71 (0.82-3.55) |
| AC or CC + CG or GG | 9/14 |  | Reference |  | 9/14 |  | Reference |
| AA or AC + CC or CG | 1/ 2 |  | Reference |  | 2/2 |  | Reference |
| CC + GG | 55/75 | 0.61 | 1.67 (0.23-12.12) |  | 60/75 | 0.82 | 1.17 (0.28-4.82) |
| ***XPD* c.2251A>C *+ EXO1* c.1762G>A** |  |  |  |  |  |  |  |
| AA + GG | 24/29 | 0.76 | Reference |  | 25/29 | 0.99 | Reference |
| AC or CC + GA or AA | 16/20 |  | 1.10 (0.58-2.08) |  | 16/20 |  | 1.00 (0.53-1.88) |
| AA or AC + GG or GA | 0/1 |  | NE |  |  |  | NE |
| CC + AA | 68/90 |  |  |  |  |  |  |
| ***XPD* c.2251A>C *+ TP53* c.215G>C** |  |  |  |  |  |  |  |
| AA + CC | 42/50 | 0.40 | 1.48 (0.58-3.77) |  | 45/50 | 0.50 | 1.37 (0.54-3.47) |
| AC or CC + GC or GG | 5/7 |  | Reference |  | 5/7 |  | Reference |
| AA or AC + CC or GC | 3/3 | 0.38 | 1.68 (0.51-5.49) |  | 3/3 | 0.62 | 1.34 (0.41-4.36) |
| CC + GG | 39/52 |  | Reference |  | 41/52 |  | Reference |
| ***XPD* c.2251A>C *+ CASP3* c.-1191A>G** |  |  |  |  |  |  |  |
| AA + GG | 42/49 | 0.13 | 2.47 (0.76-8.00) |  | 44/49 | 0.29 | 1.73 (0.62-4.84) |
| AC or CC + AG or AA | 3/6 |  | Reference |  | 4/6 |  | Reference |
| AA or AC + GG or AG | 4/4 | 0.31 | 1.69 (0.60-4.75) |  | 4/4 | 0.27 | 1.78 (0.63-4.97) |
| CC + AA | 43/59 |  | Reference |  | 46/59 |  | Reference |
| ***XPD* c.2251A>C *+ FAS* c.-671A>G** |  |  |  |  |  |  |  |
| AA + GG | 31//39 | 0.88 | 1.05 (0.48-2.31) |  | 33/39 | 0.91 | 1.04 (0.48-2.26) |
| AC or CC + AG or AA | 8/11 |  | Reference |  | 8/11 |  | Reference |
| AA or AC + GG or AG | 3/3 | 0.57 | 1.40 (0.43-4.50) |  | 3/3 | 0.84 | 1.12 (0.35-3.60) |
| CC + AA | 55/69 |  | Reference |  | 59/69 |  | Reference |
| ***XPD* c.2251A>C *+ FASL* c.-844C>T** |  |  |  |  |  |  |  |
| AA + TT | 37/44 | 0.84 | Reference |  | 39/44 | 0.58 | Reference |
| AC or CC + CT or CC | 13/17 |  | 1.06 (0.56-2.00) |  | 12/17 |  | 1.19 (0.62-2.29) |
| AA or AC + TT or CT | 2/2 | 0.14 | 2.98 (0.70-12.70) |  | 2/2 | 0.07 | 3.83 (0.88-16.58) |
| CC + CC | 55/70 |  | Reference |  | 55/70 |  | Reference |
| ***XPF* c.2505T>C *+ ERCC1* c.354C>T** |  |  |  |  |  |  |  |
| TT + CC | 30/41 | 0.76 | 1.13 (0.51-2.47) |  | 32/41 | 0.90 | 1.04 (0.51-2.13) |
| TC or CC + CT or TT | 8/12 |  | Reference |  | 10/12 |  | Reference |
| TT or TC + CC or CT |  |  | NE |  |  |  | NE |
| CC + TT |  |  |  |  |  |  |  |
| ***XPF* c.2505T>C *+ MLH1* c.93G>A** |  |  |  |  |  |  |  |
| TT + GG | 19/24 | 0.31 | 1.41 (0.74-2.54) |  | 20/24 | 0.27 | 1.40 (0.76-2.56) |
| TC or CC + GA or AA | 22/30 |  | Reference |  | 23/30 |  | Reference |
| TT or TC + GG or GA | 1/1 | 0.14 | 4.44 (0.59-33.11) |  | 1/1 | 0.10 | 5.30 (0.70-39.86) |
| CC + AA | 76/98 |  | Reference |  | 81/98 |  | Reference |
| ***XPF* c.2505T>C *+ MSH2* c.211+9C>G** |  |  |  |  |  |  |  |
| TT + CC | 36/44 | 0.95 | 1.02 (0.49-2.13) |  | 37/44 | 0.93 | 1.02 (0.49-2.14) |
| TC or CC + CG or GG | 9/12 |  | Reference |  | 9/12 |  | Reference |
| TT or TC + CC or GC | 1/ 2 | 0.75 | 1.36 (0.18-9.87) |  | 2/2 | 0.65 | 1.38 (0.33-5.68) |
| CC + GG | 57/76 |  | Reference |  | 63/76 |  | Reference |
| ***XPF* c.2505T>C *+ MSH3* c.3133G>A** |  |  |  |  |  |  |  |
| TT + GG | 43/54 | 0.84 | 1.10 (0.43-2.79) |  | 45/54 | 0.18 | 1.99 (0.71-5.57) |
| TC or CC + GA or AA | 5/7 |  | Reference |  | 4/7 |  | Reference |
| TT or TC + GG or GA | 3/5 | 0.48 | Reference |  | 3/5 | 0.44 | Reference |
| CC + AA | 34/45 |  | 1.53 (0.46-5.12) |  | 37/45 |  | 1.59 (0.48-5.29) |
| ***XPF* c.2505T>C *+ CASP3* c.-182-247G>T** |  |  |  |  |  |  |  |
| TT + TT | 40/50 | 0.80 | 1.12 (0.44-2.85) |  | 5/3 | 0.41 | 1.62 (0.50-5.21) |
| TT or TC + GT or GG | 5/6 |  | Reference |  | 52/67 |  | Reference |
| TT or TC + TT or GT | 2/5 | 0.20 | Reference |  | 3/5 | 0.41 | Reference |
| CC + GG | 53/67 |  | 2.51 (0.61-10.36) |  | 52/67 |  | 1.62 (0.50-5.21) |
| ***ERCC1* c.354C>T *+ MSH3* c.3133G>A** |  |  |  |  |  |  |  |
| CC + GG | 54/74 |  | Reference |  | 60/74 |  | Reference |
| CT or TT + GA or AA | 3/3 | 0.11 | 2.56 (0.79-8.25) |  | 2/3 | 0.71 | 1.29 (0.31-5.32) |
| CC or CT + GG or GA | 8/14 | **0.06** | Reference |  | 8/14 | **0.02** | Reference |
| TT + AA | 32/42 |  | **2.12 (0.96-4.69)** |  | 35/42 |  | **2.38 (1.09-5.20)** |
| ***ERCC1* c.354C>T *+ CASP3* c.-1191A>G** |  |  |  |  |  |  |  |
| CC + GG | 57/75 |  | Reference |  | 61/75 |  | Reference |
| CC or CT + AG or AA | 4/5 | 0.78 | 1.15 (0.41-3.19) |  | 5/5 | 0.32 | 1.58 (0.63-3.98) |
| CC or CT + GG or AG | 6/11 | 0.27 | Reference |  | 7/11 | 0.31 | Reference |
| TT + AA | 40/55 |  | 1.60 (0.68-3.79) |  | 45/55 |  | 1/50 (0.67-3.32) |
| ***ERCC1* c.354C>T *+ FAS* c.-1378G>A** |  |  |  |  |  |  |  |
| CC + AA |  |  | NE |  |  |  | NE |
| CT or TT + GA or GG |  |  |  |  |  |  |  |
| CC or CT + AA or GA | 10/16 | 0.18 | Reference |  | 11/16 | 0.17 | Reference |
| TT + GG | 21/25 |  | 1.66 (0.78-3.55) |  | 22/25 |  | 1.64 (0.79-3.41) |
| ***ERCC1* c.354C>T *+ FASL* c.-844C>T** |  |  |  |  |  |  |  |
| CC + TT | 44/60 |  | Reference |  | 48/60 |  | Reference |
| CT or TT + CT or CC | 6/6 | **0.04** | **2.38 (1.00-5.67)** |  | 5/6 | 0.23 | 1.76 (0.69-4.48) |
| CC or CT + TT or CT | 4/9 |  | Reference |  | 6/9 | 0.08 | Reference |
| TT + CC | 52/65 | **0.02** | **3.11 (1.12-8.63)** |  | 55/66 |  | 2.10 (0.89-4.90) |
| ***MLH1* c.93G>A *+ MSH2* c.211+9C>G** |  |  |  |  |  |  |  |
| GG + CC | 25/34 | 0.99 | Reference |  | 30/34 | 0.69 | Reference |
| GA or AA + CG or GG | 10/13 |  | 1.00 (0.48-2.09) |  | 10/13 |  | 1.15 (0.56-2.36) |
| GG or GA + CC or CG | 1/1 |  | Reference |  | 1/1 |  | Reference |
| AA + GG | 59/79 | 0.84 | 1.21 (0.16-8.83) |  | 65/79 | 0.75 | 1.36 (0.18-9.87) |
| ***MLH1* c.93G>A *+ MSH3* c.3133G>A** |  |  |  |  |  |  |  |
| GG + GG | 31/43 | 0.95 | 1.03 (0.40-2.65) |  | 37/43 | 0.18 | 2.00 (0.71-5.64) |
| GA or AA + GA or AA | 5/7 |  | Reference |  | 4/7 |  | Reference |
| GG or GA + GG or GA | 2/2 |  | Reference |  | 2/2 |  | Reference |
| AA + AA | 35/46 | 0.95 | 1.03 (0.24-4.35) |  | 39/46 | 0.67 | 1.35 (0.32-5.65) |
| ***MLH1* c.93G>A *+ EXO1* c.1762G>A** |  |  |  |  |  |  |  |
| GG + GG | 16/23 | 0.29 | Reference |  | 19/23 | 0.58 | Reference |
| GA or AA + GA or AA | 19/22 |  | 1.43 (0.73-2.79) |  | 19/22 |  | 1.19 (0.63-2.25) |
| GG or GA + GG or GA |  |  | NE |  |  |  | NE |
| AA or AA |  |  |  |  |  |  |  |
| ***MLH1* c.93G>A *+ FAS* c.-1378G>A** |  |  |  |  |  |  |  |
| GG + AA | 32/44 | 0.40 | 1.65 (0.50-5.44) |  | 37/44 | 0.22 | 2.07 (0.63-6.77) |
| GA or AA + GA or GG | 3/5 |  | Reference |  | 3/5 |  | Reference |
| GG or GA + AA or GA | 1/1 |  | Reference |  | 1 2 |  | Reference |
| AA + GG | 21/27 | 0.63 | 1.62 (0.21-12.17) |  | 22/27 | 0.41 | 2.31 (0.30-17.35) |
| ***MLH1* c.93G>A *+ FASL* c.-844C>T** |  |  |  |  |  |  |  |
| GG + TT | 25/33 | 0.54 | Reference |  | 29/33 | 0.99 | Reference |
| GA or AA + CT or CC | 12/14 |  | 1.24 (0.62-2.49) |  | 11/14 |  | 1.00 (0.49-2.01) |
| GG or GA + TT or CT | 1/1 |  | Reference |  | 1/1 |  | Reference |
| AA + CC | 58/74 | 0.73 | 1.40 (0.19-10.19) |  | 60/74 | 0.76 | 1.36 (0.18-9.87) |
| ***MSH2* c.211+9C>G *+ EXO1* c.1762G>A** |  |  |  |  |  |  |  |
| CC + GG | 34/49 | 0.69 | 1.19 (0.49-2.90) |  | 38/49 | 0.57 | 1.25 (0.56-2.83) |
| CG or GG + GA or AA | 7/10 |  | Reference |  | 7/10 |  | Reference |
| CC or CG + GG or GA | 2/3 |  | Reference |  | 3/3 |  | Reference |
| GG + AA | 55/74 | 0.54 | 1.54 (0.37-6.35) |  | 60/74 | 0.90 | 1.07 (0.33-3.42) |
| ***MSH2* c.211+9C>G *+ CASP3* c.-1191A>G** |  |  |  |  |  |  |  |
| CC + GG | 61/78 |  | 1.61 (0.50-5.15) |  | 65/78 | 0.41 | 1.62 (0.50-5.18) |
| CG or GG + AG or AA | 3/5 | 0.42 | Reference |  | 3/5 |  | Reference |
| CC or CG + GG or AG | 13/15 |  | 1.30 (0.69-2.46) |  | 12/15 | 0.66 | 1.15 (0.60-2.20) |
| GG + AA | 37/52 | 0.41 | Reference |  | 41/52 |  | Reference |
| ***MSH2* c.211+9C>G *+ FAS* c.-1378G>A** |  |  |  |  |  |  |  |
| CC + AA | 61/80 |  | Reference |  | 67/80 |  | Reference |
| CG or GG + GA or GG | 2/3 | 0.85 | 1.14 (0.27-4.71) |  | 2/3 | 0.84 | 1.14 (0.28-4.69) |
| CC or CG + AA or GA | 18/22 | 0.68 | 1.14 (0.59-2.22) |  | 18/22 | 0.77 | 1.09 (0.57-2.11) |
| GG + GG | 19/24 |  | Reference |  | 20/24 |  | Reference |
| ***MSH2* c.211+9C>G *+ FAS* c.-671A>G** |  |  |  |  |  |  |  |
| CC + GG | 46/62 |  | Reference |  | 51/62 |  | Reference |
| CG or GG + AG or AA | 4/4 | **0.0001** | **11.18 (3.56-35.07)** |  | 4/4 | **0.001** | **6.15 (2.12-17.82)** |
| CC or CG+ AG or GG | 5/7 |  | Reference |  | 5/7 | 0.49 | Reference |
| GG + AA | 42/55 | 0.88 | 1.06 (0.41-2.74) |  | 48/55 |  | 1.38 (0.54-3.48) |
| ***MSH2* c.211+9C>G *+ FASL* c.-844C>T** |  |  |  |  |  |  |  |
| CC + TT | 47/65 | 0.31 | 1.68 (0.60-4.70) |  | 53/65 | 0.20 | 1.94 (0.70-5.37) |
| CG or GG + CT or CC | 4/8 |  | Reference |  | 4/8 |  | Reference |
| CC or CG + TT or CT | 3/5 | 0.63 | 1.32 (0.40-4.28) |  | 5/5 | 0.66 | 1.22 (0.48-3.10) |
| GG or CC | 41/55 |  | Reference |  | 45/55 |  | Reference |
| ***MSH3* c.3133G>A *+ TP53* c.215G>C** |  |  |  |  |  |  |  |
| GG + CC | 69/90 |  | Reference |  | 76/90 |  | Reference |
| GA or AA + GC or GG | 2/2 | 0.49 | 1.63 (0.39-6.69) |  | 1/2 | 0.70 | 1.46 (0.20-10.58) |
| GG or GA + CC or GC | 19/26 | 0.85 | 1.06 (0.53-2.11) |  | 20/26 | 0.61 | 1.18 (0.61-2.26) |
| AA + GG | 15/23 |  | Reference |  | 18/23 |  | Reference |
| ***MSH3* c.3133G>A *+ CASP3* c.-1191A>G** |  |  |  |  |  |  |  |
| GG + GG |  |  | NE |  |  |  | NE |
| GA or AA + AG or AA |  |  |  |  |  |  |  |
| GG or GA + GG or AG | 21/25 | 0.56 | 1.19 (0.64-2.21) |  | 21/25 | 0.63 | 1.15 (0.63-2.08) |
| AA + AA | 20/28 |  | Reference |  | 23/28 |  | Reference |
| ***MSH3*c.3133G>A *+ CASP3*c.-182-247G>T** |  |  |  |  |  |  |  |
| GG + TT | 65/87 |  | Reference |  | 73/87 |  | Reference |
| GA or AA + GT or GG | 1/1 | **0.02** | **10.55 (1.32-84.37)** |  | 1/1 | 0.16 | 4.10 (0.54-30.66) |
| GG or GA + TT or GT | 18/24 | 0.61 | Reference |  | 21/24 | 0.89 | Reference |
| AA + GG | 27/33 |  | 1.16 (0.64-2.12) |  | 27/33 |  | 1.03 (0;58-1.83) |
| ***MSH3* c.3133G>A *+ FAS* c.-671A>G** |  |  |  |  |  |  |  |
| GG + GG | 56/76 |  | Reference |  | 62/76 |  | Reference |
| GA or AA + AG or AA | 3/3 | 0.19 | 2.16 (0.67-6.95) |  | 2/3 | 0.76 | 1.24 (0.30-5.11) |
| GG or GA + GG or AG | 11/17 | 0.46 | Reference |  | 11/17 | 0.18 | Reference |
| AA + AA | 23/31 |  | 1.31 (0.63-2.70) |  | 27/31 |  | 11.62 (0.80-3.28) |
| ***MSH3* c.3133G>A *+ FASL* c.-844C>T** |  |  |  |  |  |  |  |
| GG + TT | 56/74 |  | Reference |  | 63/74 |  | Reference |
| GA or AA + CT or CC | 2/2 | 0.69 | 1.12 (0.62-2.01) |  | 1/2 | 0.60 | 1.67 (0.23-12.12) |
| GG or GA + TT or CT | 18/23 | 0.69 | Reference |  | 19/23 | 0.85 | Reference |
| AA + CC | 31/39 |  | 1.21 (0.62-2.01) |  | 32/39 |  | 1.05 (0.59-1.86) |
| ***EXO1* c.1762G>A *+ FAS* c.-1378G>A** |  |  |  |  |  |  |  |
| GG + AA | 44/62 | 0.99 | 1.00 (0.31-3.25) |  | 48/62 | 0.78 | 1.17 (0.36-3.79) |
| GA or AA + GA or GG | 3/5 |  | Reference |  | 3/5 |  | Reference |
| GG or GA + AA or GA | 6/9 |  | Reference |  | 8/9 |  | Reference |
| AA + GG | 21/27 | 0.69 | 1.20 (0.48-3.01) |  | 22/27 | 0.78 | 1.12 (0.49-2.53) |
| ***EXO1* c.1762G>A *+ FASL* c.-844C>T** |  |  |  |  |  |  |  |
| GG + TT | 35/51 | 0.51 | Reference |  | 40/51 | 0.52 | Reference |
| GA or AA + CT or CC | 10/14 |  | 1.26 (0.62-2.56) |  | 11/14 |  | 1.24 (0.63-2.43) |
| GG or GA + TT or CT | 2/4 |  | Reference |  | 3/ 4 |  | Reference |
| AA + CC | 54/70 | 0.26 | 2.24 (0.54-9.24) |  | 55/70 | 0.71 | 1.23 (0.38-3.97) |
| ***TP53* c.215G>C *+ CASP3* c.-1191A>G** |  |  |  |  |  |  |  |
| CC + GG | 69/88 |  | NE |  |  |  | NE |
| CG or GG + AG or AA | 0/1 |  |  |  |  |  |  |
| CC or CG+ GG or AG | 16/19 |  | 1.28 (0.67-2.44) |  | 17/19 | 0.49 | 1.23 (0.67-2.28) |
| GG + AA | 22/32 | 0.45 | Reference |  | 26/32 |  | Reference |
| ***TP53* c.215G>C *+ CASP3* c.-182-247G>T** |  |  |  |  |  |  |  |
| CC + TT |  |  | NE |  |  |  | NE |
| GC or GG + GT or GG |  |  |  |  |  |  |  |
| CC or GC+ TT or GT | 14/18 |  | Reference |  | 16/18 | 0.80 | Reference |
| GG + GG | 30/37 | 0.86 | 1.05 (0.55-1.99) |  | 29/37 |  | 1.08 (0.58-1.99) |
| ***TP53* c.215G>C *+ FAS* c.-1378G>A** |  |  |  |  |  |  |  |
| CC + AA |  |  | NE |  |  |  | NE |
| GC or GG + GA or GG |  |  |  |  |  |  |  |
| CC or GC+ GA or AA | 30/38 | 0.88 | 1.05 (0.54-2.01) |  | 32/38 | 0.98 | 1.00 (0.53-1.88) |
| GG + GG | 13/16 |  | Reference |  | 14/16 |  | Reference |
| ***TP53* c.215G>C *+ FAS* c.-671A>G** |  |  |  |  |  |  |  |
| CC + GG |  |  | NE |  |  |  | NE |
| GC or GG + AG or AA |  |  |  |  |  |  |  |
| CC or CG+ AG or GG | 13/17 |  | Reference |  | 12/17 | 0.19 | Reference |
| GG + AA | 32/41 | 0.63 | 1.16 (0.61-2.23) |  | 35/41 |  | 1.53 (0.79-2.97) |
| ***TP53* c.215G>C *+ FASL* c.-844C>T** |  |  |  |  |  |  |  |
| CC + TT |  |  | NE |  |  |  | NE |
| GC or GG + CT or CC |  |  |  |  |  |  |  |
| CC or GC + TT or CT | 10/13 | 0.91 | Reference |  | 11/13 |  | Reference |
| GG + CC | 30/39 |  | 1.04 (0.50-2.13) |  | 31/39 | 0.93 | 1.02 (0.51-2.05) |
| ***CASP3* c.-1191A>G *+ FAS* c.-1378G>A** |  |  |  |  |  |  |  |
| GG + AA |  |  | NE |  |  |  | NE |
| AG or AA + GA or GG |  |  |  |  |  |  |  |
| AG or GG + GA or AA | 26/33 | 0.30 | 1.35 (0.68-2.70) |  | 27/33 | 0.26 | 1.46 (0.75-2.85) |
| AA + GG | 12/17 |  | Reference |  | 13/17 |  | Reference |
| ***CASP3* c.-1191A>G *+ FAS* c.-671A>G** |  |  |  |  |  |  |  |
| GG + GG | 56/73 |  | Reference |  | 59/73 |  | Reference |
| AG or AA + AG or AA | 1/1 | 0.17 | 4.10 (0.54-30.98) |  | 1/1 | 0.19 | 3.80 (0.50-28.60) |
| AG or GG + AG or GG | 9/13 |  | Reference |  | 9/13 |  | Reference |
| AA + AA | 31/43 | 0.80 | 1.09 (0.52-2.30) |  | 36/43 | 0.65 | 1.18 (0.56-2.45) |
| ***CASP3* c.-182-247G>T *+ FAS* c.-1378G>A** |  |  |  |  |  |  |  |
| TT + AA |  |  | NE |  |  |  | NE |
| GT or GG + GA or GG |  |  |  |  |  |  |  |
| TT or GT + AA or GA | 18/26 |  | Reference |  | 24/26 | 0.53 | Reference |
| GG + GG | 14/16 | 0.98 | 1.00 (0.49-2.04) |  | 14/16 |  | 1.23 (0.63-2.41) |
| ***CASP3* c.-182-247G>T *+ FAS* c.-671A>G** |  |  |  |  |  |  |  |
| TT + GG | 51/71 |  | Reference |  | 56/71 |  | Reference |
| GT or GG + AG or AA | 1/1 | 0.91 | 1.11 (0.15-8.11) |  | 1/1 | 0.75 | 1.37 (0.18-10.03) |
| TT or GT + GG or AG | 8/11 |  | Reference |  | 9/11 | 0.66 | Reference |
| GG + AA | 40/47 | 0.63 | 1.20 (0.56-2.58) |  | 40/47 |  | 1.17 (0.56-2.43) |
| ***FAS* c.-1378G>A *+FAS* c.-671A>G** |  |  |  |  |  |  |  |
| GG + AA | 58/78 |  | Reference |  | 63/78 |  | Reference |
| GA or AA + AG or GG | 2/2 | **0.01** | **6.84 (1.56-29.95)** |  | 2/2 | 0.06 | 3.81 (0.90-15.97) |
| GG or GA + AA or AG | 20/29 |  | Reference |  | 20/29 | 0.51 | Reference |
| AA + GG | 19/24 | 0.75 | 1.10 (0.59-2.07) |  | 20/24 |  | 1.22 (0.66-2.28) |
| ***FAS* c.-1378G>A + *FASL* c.-844C>T** |  |  |  |  |  |  |  |
| GG + TT |  |  | NE |  |  |  | NE |
| GA or AA + CT or CC |  |  |  |  |  |  |  |
| GG or GA + TT or CT | 17/25 |  | Reference |  | 21/25 | 0.67 | Reference |
| AA + CC | 17/22 | 0.87 | 1.05 (0.53-2.07) |  | 18/22 |  | 1.14 (0.60-2.15) |
| ***FAS* c.-671A>G + *FASL* c.-844C>T** |  |  |  |  |  |  |  |
| GG or TT | 47/64 |  | Reference |  | 64/77 |  | Reference |
| AG or AA + CT or CC | 7/8 | 0.27 | 1.56 (0.70-3.48) |  | 1/2 | 0.45 | 1.35 (0.61-3.00) |
| GG or AG + TT or CT | 6/9 |  | Reference |  | 7/9 | 0.58 | Reference |
| AA + CC | 42/52 | 0.67 | 1.20 (0.51-2.83) |  | 45/52 |  | 1.25 (0.56-2.78) |

N: number of patients; HR: hazard ratio; CI: confidence interval; ^*^: multivariate Cox analysis adjusted by tumor size, tumor stage and N; NE: not evaluated. Results with significant *P*-values are presented in bold letters. All variables with *P*-value ≤ 0.20 in univariate Cox regression analysis were included in multivariate analysis. All combinations with n ≤ 1 were not included in this table
